# Developmental programming: Differing impact of prenatal testosterone and prenatal bisphenol-A -treatment on hepatic methylome in female sheep

**DOI:** 10.1016/j.mce.2025.112655

**Published:** 2025-09-04

**Authors:** John Dou, Soundara Viveka Thangaraj, Yiran Zhou, Vasantha Padmanabhan, Kelly Bakulski

**Affiliations:** a Department of Epidemiology, University of Michigan, Ann Arbor, USA; b Department of Pediatrics, University of Michigan, Ann Arbor, USA

**Keywords:** DNA methylation, Liver, Prenatal exposures, Testosterone, Bisphenol A

## Abstract

Steroid hormones are integral to pregnancy and fetal development, regulating processes such as metabolism, inflammation, and immune responses. Excessive prenatal steroid exposure, through lifestyle choices or environmental chemicals, can lead to metabolic dysfunctions in offspring. The research focuses on how exposure to testosterone (T) and bisphenol A (BPA) affects the liver’s DNA methylome, a key component of the epigenome influencing long-term health. Using Suffolk sheep, the study involved two cohorts: one exposed to prenatal-T and the other to prenatal-BPA. Whole genome bisulfite sequencing was employed to map DNA methylation across over 22 million CpG sites. Regions with p-value<10^−4^ and a magnitude of difference of at least 5 % methylation between groups were considered differentially methylated. Results demonstrated substantial differential methylation in the liver tissues due to both treatments, with prenatal-T causing unique epigenetic modifications distinct from those induced by prenatal-BPA. Specifically, prenatal-T treatment resulted in 53 differentially methylated regions (DMRs), of which 31 were located in gene regions, including exons. Prenatal-BPA exposure led to 32 DMRs, with 22 associated with gene regions. These modifications were associated with genes governing lipid and glucose metabolism, potentially underlying the observed metabolic disruptions such as insulin resistance and dyslipidemia. Pathway analysis revealed that genes differentially methylated due to prenatal-T were involved in cellular organization, while those affected by prenatal-BPA were enriched in signal regulation pathways. The findings underscore how prenatal exposure to steroid excess and steroid-mimics influence epigenetic landscapes, contributing to metabolic disease programming.

## 1. Introduction

Steroid hormones are major modulators of pregnancy and fetal development, playing a vital role in physiological processes like metabolism, inflammation and immune response. Steroid hormones also play a crucial role in the pathogenesis of metabolic dysfunction associated steatotic liver disease (MASLD) (Yang et al., 2021). Based on the developmental origins of health and disease theory (Barker et al., 1986), prenatal exposure to excess steroids can interfere with normal development and metabolic homeostasis, thus contributing to programming of metabolic diseases later in life (Cardoso et al., 2019). This exposure to excess could occur from medical conditions producing excess androgens like luteoma and hyperreactio luteinalis or from the inadvertent exposure to environmental chemicals with steroidogenic potential. Bisphenol A (BPA), a synthetic estrogen, is one such steroid mimic that is ubiquitously present in the environment and in the human population (Lehmler et al., 2018; Tagne-Fotso et al., 2024).

Prenatal treatment with native steroids like testosterone (T) and steroid-mimics like BPA could activate steroid receptors in the developing fetus, programming metabolic changes, characteristic of MASLD, in the offspring as evidenced in animal models. For example, prenatal treatment with excess T in sheep results in metabolic changes including disrupted insulin secretion (Carrasco et al., 2020; Hogg et al., 2011) dyslipidemia and peripheral insulin resistance in female offspring (Veiga-Lopez et al., 2013a). Similar features of metabolic dysfunction are programmed by prenatal-T excess in rats and mice as well (Roland et al., 2010; Yan et al., 2013). The impact of prenatal-BPA treatment on metabolic parameters is also well-documented (Abulehia et al., 2022). Briefly, prenatal treatment with BPA results in metabolic disruptions like insulin resistance and adipocyte hypertrophy in sheep (Veiga-Lopez et al., 2016), lower birthweight, adulthood obesity, impaired glucose and insulin homeostasis in mice (Susiarjo et al., 2015), increased plasma triglyceride levels and adipocyte density in rats (Lejonklou et al., 2017). Maternal early pregnancy BPA concentrations was also associated with markers of metabolic dysfunction like plasma adiponectin and leptin in children (Chou et al., 2011; Volberg et al., 2013).

The liver is the primary metabolic organ central to insulin and glucose regulation as well as lipid metabolism. Accordingly, animal studies have demonstrated that prenatal-T and -BPA treatments induce changes in liver pathology and gene expression, which could underlie the observed metabolic disruption. Specifically, studies using the precocial animal model, sheep, have shown hepatic lipid accumulation and lipotoxicity (Puttabyatappa et al., 2017a) along with hepatic transcriptional changes (Saadat et al., 2022) in female offspring prenatally exposed to excess T. In a parallel study, gestational treatment with BPA resulted in lipotoxicity, increased oxidative stress and transcriptomic changes in the liver of female offspring (Puttabyatappa et al., 2019, 2022; Veiga-Lopez et al., 2015a). Similar hepatic transcriptome changes due to prenatal-BPA treatment (Nguyen et al., 2020; Shu et al., 2019) have also been reported in other animal studies. These observations prompt further investigation into the mechanisms that mediates the long-term impact of a transient exposure to excess steroids, native (prenatal T model) or environmental (prenatal BPA model), during fetal life.

The DNA methylome, the complete set of DNA methylation sites in a cell, is an integral component of the epigenome, which is at the intersection of environment and tissue-specific regulatory networks. During development, DNA methylation patterns can be remodeled in response to maternal environmental factors and in some cases, reversed if those environmental conditions change, thereby providing flexibility in gene regulation. Conversely, genomic imprinting is marked by typically stable and inherited methylation patterns that dictate parent-specific gene expression. However, certain imprinted genes are particularly sensitive to environmental exposures, during critical developmental windows which suggests that DNA methylation serves as both a stable inheritance mechanism and a responsive element to environmental changes (Colwell et al., 2023). This duality is particularly evident in studies of prenatal exposure to endocrine disruptors (Bommarito et al., 2017) such as BPA (McCabe et al., 2020), phthalates (Miura et al., 2021) and per- and polyfluoroalkyl substances (Starling et al., 2020) which have been associated with cord blood DNA methylation in humans. These alterations in the methylome may mediate long-term effects on health. Epidemiological studies have demonstrated links between prenatal-BPA treatment, DNA methylation, and adverse metabolic outcomes such as low birth weight (Huang et al., 2021) and early childhood body mass index (Choi et al., 2020). Although DNA methylation is generally more stable compared to other epigenetic mechanisms like histone modification, non-coding RNA, chromatin remodeling, all of which play vital roles in gene regulation (Acharjee et al., 2023), it retains the capacity for dynamic remodeling in response to specific developmental and environmental cues. Thus, DNA methylation provides both a stable epigenetic memory and the flexibility necessary to adapt to environmental changes during development. In contrast to histone modifications and chromatin remodeling, which can be rapidly reversed (Rao et al., 2001; Bannister et al., 2011), DNA methylation constitutes a more persistent epigenetic mark, thereby functioning as an enduring molecular record of prenatal exposures (Bozack et al., 2024). The direct impact of DNA methylation on gene expression, along with its crucial role in establishing cell-type specific gene expression pattern during fetal development (Slieker et al., 2015) makes DNA methylation a particularly compelling focus for understanding how prenatal exposures translate into long term metabolic programming.

Understanding tissue-specific DNA methylome changes is crucial for elucidating the mechanisms underlying the direct effects of prenatal exposures. This focus is particularly relevant when considering the impact of various steroid hormones and their mimics, such as BPA, which play crucial roles in modulating fetal development and programming metabolic diseases. Consequently, it becomes essential to investigate the underlying epigenetic mechanisms. By examining how adverse prenatal hormone exposure affects liver-specific DNA methylation and transcriptomic changes, we can gain insights into how early life exposures influence long-term metabolic health outcomes. Using a precocial large animal model like sheep, which shares a developmental trajectory similar to humans, offers an effective approach to achieving this understanding. Sheep pregnancy closely resembles human pregnancy in terms of placental development and transfer of oxygen and nutrients to the fetus (Barry et al., 2008). Additionally, the developmental ontogeny of major organs, including brain, lungs, pancreas, hypothalamus, ovary and regulatory systems is similar between sheep and humans (Morrison et al., 2018; Padmanabhan et al., 2013), allowing insights gained from sheep pregnancy studies to be effectively translated to human perinatal care (Morrison et al., 2018). Sheep models have widely been used to study several human disorders that are difficult to recapitulate in small animal models (Cardoso et al., 2019; Padmanabhan et al., 2013; Banstola et al., 2022). This study aims to examine the effects of prenatal treatment to native steroids and steroid-mimics on the liver methylome, elucidating their role in metabolic dysfunctions. We hypothesized that prenatal treatment with excess T (a native aromatizable androgen and estrogen precursor) or BPA (an environmental estrogen-mimic) disrupts the hepatic methylome in a manner consistent with the observed transcriptomic and phenotypic metabolic outcomes in the offspring.

## 2. Methods

### 2.1. Animals

Suffolk sheep were maintained at the University of Michigan Sheep Research Facility (Ann Arbor, MI) where studies were conducted following the Institutional Animal Care and Use Committee of the University of Michigan approved protocol in accordance with the National Research Council’s Guide for the Care and Use of Laboratory Animals and the Animal Welfare Act. This study is reported in accordance with ARRIVE guidelines (Percie du Sert et al., 2020). This study involved two cohorts of sheep: Cohort 1: control and prenatal-T treated and Cohort 2: control and prenatal-BPA treated. Animals from both cohorts were kept together under the same conditions and given a similar maintenance diet to prevent obesity. Potential phytoestrogen exposure through the diet was consistent across the treatment groups as previously described (Manikkam et al., 2004).

### 2.2. Prenatal-T treatment

In Cohort 1, pregnant sheep were randomly assigned to control and prenatal-T treatment groups. The prenatal-T treated group received biweekly intramuscular injections of T propionate (~1.2 mg/kg; Millipore Sigma, St. Louis, MO) suspended in corn oil, from gestational day 30 to day 90. Intramuscularly administered testosterone has a half-life of 19–24 h, requiring biweekly injections (Fujioka et al., 1986; Shoskes et al., 2016). There was no vehicle treatment for the control animals, as a prior study showed no effects of corn oil in sheep (Veiga-Lopez et al., 2008). Five control and five prenatal-T treated sheep were randomly selected and used in the previous liver transcriptomic study (Saadat et al., 2022) were used in the current study, ensuring mother as the experimental unit. The phenotypic effects and changes in mRNA and non-coding RNA in prenatal-T treated offspring have been published earlier (Cardoso et al., 2016; Dou et al., 2023; Lu et al., 2016; Puttabyatappa et al., 2017b, 2018, 2020).

### 2.3. Prenatal-BPA treatment

In Cohort 2, pregnant sheep were randomly assigned to control and prenatal-BPA treatment groups. Control and treatment groups were administered daily, subcutaneous injections of vehicle (corn oil) and 0.5 mg/kg/day BPA (purity ≥99 %, catalog number 239658; Aldrich Chemical Co, Milwaukee, Wisconsin) solubilized in corn oil, respectively. BPA has a biological half-life of less than 6 h and is eliminated from the body in 24 h, warranting daily subcutaneous injections (Ramírez et al., 2023). This treatment generated mean BPA concentration of ~2.6 ng/ml in the umbilical artery (Veiga-Lopez et al., 2013b). This level falls within the reference range established by human biomonitoring studies (Gerona et al., 2013; Lee et al., 2018; Veiga-Lopez et al., 2015b). In this study, the same four control and prenatal-T treated sheep from our prior liver transcriptomic investigation were employed (Puttabyatappa et al., 2022). These animals were chosen randomly, maintaining the mother as the experimental unit. The phenotypic effects of prenatal-BPA treatment on the offspring along with the transcriptome analysis of coding and noncoding RNA from this cohort have been previously published (Veiga-Lopez et al., 2015a, 2016; Puttabyatappa et al., 2019; Dou et al., 2023).

### 2.4. Tissue collection

At the conclusion of the second breeding season (~21 months of age), female offspring in the prenatal-T cohort were subjected to ovariectomy to eliminate any confounding influences from varying steroid levels. Liver tissue samples were obtained during the artificially induced follicular phase, following established protocols (Puttabyatappa et al., 2017a). Early follicular phase estradiol levels were maintained using a subcutaneous estradiol implant, 1 cm in length (Goodman et al., 1981). To synchronize estrous cycle, two controlled internal drug-release implants containing progesterone (CIDR-G; InterAg, Hamilton, New Zealand) were placed subcutaneously to mimic luteal phase levels of progesterone. The progesterone implants were removed after 14 days, and four 30-mm estradiol implants were inserted to produce late follicular phase levels of estradiol. Animals were euthanized 18 h later during the late artificial follicular phase by a barbiturate overdose (Fatal Plus; Vortech Pharmaceuticals, Dearborn, MI). In the prenatal-BPA treated cohort, female offspring liver tissue samples were collected during the natural follicular phase after synchronization with two injections of PGF2α, 11 days apart. Samples were taken 27 h after the second PGF2α injection. From all animals, liver tissue was collected from the tip of the left lobe, flash-frozen and stored at −80 °C.

### 2.5. DNA extraction, library construction and sequencing

DNA was extracted from 30 μg of frozen liver tissue using AllPrep DNA/RNA kit (Qiagen, Germantown, MD) using manufacturer protocol. A total of 200 ng of DNA was used for each whole genome bisulphite sequencing (WGBS). Library preparation and WGBS were carried out at the University of Michigan, Epigenomics Core. Each sample was spiked-in with 0.5 % (w/w) of unmethylated Lambda DNA prior to library preparation, according to the ENCODE consortium’s guidelines (Consortium, 2012). DNA was fragmented to a 350bp peak on a Covaris S220 focused-ultrasonicator system. The samples were next processed for end-repair and A-tailing, before proceeding to the ligation of a truncated methylated adaptor at 16 °C overnight. The ligation products were cleaned with two rounds of Omega Bio-tek Mag-Bind TotalPure NGS beads. The ligated products were submitted to bisulfite treatment using Zymo’s EZ DNA Methylation kit, with the following protocol: 55 cycles of 95 °C for 30sec, 55 °C for 15 min. Samples were kept at 4 °C until cleanup. Final library amplification was done using the KAPA HiFi Uracil + polymerase and NEB’s dual indexing primers. A total of 10 cycles of amplification were used. The PCR products were then cleaned with Omega Bio-tek Mag-Bind TotalPure NGS beads, and the final libraries were quantitated using the Qubit High Sensitivity dsDNA kit. Quality of the libraries was assessed on Agilent’s 2200 TapeStation, using the High Sensitivity D1000 kit. Libraries were quantified using the KAPA Library quantitation kit and pooled for sequencing with a S4 flowcell for 200 cycles (paired end, 100 base pairs in length) on a NovaSeq6000 at the University of Michigan Advance Genomics Core.

### 2.6. DNA methylation sequence data pre-processing

FastQC (v0.11.8) was used to assess quality of reads (https://www.bioinformatics.babraham.ac.uk/projects/fastqc/). Trimming was done with TrimGalore (v0.4.5) (https://github.com/FelixKrueger/TrimGalore) with the parameters: adapter AGATCGGAAGAGC -e 0.1 –stringency 6 –length 20 –nextseq 20. Reads were aligned to the ARS-UI-Ramb-v2.0 sheep genome build using Bowtie2 (v2.3.4) (Langmead et al., 2012). DNA methylation was called using MethylDackel (v0.4.0) (parameters -d 5 -D 2000 –mergeContext) (https://github.com/dpryan79/MethylDackel).

### 2.7. Differential methylation testing

Data were analyzed in R statistical software (version 4.4.0). First, the DNA methylation sites (CpGs) with coverage of at least 10 reads in all samples was filtered. Analysis was done separately for the prenatal-T and the prenatal-BPA cohorts. To visualize general patterns in the methylation data, principal component analysis (PCA) was computed on the 10 % most variable CpGs determined by variance/std deviation, then samples plotted by principal components. The first three principal components were used to generate a three-dimensional PCA plot using plotly package in R (https://cran.r-project.org/web/packages/plotly/citation.html). Global DNA methylation was examined by computing the average methylation level across all CpGs for each sample and global differences assessed by comparing treatment group versus control group with a two-tailed *t*-test with unequal variances.

Next, differential methylated region analysis was conducted using the dmrseq package (Korthauer et al., 2019), with default parameters for determining candidate regions. The dmrseq package fits a generalized least squares regression model with nested autoregressive correlated error structure, and computes significance with a permutation test on a pooled null distribution. Since there were no regions that crossed the FDR<0.05 cutoff, for exploratory analysis, regions with raw p-values <10^−4^ and a magnitude of difference of at least 5 % methylation between groups were considered differentially methylated. For regions of interest, differentially methylated regions were plotted using dmrseq. A heatmap was generated with hierarchical clustering to visualize patterns in DNA methylation across samples among differentially methylated regions.

### 2.8. Pathway analysis

For pathway enrichment analysis, a more liberal threshold of p-value<10^−3^ was applied to determine genes of interest. Pathway enrichment tests for gene ontology terms was performed with the gprofiler2 package using “gSCS” correction method (Kolberg et al., 2020). Genes in whose regions contain a DMR with p-value<10^−3^ (Prenatal-T treated, n = 157; Prenatal-BPA treated, n = 258 genes) were tested for enrichment, with the background set as genes with a measured CpG in our WGBS data. Pathway enrichment was visualized using a dot plot.

### 2.9. Integrated analysis of DNA methylome and transcriptome data

The transcriptome (RNA sequencing) data for the same samples have been published previously for *in utero* prenatal-T treated (Saadat et al., 2022) and prenatal-BPA treated cohorts (Puttabyatappa et al., 2022). The current epigenome data was matched with the transcriptome data based on gene annotation. As an exploratory analysis for the relationship between methylation and gene expression, we expanded the analysis to all genes with candidate DMRs (p-value <0.01) and matching expression data. For genes that showed both differential expression (adjusted p-value <0.05, log2 fold change >0.5) and differential methylation (p-value <10^−4^), Pearson correlation coefficient and their significance were computed and visualized as a scatterplot using the ggplot2 package (https://cran.r-project.org/web/packages/ggplot2/citation.html).

### 2.10 . GPRASP1 ELISA assay

A sheep-specific quantitative sandwich ELISA kit (MyBioSource, San Diego, USA) with a reported sensitivity of 1.0 ng/ml and detection range of 3.12–100 ng/ml was used to measure GPRASP1 levels. Liver tissue extracts were prepared by homogenizing the frozen liver tissue in phosphate buffered saline solution with 10 mM EDTA. 50 μl of this extract was used to quantify the total quantity of GPRASP1 according to manufacturer’s instructions. The intra- and inter-assay coefficient of variation is <15 %. A two-tailed *t*-test with equal variances was used to assess the difference between Control and Pre-T groups using GraphPad Prism (10.5.0) (GraphPad software, Boston, USA).

### 2.11. Control comparison

DNA methylation in Control animals from the prenatal-T cohort was compared against Control animals from the prenatal-BPA cohort to assess if the pattern of vehicle administration (no vehicle treatment for in prenatal-T cohort and daily subcutaneous injections in prenatal-BPA cohort) had any effect. Differentially methylated regions were first calculated and visualized, followed by performance of pathway analysis, using approaches described above.

### 2.12. Data availability

WGBS methylation data is available on the Gene Expression Omnibus (GSE296249). Data on gene expression has been previously released (GSE190328, GSE178777). Code used in analysis is available on GitHub (https://github.com/bakulskilab/Sheep-Hepatic-WGBS-BPA-and-prenatal-T).

## 3. Results

### 3.1. Sample descriptives

The average number of sequencing reads per CpG site in each sample was between 21 and 28, except for one prenatal-T treated sample that was sequenced more deeply with mean coverage of 47.7 (Table 1, Supplementary Fig. 1).

### 3.2. Effect of prenatal-T treatment on liver epigenome

**Fig. 1.**
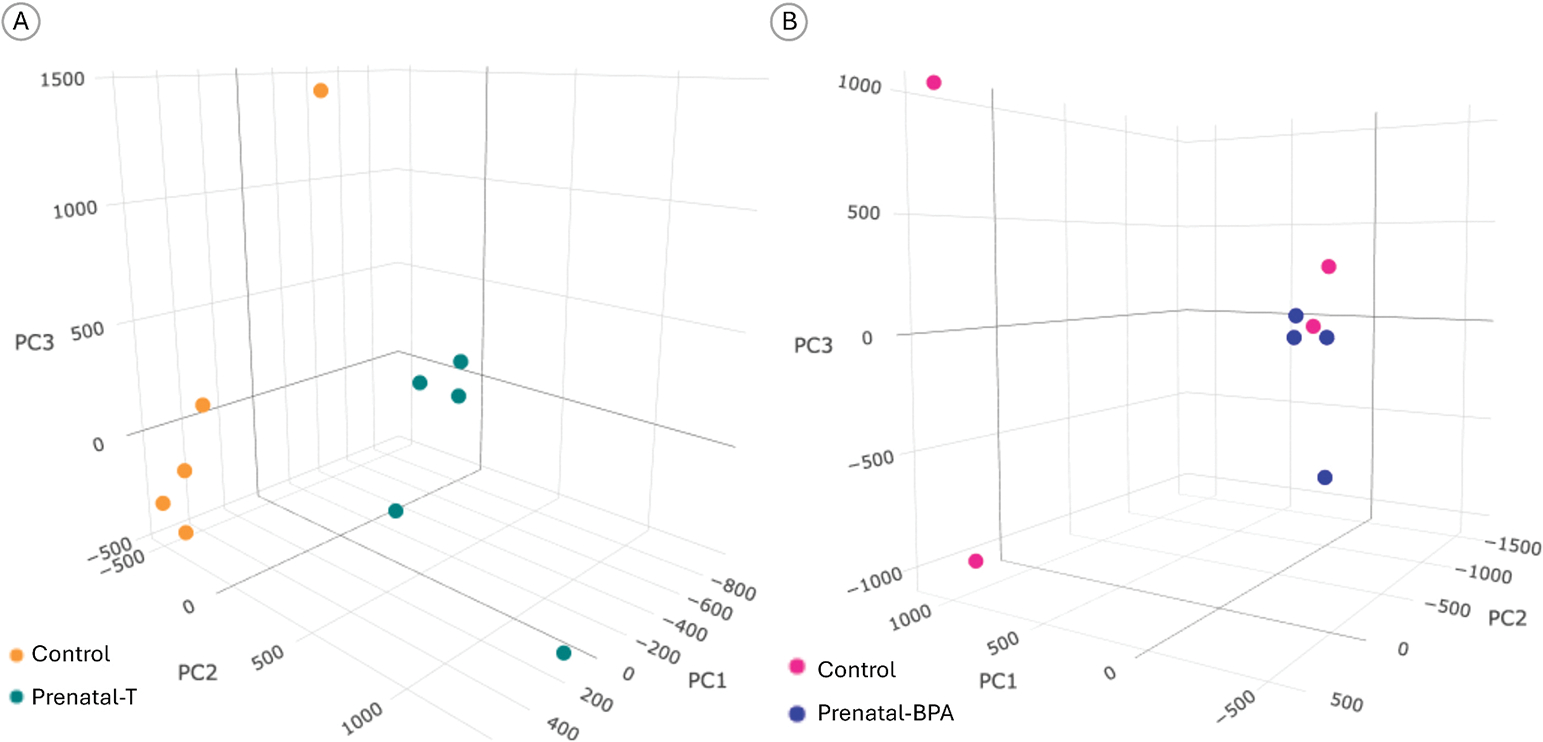
Principal Component Analysis of Differentially Methylated Regions (DMR). 3D Principal Component Analysis plots based on DMR in A) Prenatal-Testosterone and B) Prenatal-BPA treated female sheep liver. Principal components were calculated from the 10 % most variable CpGs.

After filtering (≥10 reads/CpG), 2,245,382 CpGs (of 22,453,820 total) were used for PCA, which separated prenatal-T and control sheep (Fig. 1A). Prenatal-T treatment did not alter global DNA methylation, with a difference of 0.37 % (95 % CI −0.81 %, 1.5 %).

**Fig. 2.**
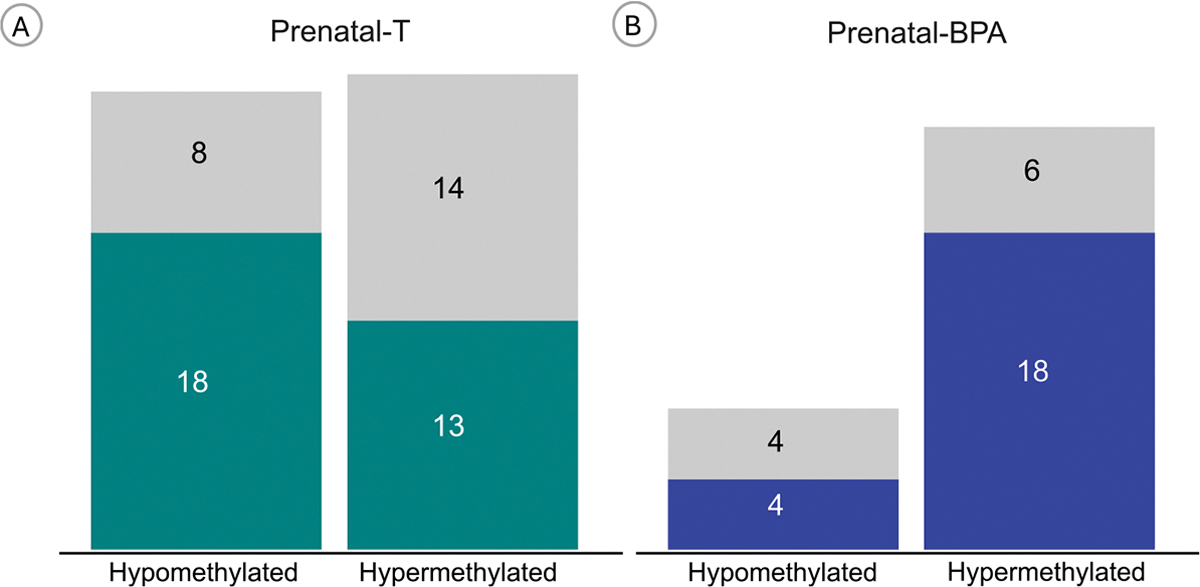
Differentially Methylated Region (DMR) Counts in Prenatal Exposures. Bar graph represents the number of hypomethylated or hypermethylated DMR counts in A) Prenatal-Testosterone or B) Prenatal-BPA treatment groups (X-axis). DMR annotated to a gene are indicated in teal.

**Fig. 3.**
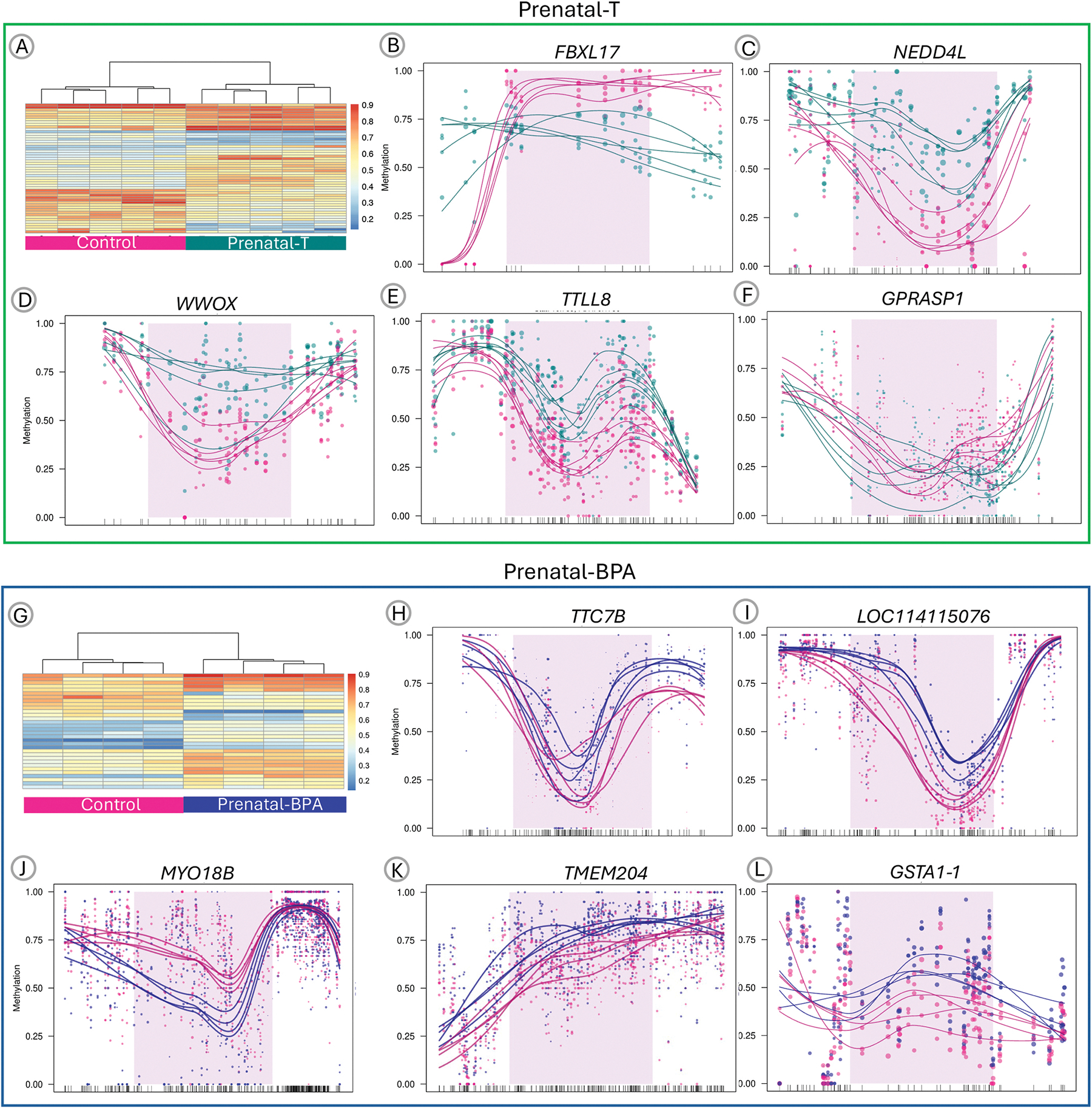
Differentially methylated region (DMR) in Prenatal-Testosterone and Prenatal-BPA treated sheep liver. Top panel - A) Heatmap depicting mean DNA methylation levels (%) in DMRs (rows) across samples (columns) for the Prenatal-Testosterone cohort. Color scale: red (100 % methylated) to blue (0 % methylated). Scatter plots of CpG methylation frequency (%) in the 5 most significant gene-annotated DMRs (Control: pink, Prenatal-T: teal): B) *FBXL17*, C) *NEDD4L*, D) *WWOX*, E) *TTLL8*, F) *GPRASP1*. Bottom panel - G) Heatmap depicting mean DNA methylation levels (%) in DMRs (rows) across samples (columns) for the Prenatal-BPA cohort. Color scale: red (100 % methylated) to blue (0 % methylated). Scatter plots of CpG methylation frequency (%) in the 5 most significant gene-annotated DMRs (Control: pink, Prenatal-BPA: blue): H) *TTC7B*, I) *LOC114115076*, J) *MYO18B*, K) *TMEM204*, L) *GSTA1–1. (For interpretation of the references to colour in this figure legend, the reader is referred to the Web version of this article.)*

Prenatal-T treatment was associated with 53 differentially methylated regions, 31 of which were annotated to a gene region (Fig. 2A, Table 2). Heatmap of the DMR showed clustering of the control and prenatal-T treated samples into separate groups (Fig. 3A). The top differentially methylated region was in an exon of *FBXL17* on chromosome 5, in which prenatal-T treated sheep had mean 24.1 % lower DNA methylation than control sheep (Supplementary Table S1). Scatter plots of the top 5 differentially methylated regions mapped to a gene are show in Fig. 3B to F and plots for all the differentially methylated regions are found in Supplementary File 1.

**Fig. 4.**
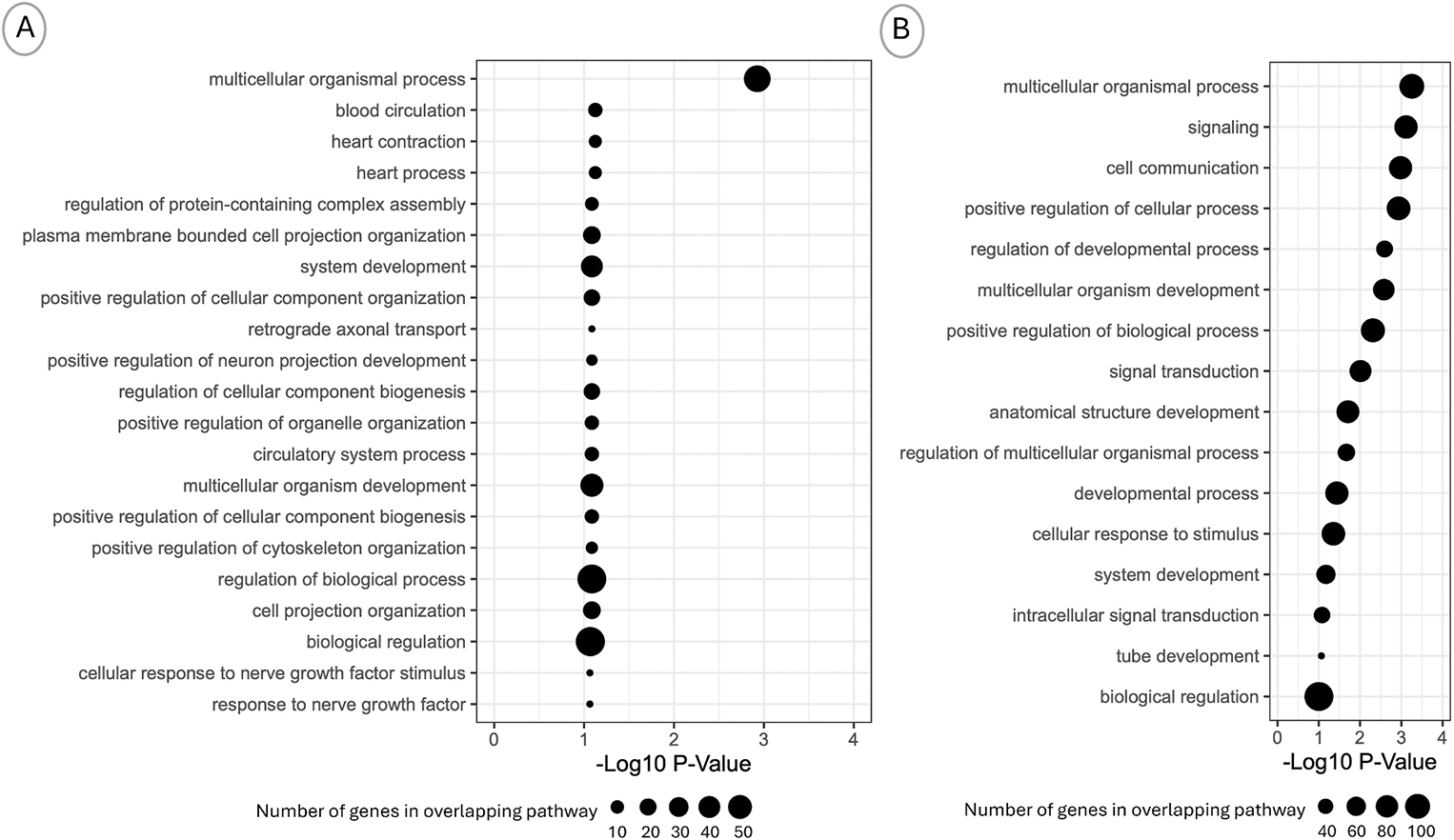
Biological Processes Associated with Prenatal Testosterone and BPA-Induced Methylation Changes. Graphs show enriched gene ontology biological processes in genes annotated to differentially methylated regions in A) Prenatal-Testosterone and B) Prenatal-BPA treatment groups. The X-axis represents the enrichment test −log10 (FDR p-value) and the Y-axis represent the biological processes. The circle size represents the number of differentially methylated genes in that biologic process.

Among genes annotated to prenatal-T differentially methylated regions (p-value <10^−3^, n = 157 genes), pathways involved in cardiac processes, and cellular organization and development were enriched (Fig. 4A, Supplementary Table S2).

**Fig. 5.**
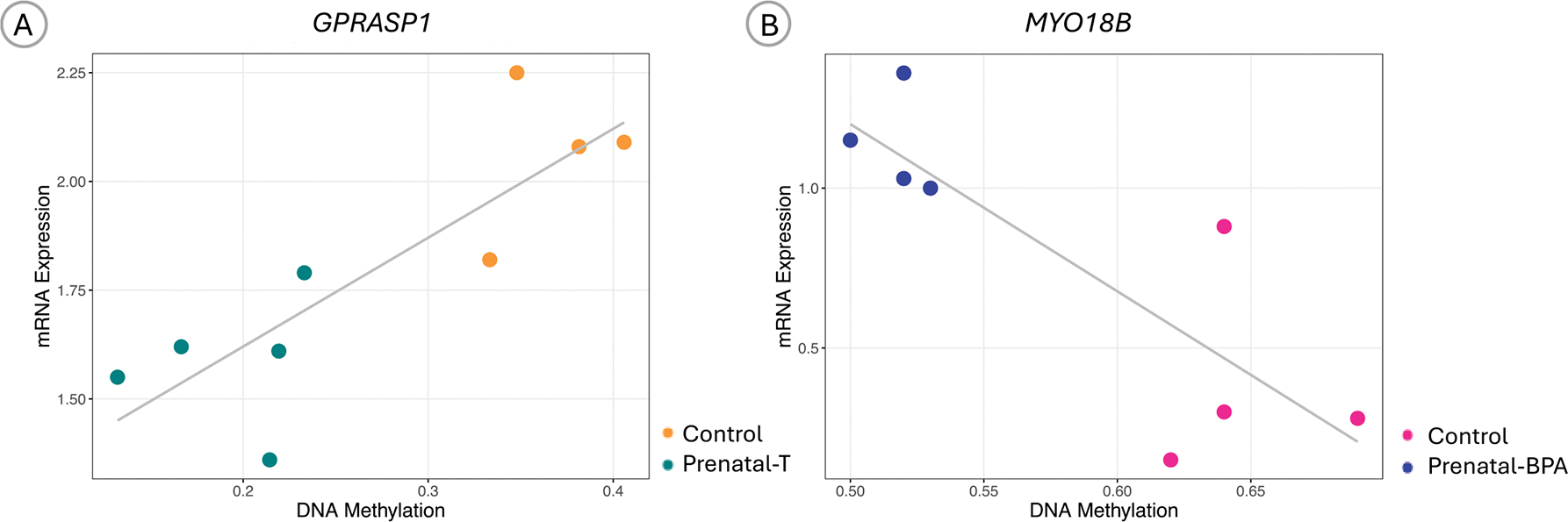
Correlation between mRNA expression and DNA methylation. Scatter plots showing the relationship between RNA log_2_ fold change and DNA methylation frequency coefficient for the differentially expressed genes A) *GPRASP1* in prenatal-Testosterone and B) *MYO18B* in prenatal-BPA treated sheep liver.

Of the 755 genes having a candidate DMR with p-value <0.01, 45 genes had expression levels that correlated with methylation at DMRs (Supplemental Table S3). The most correlated genes include *LOC105605861*, *NSD3*, *PAX7*, *AQP7*, and *IGSF21*. Of note, gene expression and methylation of *GPRASP1* was significantly positively correlated (Pearson’s r = 0.78, P-value = 0.02) (Fig. 5A), and in our previous study (Saadat et al., 2022) showed a differential expression (1.59-fold lower) in response to prenatal-T treatment. However, this relationship did not extend to the protein level, as GPRASP1 protein abundance remained unchanged between the Control and pre-T liver groups (P-value = 0.30) (Supplementary Fig. S2).

### 3.3. Effect of prenatal-BPA treatment on liver epigenome

There were 22,056,352 CpGs in the genome, after filtering loci with a coverage of at least 10 reads per CpG site for all samples. Of these, 2,205,635 CpGs with the most variance was used in PCA, which showed separation of control and prenatal-BPA treated sheep (Fig. 1B). Based on methylation in these regions, control and prenatal-BPA treated sheep clustered in separate groups. Prenatal-BPA treatment did not alter global DNA methylation, producing a difference of 0.21 % (95 % CI −0.79 %, 1.2 %).

Prenatal-BPA treatment resulted in 32 differentially methylated regions, of which 22 were annotated to a gene region (Fig. 2B–Table 3). Heatmap of the DMR showed clustering of the control and prenatal-BPA treated samples into separate groups (Fig. 3G). The top differentially methylated region had 34.2 % lower DNA methylation in prenatal-BPA treated sheep and was located in an intergenic region of chromosome 3 (Supplementary Table S4). Scatter plots of the top 5 differentially methylated regions mapped to a gene are shown in Fig. 3H to L and plots for all the differentially methylated regions are found in Supplementary File 2.

The differentially methylated genes (p-value <10^−3^, n = 258 genes) were enriched for pathways involved in cell communication (Fig. 4B–Supplementary Table S5).

There were 1291 genes having a candidate DMR with p-value <0.01 in relation to BPA exposure. Out of these, 127 genes had expression levels that correlated with methylation at DMRs (Supplemental Table S6). The most correlated genes include *TTC28*, *DENND2A*, *WBP2*, *TP53BP1*, and *PLEKHM1*. Notably, *MYO18B* exhibited both differential methylation and as demonstrated in our previous study (Puttabyatappa et al., 2022), differential expression in response to BPA exposure. Further, *MYO18B* gene expression was significantly correlated with its methylation (Pearson’s r = −0.8, P-value = 0.018) (Fig. 5B).

### 3.4. Model effect

To place the two exposure models in context, DNA methylation between the controls in the prenatal-T and the prenatal-BPA cohorts were compared. 3D-PCA of the Control samples showed tight clustering of the Controls from the prenatal-T cohort (Supplementary Fig. S3A). In differentially methylated region analysis, one region on chromosome 20 annotated to *GSTA1–1* differed between prenatal-T cohort and prenatal-BPA cohort controls (Supplementary Fig. S3B). No other regions reached the p-value<10^−4^ threshold (Supplementary Table S7).

Enriched pathways after relaxing the differentially methylated region criteria for pathway analysis (p-value <10^−3^, 17 genes) only included vascular endothelial growth factor receptor signaling (corrected p-value < 0.1). Due to the small number of genes reaching threshold for inclusion in pathway analysis, the observed number of genes overlapping with pathways was only 2 genes.

## 4. Discussion

**Fig. 6.**
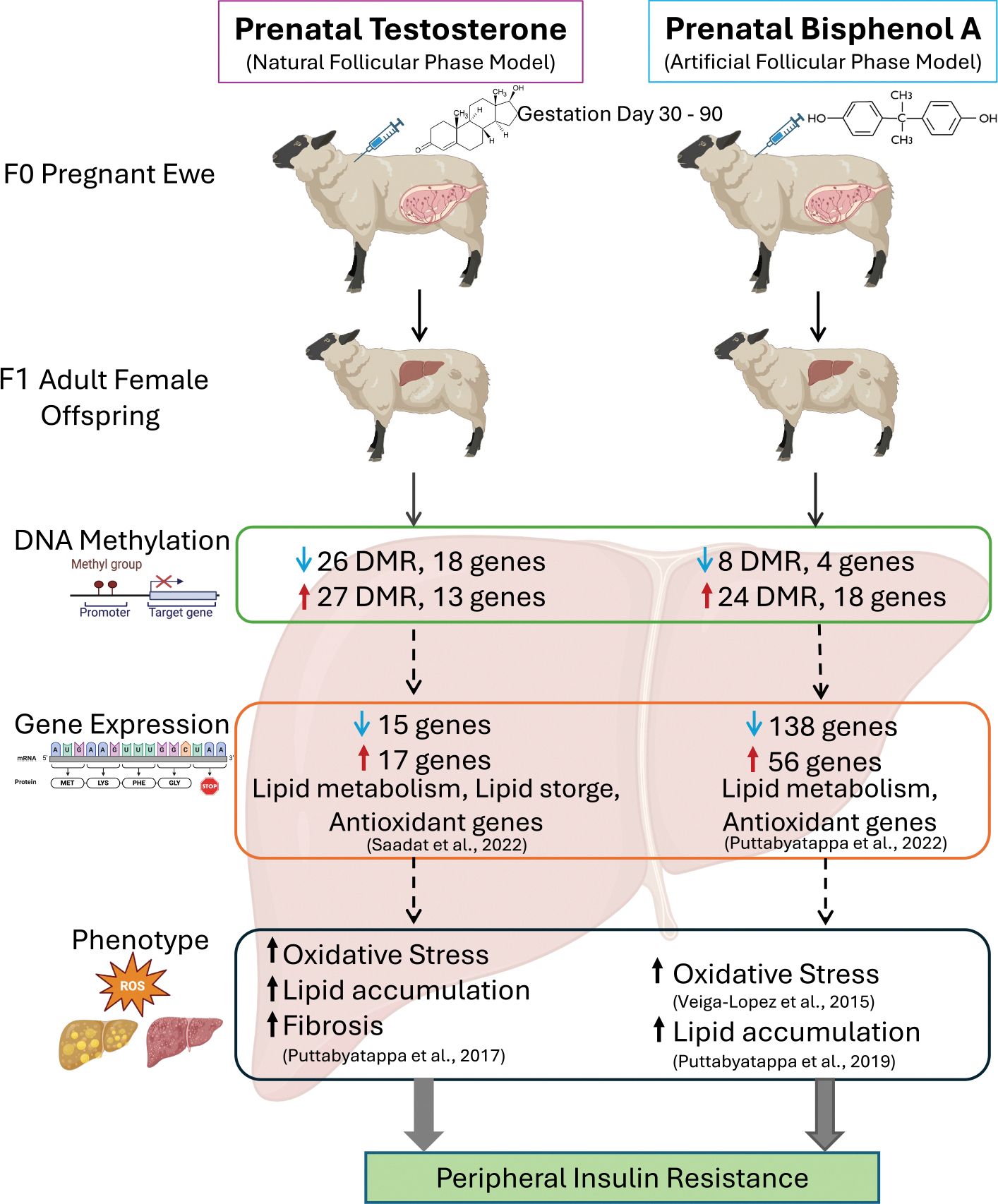
Summary of prenatal-Testosterone and -BPA exposure effects on liver. The figure integrates the DNA methylation changes with previously observed gene expression and phenotypic alterations, suggesting these models and potential contribution to insulin resistance development.

Our previous research demonstrated that prenatal exposure to endocrine disruptors, such as T, a native steroid, and BPA, an environmental steroid-mimic, can induce metabolic perturbations and alter liver gene expression in female offspring. As a key epigenetic mark established during development and maintained throughout life, DNA methylation is a strong candidate for mediating the effects of prenatal environmental exposures on long-term health outcomes. The liver’s central role in metabolism and detoxification makes the fetal liver particularly vulnerable to environmental exposures during critical windows of susceptibility, such as that used in this study. This study provides evidence of DNA methylation changes in the sheep liver induced by prenatal exposure to the native steroid, T and environmental steroid mimic, BPA. This is in line with our previous findings of metabolic perturbations and liver transcriptional changes in female sheep prenatally treated with T or BPA that were studied in parallel. A summary of these DNA methylation changes in relation to the observed phenotypic alterations like dyslipidemia and peripheral insulin resistance is represented in Fig. 6 and are discussed below.

### 4.1. Effect of prenatal-T treatment on liver epigenome

Given its profound effects on fetal development and metabolic outcomes documented from prior studies with this model (Puttabyatappa et al., 2017a) and other animal models (Bommarito et al., 2017; Abbott et al., 1998a; Mannerås et al., 2007; Padmanabhan et al., 2010), T indeed is a strong programming agent. Notably, early exposure to androgens can alter adult liver metabolism and signaling pathways, implicating the role of epigenetic mechanisms, such as DNA methylation in these metabolic disturbances (Hogg et al., 2011). Previous studies in female mice have demonstrated that exposure to masculinizing levels of T can lead to epigenetic programming at the gene promoter level in the liver (Dkhil et al., 2015). A possible mechanism of action is that T binds to androgen receptors (AR), triggering regulation of genes encoding DNA methyltransferases which drives methylation at promoter of genes. This is supported by previous study in this model showing prenatal T exposure increases the expression of the methyltransferase genes (*DNMT1* and *DNMT3A*) and histone acetylation at histone 3 lysine 9 (H3K9ac) and 27 (H3K27ac) in liver (Guo et al., 2020). Additionally, T can be aromatized to estradiol, in the liver, to activate estrogen receptors (ER) to recruit DNA methyltransferases, thus promoting methylation at specific gene locations (Kovacs et al., 2020; Norman et al., 2015). Building on these findings, this study using the sheep model has shown that prenatal-T treatment similarly programs DNA methylation in the liver of female offspring, specifically affecting genes crucial for lipid and glucose metabolism.

Methylation perturbations can have significant impact when it occurs in critical genes involved in lipid and glucose metabolism, influencing disease development. This study demonstrated that prenatal-T treatment resulted in hypomethylation of *GRB10*. *GRB10*, an imprinted gene that is a critical mediator in fetal programming of adult metabolic health, is known for its role in negatively regulating insulin signaling and implicated in endoplasmic reticulum stress-induced hepatic lipid regulation in mice (Luo et al., 2018; Smith et al., 2007). Several genes linked to oxidative stress displayed altered patterns following prenatal-T treatment. For instance, *PTGDS* which is related to endoplasmic reticulum stress and is highly expressed during MASLD progression (Gao et al., 2022) was hypomethylated. Likewise, genes that guard against lipid peroxidation and oxidative stress, such as *SLC8A3* (Chleilat et al., 2020) and *GSTA1–1* (Zhao et al., 1999) were also hypomethylated, suggestive of their increased expression. This aligns with previous research that demonstrated oxidative stress in the livers of these animals (Puttabyatappa et al., 2017b). In contrast, genes like *WWOX* that plays a critical role in lipid and glucose metabolism (Iatan et al., 2014) and *ADGRL3* that is a glucose receptor mediating energy and glucose homeostasis in mouse (Chhabra et al., 2024) were hypermethylated in response to prenatal-T treatment. Although the reduction in gene expression for these genes did not reach statistical significance (Saadat et al., 2022), it is possible that the hypermethylation led to inefficient transcription initiation or altered mRNA processing, ultimately resulting in lower levels of protein expression for these genes. If so, it would likely impair the function of these proteins, thereby disrupting lipid and glucose metabolism and potentially contributing to the increased lipid accumulation and oxidative stress observed in these animals (Puttabyatappa et al., 2019). Additionally, *CADM1* which affects insulin secretion and sensitivity in pancreas (Zhang et al., 2016), was hypomethylated in response to prenatal-T treatment. The specific functional implications of the other differentially methylated genes in liver are unknown.

*GPRASP1*, involved in lipid metabolism in sheep (Gunawan et al., 2021), was hypomethylated and it is the only differentially methylated gene that was also differentially expressed. However, this relationship did not extend to the protein level, suggesting that post-transcriptional regulation, translational efficiency, or protein stability mechanisms may buffer changes in mRNA expression, preventing them from being reflected in protein abundance. The relationship between DNA methylation and gene expression is complex and influenced by multiple factors, including histone modifications. Prior studies in these animals reported increased expression of the DNA methyltransferases *DNMT1* and *DNMT3B*, alongside increased histone acetylation at histone 3 lysine 9 (H3K9ac) and 27, in the liver of prenatal-T treated sheep (Guo et al., 2020). This interplay of epigenetic factors may explain the lack of a direct correlation between DNA methylation and gene expression observed in this study. The pathway analysis indicated strong enrichment of genes involved in cellular organization, development, and transport. This aligns with the established understanding of prenatal androgen excess’s impact on fundamental biological processes that shape tissue structure and function, potentially leading to altered development and organization at the cellular level (Siemienowicz et al., 2019).

### 4.2. Effect of prenatal-BPA treatment on liver epigenome

BPA is an estrogen-mimic which binds to the ER and alters recruitment of epigenetic modifiers to target genes (Kundakovic et al., 2013; Qin et al., 2020). This mechanism of action could contribute to a DNA hypomethylating effect on the epigenome of the offspring, as seen in mouse (Dolinoy et al., 2007). Early developmental exposure to BPA has been shown to cause dose-dependent changes in metabolic phenotype and liver DNA methylation, a finding well-established in animal models such as mice and rats (Shu et al., 2019; Kim et al., 2014; Ma et al., 2013). Rats prenatally exposed to BPA showed a decrease in global hepatic DNA methylation along with insulin resistance (Ma et al., 2013). Similarly, our previous findings in sheep reveal that prenatal-BPA treatment leads to ectopic lipid accumulation and insulin resistance in female offspring, highlighting similar epigenetic impacts (Puttabyatappa et al., 2019). This study explores if changes in liver DNA methylation may underlie these metabolic disturbances, emphasizing the potential mechanisms underlying BPA’s metabolic disruptions.

The gene *TTC7B*, associated with metabolic disorders like diabetic nephropathy (Khurana et al., 2023), was hypermethylated. *GSTA1–1*, a gene known for its role in protecting the liver against oxidative stress (Zhao et al., 1999), was hypermethylated in response to prenatal-BPA treatment, potentially undermining oxidative stress defense. A transmembrane protein gene, *TMEM204*, typically hypomethylated in MASLD patients (Abbott et al., 1998b; Murphy et al., 2013; Oh et al., 2024), was found to be hypermethylated, and *EFCAB11*, often downregulated in obese individuals with diabetes (Ganekal et al., 2023), showed similar hypermethylation in prenatal-BPA treated liver samples. The *FOXN3* gene, crucial for liver glucose metabolism, was hypermethylated, possibly contributing to the insulin resistance seen in these animals (Veiga-Lopez et al., 2016). Hypermethylation of the growth hormone receptor gene (*GHR*), critical for lipid metabolism and insulin-glucose homeostasis (Liu et al., 2016; Vazquez-Borrego et al., 2023), might explain the observed lipid accumulation and insulin resistance. Similarly, the *MIR-27A* gene, linked to insulin resistance and hepatic lipid metabolism (Chen et al., 2019; Zhang et al., 2017), was hypermethylated, predisposing these animals to the perturbed metabolic phenotype. Another hypomethylated gene is *VAV2*, a guanine nucleotide exchange factor that regulates glucose-stimulated insulin secretion in islet β-cell and metabolic homeostasis in muscles (Lv et al., 2020; Rodriguez-Fdez et al., 2020) but its role in liver is unknown. *ITGA7*, important for insulin and glucose metabolism, Taylor (2018) showed hypermethylation, as did *PLXND1*, a therapeutic target in liver fibrosis and associated with adipocyte lipolysis (Strawbridge et al., 2016; Yagai et al., 2014), further linking these epigenetic changes to the metabolic disruptions observed due to prenatal-BPA treatment (Veiga-Lopez et al., 2016). The hypomethylated gene *MYO18B* is the only gene that exhibited a significant correlation between its methylation and gene expression. This gene is involved in calcium signaling (Zhao et al., 2020) and its methylation in peripheral blood DNA is associated with fatty liver index (Ko et al., 2022), although its role in liver functions is unclear. The pathway analysis indicated strong enrichment of genes involved in development processes and response to stimulus. This is in line with BPA’s known role in altering liver development (DeBenedictis et al., 2016) and inducing an immune response (Yan et al., 2008), based on studies in mouse.

### 4.3. Comparison between prenatal-T and prenatal-BPA treatment effects – model effect

The rationale for comparing prenatal T and BPA treatments is twofold: both treatments have been shown to impact hepatic function and promote insulin resistance (Puttabyatappa et al., 2017a, 2017b, 2019, 2022; Saadat et al., 2022) and, both may act through ER-dependent mechanisms, raising the possibility of shared pathways of developmental programing. In the pre-T model, T can be aromatized to estrogen, enabling activation of ER signaling pathways in addition to AR pathways. In the prenatal-BPA model, BPA acts as an estrogen-mimic and can directly bind and activate ER. Thus, both treatments may drive epigenetic programming in the liver through ER-mediated processes, supporting a direct comparison aimed at investigating both unique and convergent effects on fetal development and epigenetic regulation.

Despite these mechanistic overlaps, T and BPA produce markedly different DNA methylation signatures in the liver. Prenatal-T treatment results in a nearly equal distribution of hypo- and hypermethylated hepatic genes, with affected genes enriched in pathways governing cellular organization, development, and transport, indicative of a broad systemic response. In contrast, prenatal-BPA treatment predominantly induces hypermethylation, impacting genes enriched in developmental processes, cellular signaling, regulation, and communication pathways, which are fundamental to liver function. These findings suggest compound-specific mechanisms: T influences the epigenome via both AR and ER (post-aromatization), while BPA modulates DNA methylation primarily through ER engagement (AlOgayil et al., 2021).

Methodologically, a key distinction between the two models lies in the treatment paradigm. The prenatal-T model simulates an artificial follicular phase by ovariectomizing animals and administering steroid replacements to mimic the hormone conditions of ovary-intact animals. In the T model, T with a relatively long half-life is delivered via bi-weekly intramuscular injections without any vehicle treatment for the controls. Conversely, BPA, with its short half-life, requires daily subcutaneous injections, with the control group receiving daily vehicle treatments. The rationale for the different exposure schemes is rooted in the distinct pharmacokinetic profiles of testosterone and BPA, particularly their half-lives and rates of biological clearance. Importantly, methylation profiling of control livers from both cohorts revealed no differences, except for a single gene (*GSTA1–1*), validating cohort comparability and supporting that observed methylation changes are due to treatment rather than methodological artifacts. This also aligns with our previous findings indicating no phenotypic changes in sheep due to vehicle treatment (Veiga-Lopez et al., 2008). Therefore, despite differences in dosing regimens, directly comparing these two exposures provides critical insights into both shared and distinct mechanisms of developmental epigenetic reprogramming in the liver.

### 4.4 . Strengths and limitations

By investigating the developmental impact of both T (a native steroid) and BPA (an environmental mimic) in a well-characterized sheep model, the study offers insights into different mechanisms of endocrine disruption and their epigenetic and phenotypic consequences, underlining the importance of prenatal environment in the long-term health of an individual. These findings in a large, precocial animal model with a developmental trajectory similar to humans is of translational relevance to human health and disease prevention (Gonzalez-Bulnes et al., 2017). In this study, using whole genome bisulfite sequencing, a “gold standard” genome-wide DNA methylation detection approach (Kernaleguen et al., 2018), we were able to quantify DNA methylation levels at over 22 million CpG sites, out of an estimated 28 million in the sheep genome (Doherty et al., 2014), with a high sequencing read depth (average per sample >21 per CpG site). This allowed for comprehensive genome-wide discovery analysis. Future follow-up studies using various DNA methylation detection approaches are expected to identify some overlapping CpG sites examined in this study, given the extensive coverage achieved with whole-genome bisulfite sequencing. Another strength of this study is correlating the DNA methylation changes with gene expression changes and metabolic outcomes in the same animals, allowing for a deeper understanding of the interplay between DNA methylation and the transcriptome in relation to the phenotype.

The finding that DNA methylation findings do not reach genome-wide statistical significance when accounting for over 22 million CpG sites tested makes it difficult to draw strong conclusions about the role of these changes in the phenotype under study. This could be an effect of the small sample size that reduces the power of this study to detect true differences. Large animal models show high genetic variability and DNA methylations are also highly variable, both within and between individuals (Bergstedt et al., 2022; Chatterjee et al., 2021). This variability can make it difficult to detect statistically significant differences, especially when the effect size is small. The majority of DMRs in this study associated with genes overlapped exon regions. It is important to note that the current functional annotation of the sheep genome is limited, which restricts detailed mapping and interpretation of DMRs in relation to other genomic features such as promoters, introns, or enhancers. As further annotation and improved genome assemblies become available for sheep, future analyses will enable a more comprehensive assessment of the genomic context of DMRs and their potential regulatory impact. The combination of modest DNA methylation effect sizes and a limited sample size in our study constrains the statistical power for rigorous integrative analyses of the methylome and transcriptome, thereby diminishing the reliability of detecting true biological associations following correction for multiple comparisons. Similarly, the lack of validated sheep-specific antibodies is a limitation for protein quantification in non-model species such as sheep, and we hope future development of such tools will enable comprehensive multi-omic validation. This study focused only on female offspring and including male offspring would have better distinguished and differentiated the androgenic effect of T against the estrogenic effect of BPA. While several genes were identified with differential methylation, the overall functional implications of many of these changes remain unclear without additional functional studies. Future studies to incorporate other epigenetic mechanisms such as histone modifications and non-coding RNAs will enhance our understanding of the overall epigenetic response to environmental challenges.

## 5. Conclusion

Prenatal exposure to steroid-excess and environmental steroid mimics is associated with DNA methylation in female offspring liver which could precede the hepatic transcriptional changes and the metabolic phenotype seen in these animals. Several of the DNA methylation changes that was seen in genes linked to prenatal exposure involved genes related to glucose, insulin or lipid metabolism, contributing critical knowledge into the mechanisms of metabolic disruptions induced by prenatal exposure to androgen and BPA.

## Supplementary Material

Supplementary File 1

Supplementary Table S5

Supplementary Table S6

Supplementary Table S7

Supplementary File 2

Supplementary Figure S1

Supplementary Figure S2

Supplementary Figure S3

Supplementary Table S1

Supplementary Table S2

Supplementary Table S3

Supplementary Table S4

## Figures and Tables

**Table 1:** Sample metrics from DNA methylation analysis.

Sample	Treatment Group	Number of Raw Reads	Percent Duplicated Reads	CpG Coverage Pre-Filter	CpG Coverage Post-Filter	Mean CpG Coverage by Treatment Group

**Cohort 1**						
S131	Control	622681064	9.41	24.54	25.94	25.5
S132	Control	668830953	9.88	25.80	27.19	
S179	Control	583531875	10.04	22.48	23.84	
S201	Control	585504484	10.73	22.66	23.92	
S98	Control	664992696	9.63	25.28	26.81	
S185	Prenatal-T	551149583	9.69	21.75	22.91	27.2
S233	Prenatal-T	458888443	9.08	18.33	19.43	
S255	Prenatal-T	566282549	9.62	21.49	22.76	
S48	Prenatal-T	1165690984	10.61	44.80	47.70	
S63	Prenatal-T	569648514	9.87	21.89	23.21	
**Cohort 2**						
S902	Control	645450631	9.33	23.53	24.82	24.9
S908	Control	661869704	9.88	25.31	27.09	
S924	Control	534560082	8.96	20.35	21.81	
S928	Control	678933327	10.72	24.10	25.75	
S910	Prenatal-BPA	693947379	11.03	26.69	28.08	24.7
S918	Prenatal-BPA	659995834	11.64	25.16	26.46	
S977	Prenatal-BPA	588798877	9.96	21.48	22.75	
S984	Prenatal-BPA	530810505	10.09	20.45	21.50	

**Table 2:** Differentially methylated regions (DMR) corresponding to genes in prenatal-T treated sheep liver.

Gene Symbol	Chromosome	Length of DMR	nCpG	P value	Q value	Mean Difference

*FBXL17*	chr5	368	10	3.00 × 10^−6^	0.17	−0.24
*NEDD4L*	chr23	1137	19	3.00 × 10^−6^	0.17	0.27
*GPRASP1*	chrX	1017	29	1.20 × 10^−5^	0.17	−0.15
*TTLL8*	chr3	675	27	1.20 × 10^−5^	0.17	0.21
*WWOX*	chr14	712	18	1.20 × 10^−5^	0.17	0.23
*LOC132660002*	chr6	642	15	1.50 × 10^−5^	0.18	−0.15
*ARHGAP35*	chr14	954	21	1.70 × 10^−5^	0.18	−0.14
*GSN*	chr2	1019	31	1.90 × 10^−5^	0.18	0.16
*TRABD*	chr3	583	18	2.10 × 10^−5^	0.18	0.19
*LOC132657210*	chr9	1952	18	2.60 × 10^−5^	0.18	−0.23
*ENTPD6*	chr13	867	17	3.40 × 10^−5^	0.18	0.16
*SLC8A3*	chr7	101	8	3.50 × 10^−5^	0.18	−0.19
*RSPH6A*	chr14	830	50	3.80 × 10^−5^	0.18	−0.20
*LOC114112674*	chr2	1037	52	3.90 × 10^−5^	0.18	0.13
*PKP2*	chr3	5518	32	4.40 × 10^−5^	0.18	−0.20
*LOC114117762*	chr14	669	28	4.60 × 10^−5^	0.18	−0.22
*LOC114114643*	chr4	750	12	5.00 × 10^−5^	0.18	−0.20
*GRB10*	chr4	540	15	5.30 × 10^−5^	0.18	−0.18
*LOC132659363*	chr2	493	29	5.30 × 10^−5^	0.18	−0.28
*RIN2*	chr13	236	11	6.50 × 10^−5^	0.19	0.19
*SEC31A*	chr6	427	25	6.60 × 10^−5^	0.19	0.18
*TSSK1B*	chr17	606	31	7.00 × 10^−5^	0.19	0.21
*TRAPPC3L*	chr8	584	31	7.20 × 10^−5^	0.19	−0.09
*SMAP1*	chr9	1117	13	7.30 × 10^−5^	0.19	−0.15
*CADM1*	chr15	612	11	7.40 × 10^−5^	0.19	−0.33
*ZNF71*	chr14	2257	80	7.40 × 10^−5^	0.19	−0.19
*GSTA1-1*	chr20	1474	21	8.60 × 10^−5^	0.20	−0.15
*ADGRL3*	chr6	2399	13	9.00 × 10^−5^	0.20	0.21
*PTGDS*	chr3	1129	41	9.00 × 10^−5^	0.20	−0.19
*TCHP*	chr17	361	22	9.20 × 10^−5^	0.20	0.09
*MYO16*	chr10	499	18	9.80 × 10^−5^	0.21	0.21

**Table 3:** Differentially methylated regions (DMR) corresponding to genes in prenatal-BPA treated sheep liver.

Gene Symbol	Chromosome	Length of DMR	nCpG	P value	Q value	Mean Difference

*TTC7B*	chr7	1041	34	8.00 × 10^−6^	0.40	0.17
*LOC114115076*	chr5	2736	61	1.1 × 10^−5^	0.40	0.16
*MYO18B*	chr17	4508	61	1.40 × 10^−5^	0.40	−0.13
*TMEM204*	chr24	2076	95	2.30 × 10^−5^	0.40	0.11
*GSTA1-1*	chr20	2800	29	2.40 × 10^−5^	0.40	0.15
*INKA1*	chr19	836	36	2.70 × 10^−5^	0.40	0.16
*EFCAB11*	chr7	760	20	3.90 × 10^−5^	0.40	−0.23
*MAN2A1*	chr5	816	27	5.20 × 10^−5^	0.40	0.19
*FOXN3*	chr7	525	11	5.30 × 10^−5^	0.40	0.22
*GHR*	chr16	6131	55	6.10 × 10^−5^	0.40	0.13
*LOC121819803*	chr6	531	12	6.10 × 10^−5^	0.40	−0.24
*NFATC1*	chr23	469	20	6.40 × 10^−5^	0.40	0.17
*MIR27A*	chr5	5299	160	7.30 × 10^−5^	0.40	0.10
*VAV2*	chr3	742	28	7.60 × 10^−5^	0.40	0.15
*TMEM132C*	chr17	1356	12	8.50 × 10^−5^	0.40	−0.31
*GPR182*	chr3	4619	100	8.70 × 10^−5^	0.40	0.10
*DLEU7*	chr10	644	36	9.00 × 10^−5^	0.40	0.12
*MGRN1*	chr24	895	42	9.00 × 10^−5^	0.40	0.17
*ITGA7*	chr3	483	17	9.20 × 10^−5^	0.40	0.13
*LOC105604543*	chr23	796	12	9.20 × 10^−5^	0.40	0.21
*TMEM131L*	chr17	615	16	9.30 × 10^−5^	0.40	0.17
*PLXND1*	chr19	1583	82	9.90 × 10^−5^	0.40	0.12

## Data Availability

The link to data and codes used in this study has been shared in the manuscript in the Methods section.

## Appendix A. Supplementary data

Supplementary data to this article can be found online at https://doi.org/10.1016/j.mce.2025.112655.
