# Supplementary material for: Developmental programming: Differing impact of prenatal testosterone and prenatal bisphenol-A -treatment on hepatic methylome in female sheep
Source: Mol Cell Endocrinol. Author manuscript; Available in PMC 2025 Dec 1. (PMC12668269; doi:10.1016/j.mce.2025.112655)
Supplement: Supplementary File 1 [file NIHMS2117496-supplement-Supplementary_File_1.pdf]

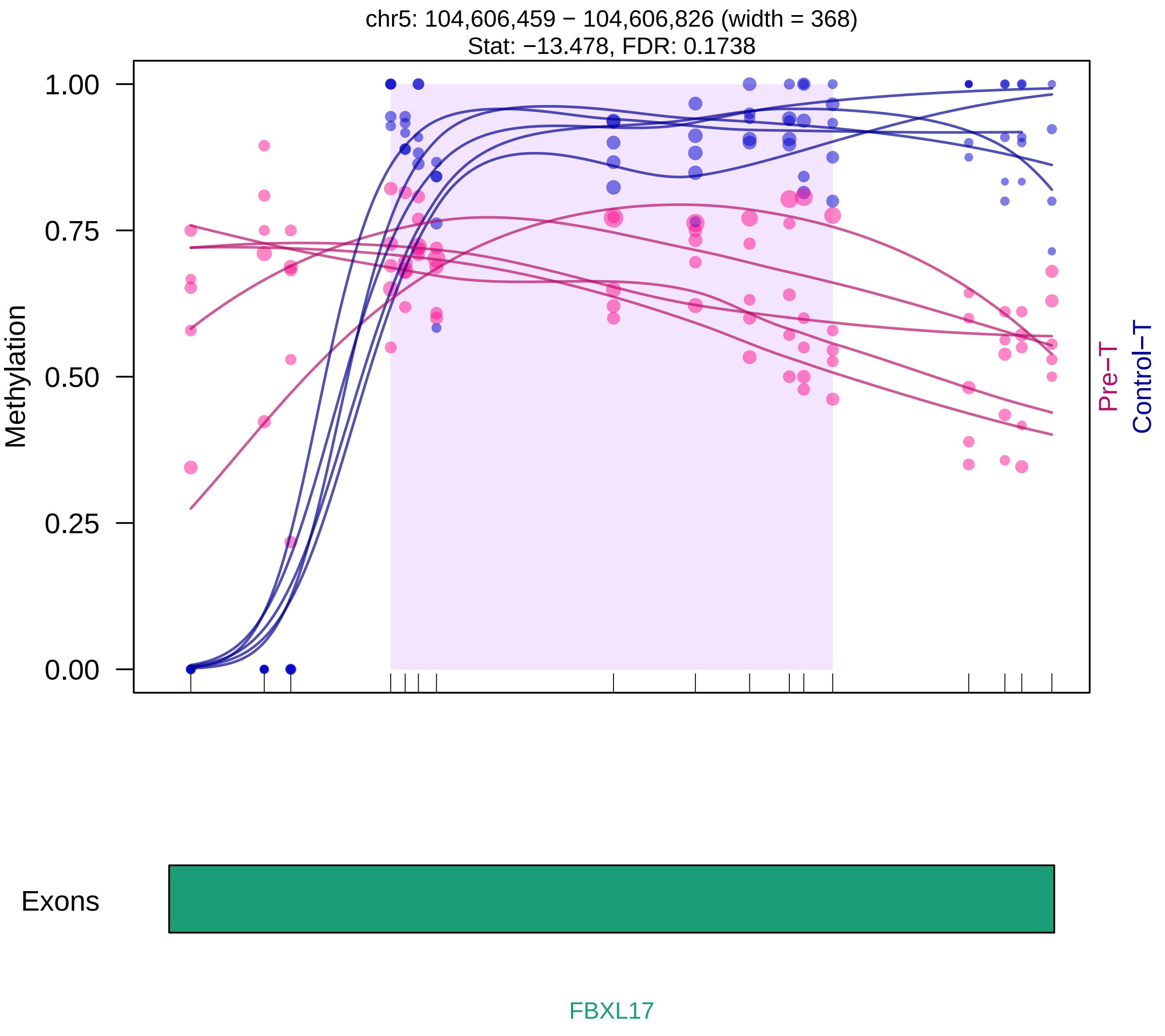

chr5: 104,606,459 − 104,606,826 (width = 368)
Stat: −13.478, FDR: 0.1738
Methylation
1.00
0.75
0.50
0.25
0.00
Pre−T
Control−T
Exons
FBXL17

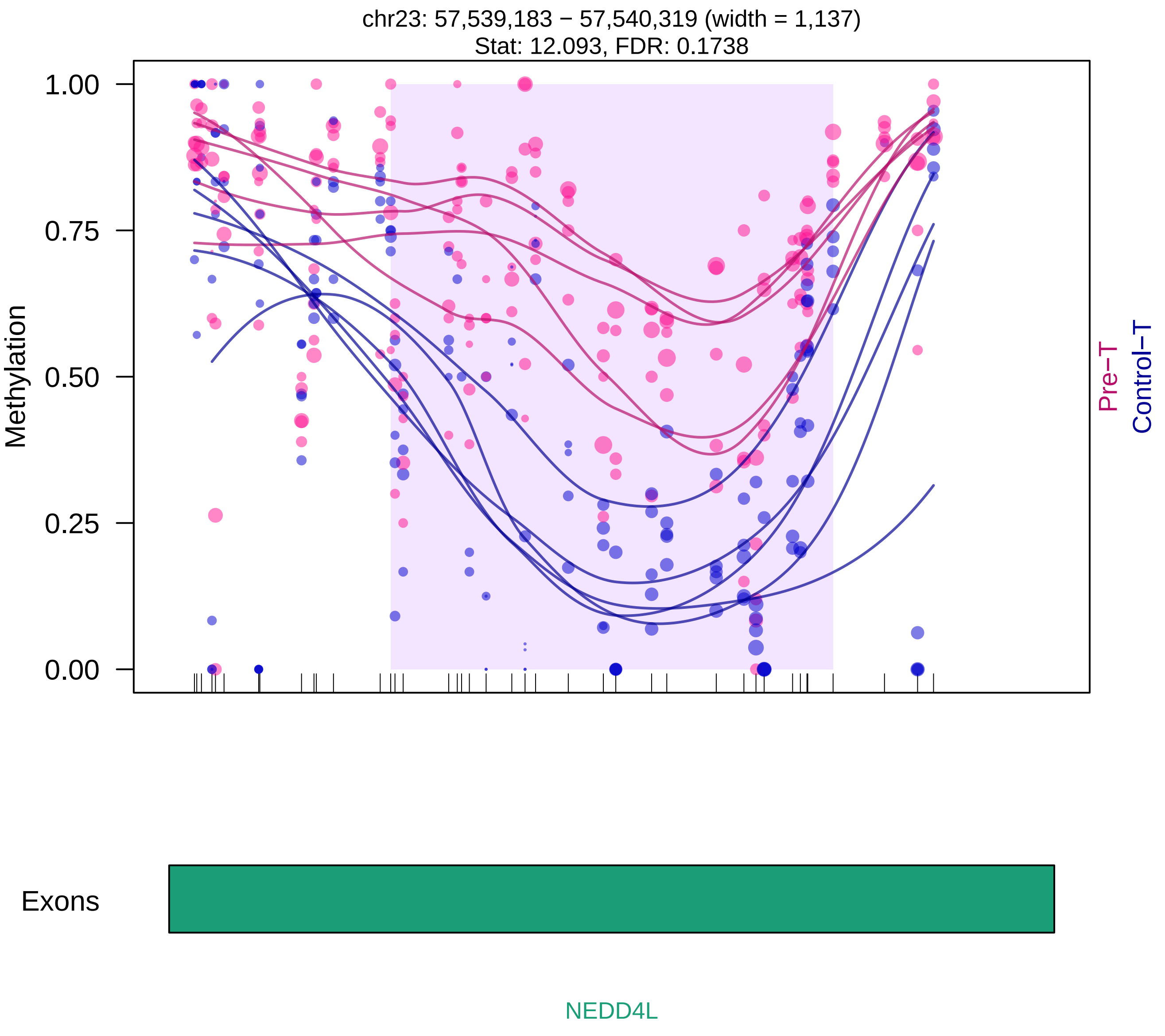
chr23: 57,539,183 − 57,540,319 (width = 1,137)
Stat: 12.093, FDR: 0.1738
1.00
0.75
0.50
0.25
0.00
Methylation
Pre−T
Control−T
Exons
NEDD4L

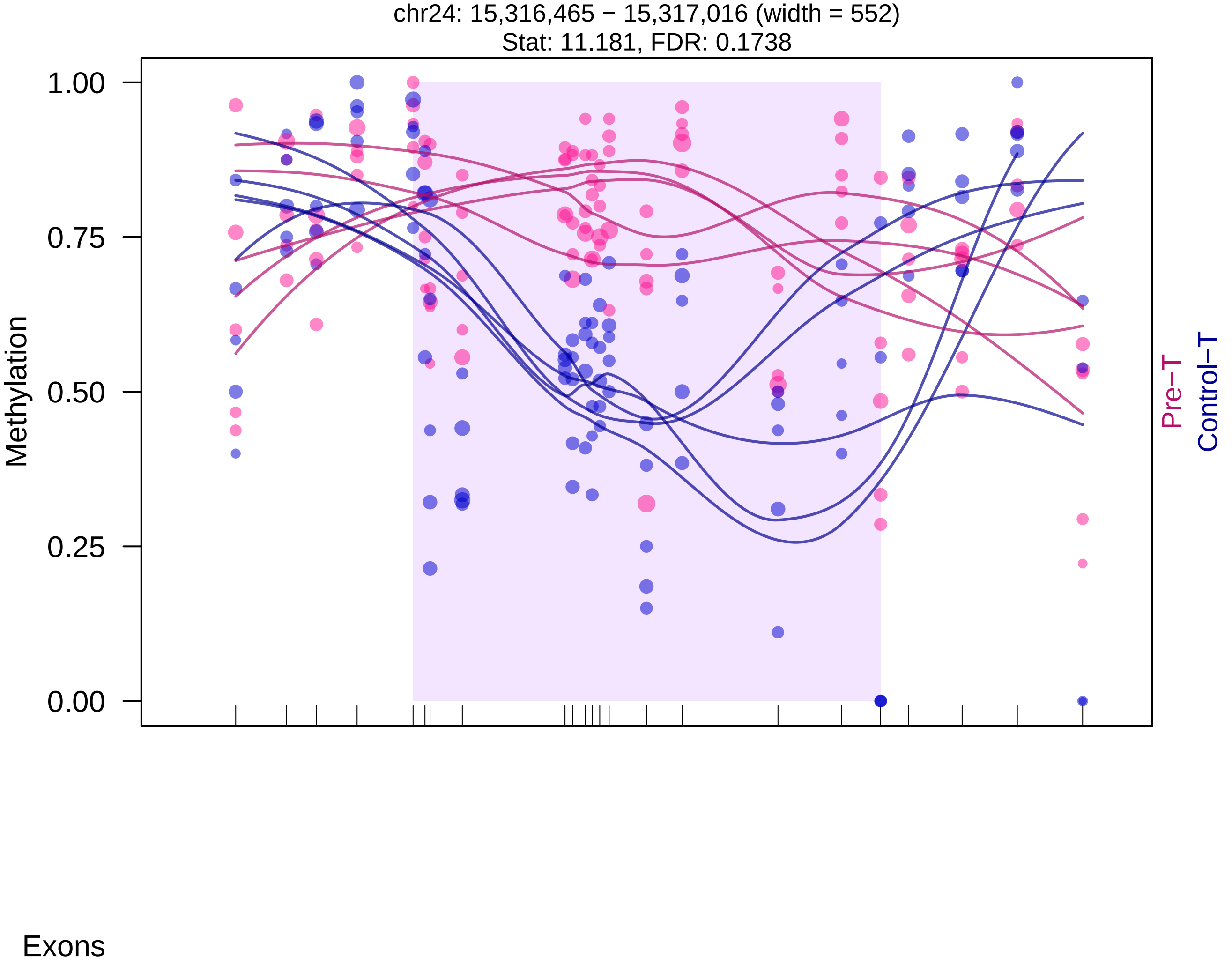
chr24: 15,316,465 − 15,317,016 (width = 552)
Stat: 11.181, FDR: 0.1738
Methylation
1.00
0.75
0.50
0.25
0.00
Pre−T
Control−T
Exons

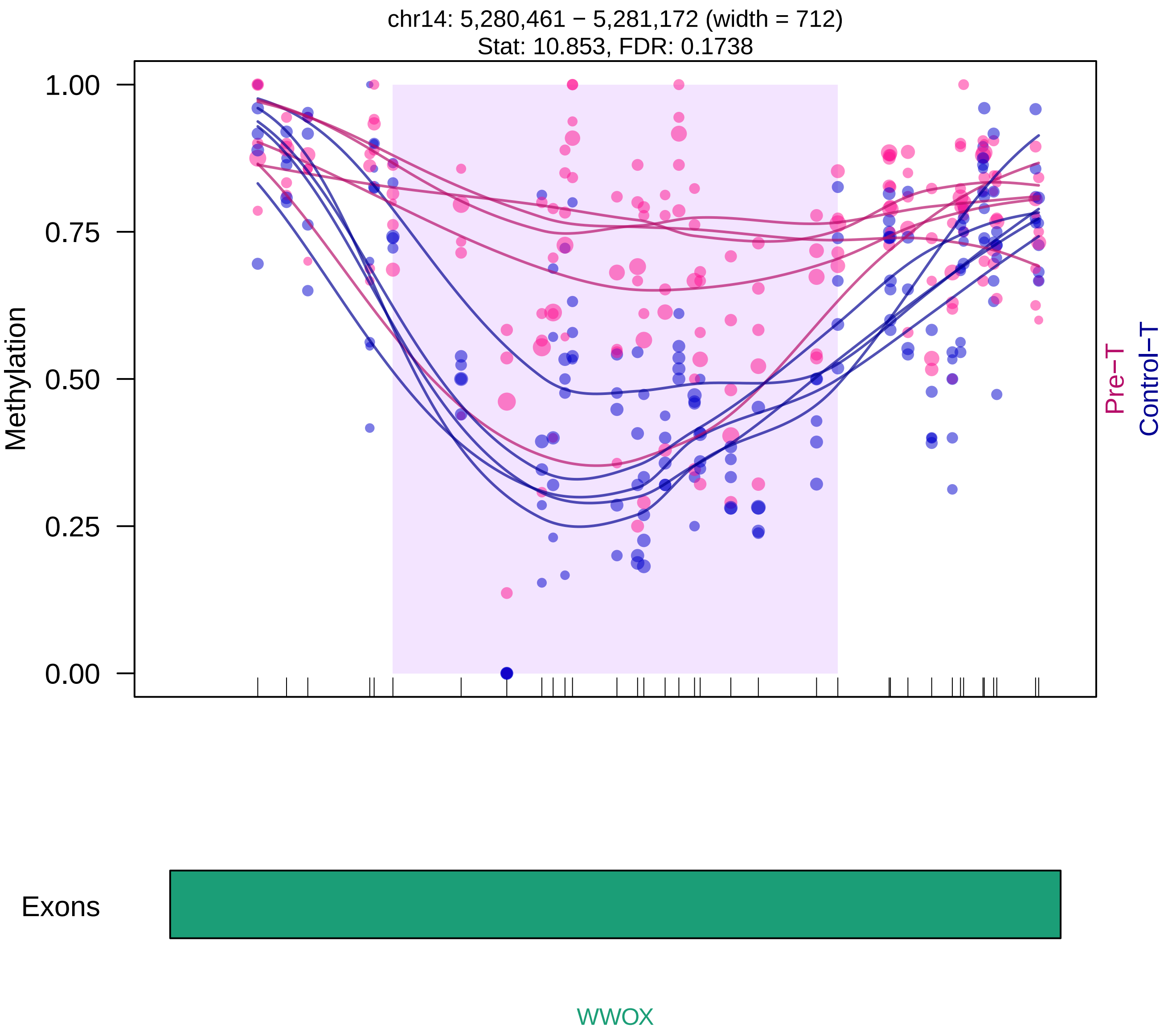

chr14: 5,280,461 − 5,281,172 (width = 712)
Stat: 10.853, FDR: 0.1738
Methylation
1.00
0.75
0.50
0.25
0.00
Pre−T
Control−T
Exons
WWOX

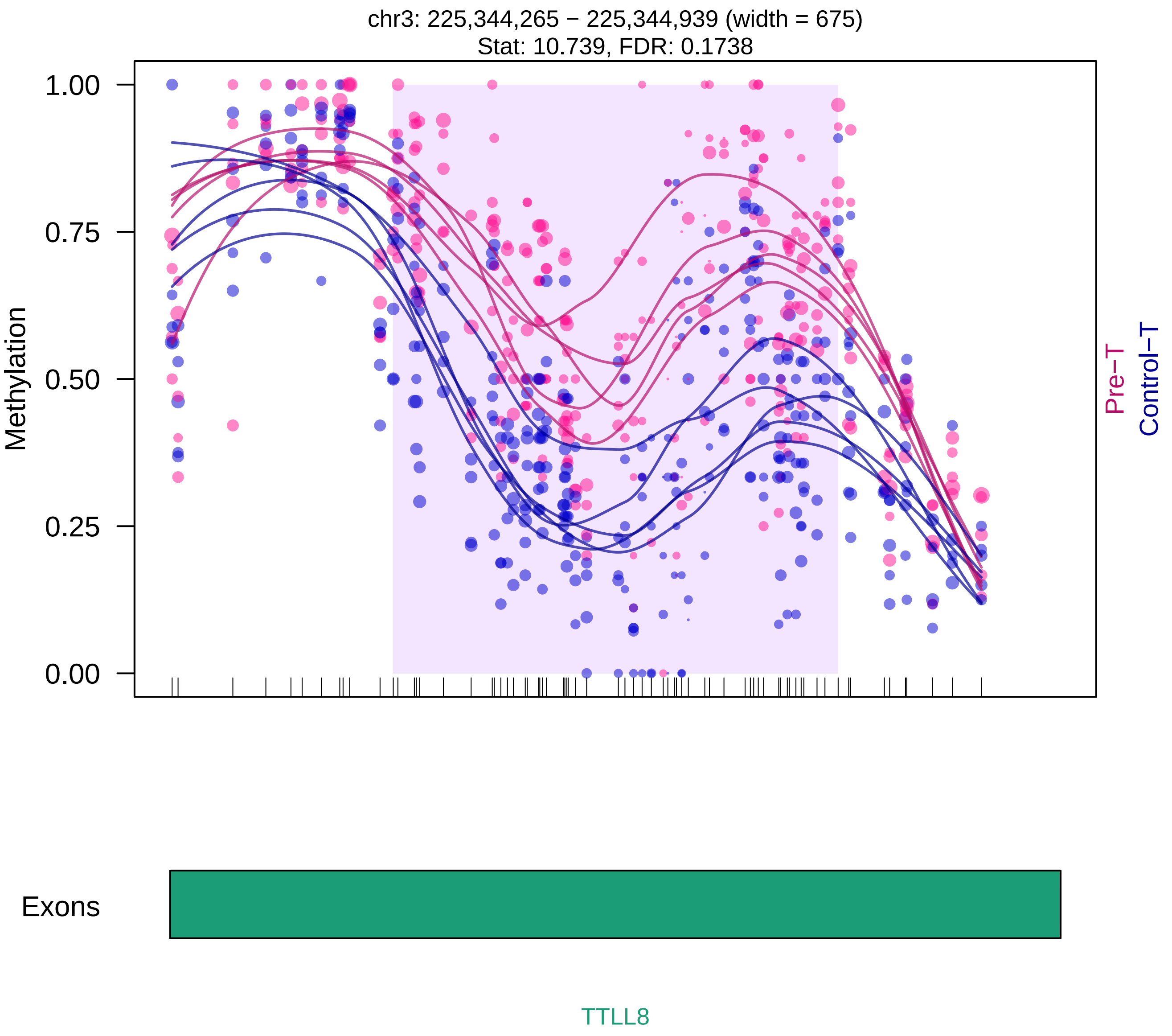

chr3: 225,344,265 − 225,344,939 (width = 675)
Stat: 10.739, FDR: 0.1738
Methylation
1.00
0.75
0.50
0.25
0.00
Pre−T
Control−T
Exons
TTLL8

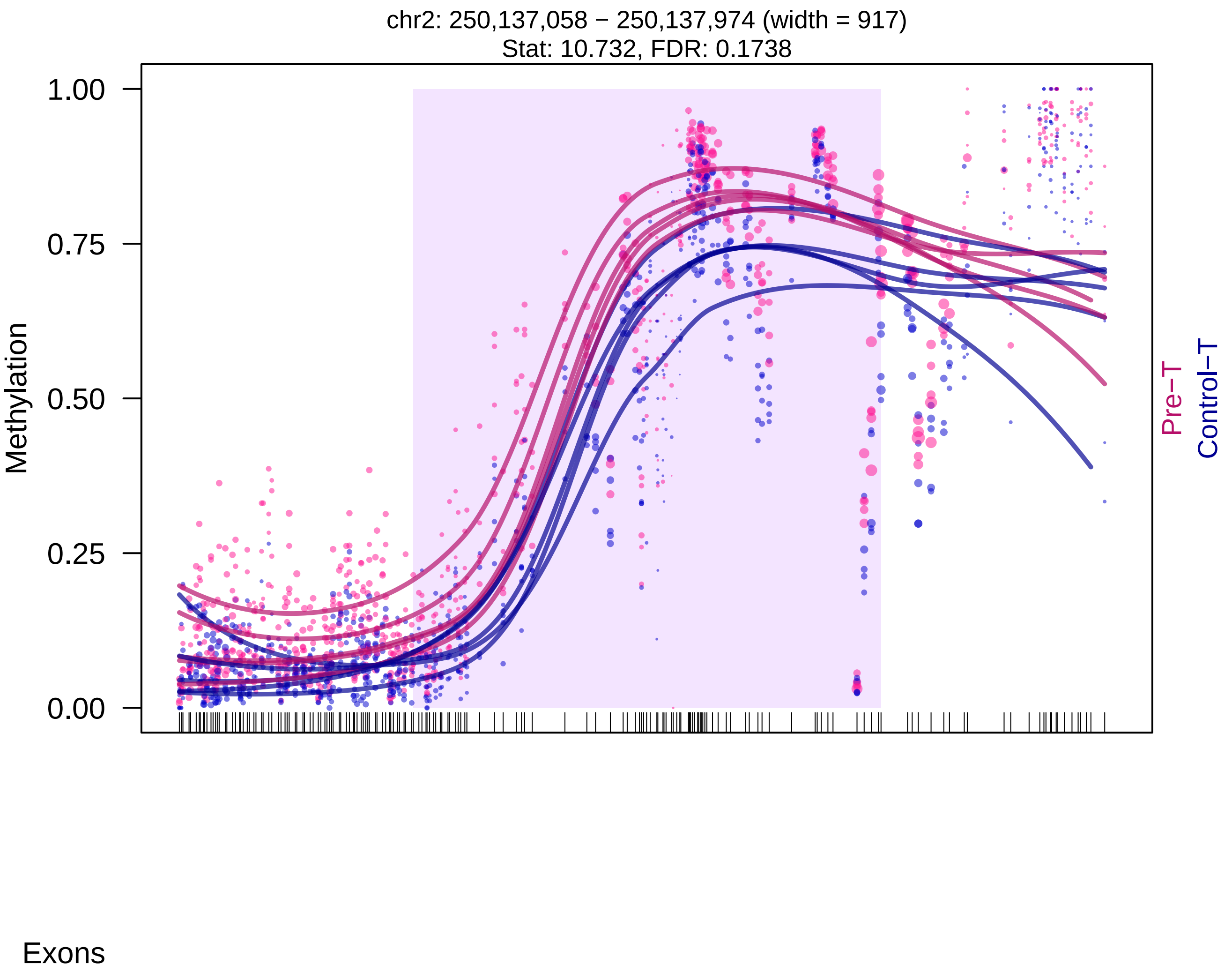

chr2: 250,137,058 − 250,137,974 (width = 917)
Stat: 10.732, FDR: 0.1738
1.00
0.75
0.50
0.25
0.00
Methylation
Pre−T
Control−T
Exons

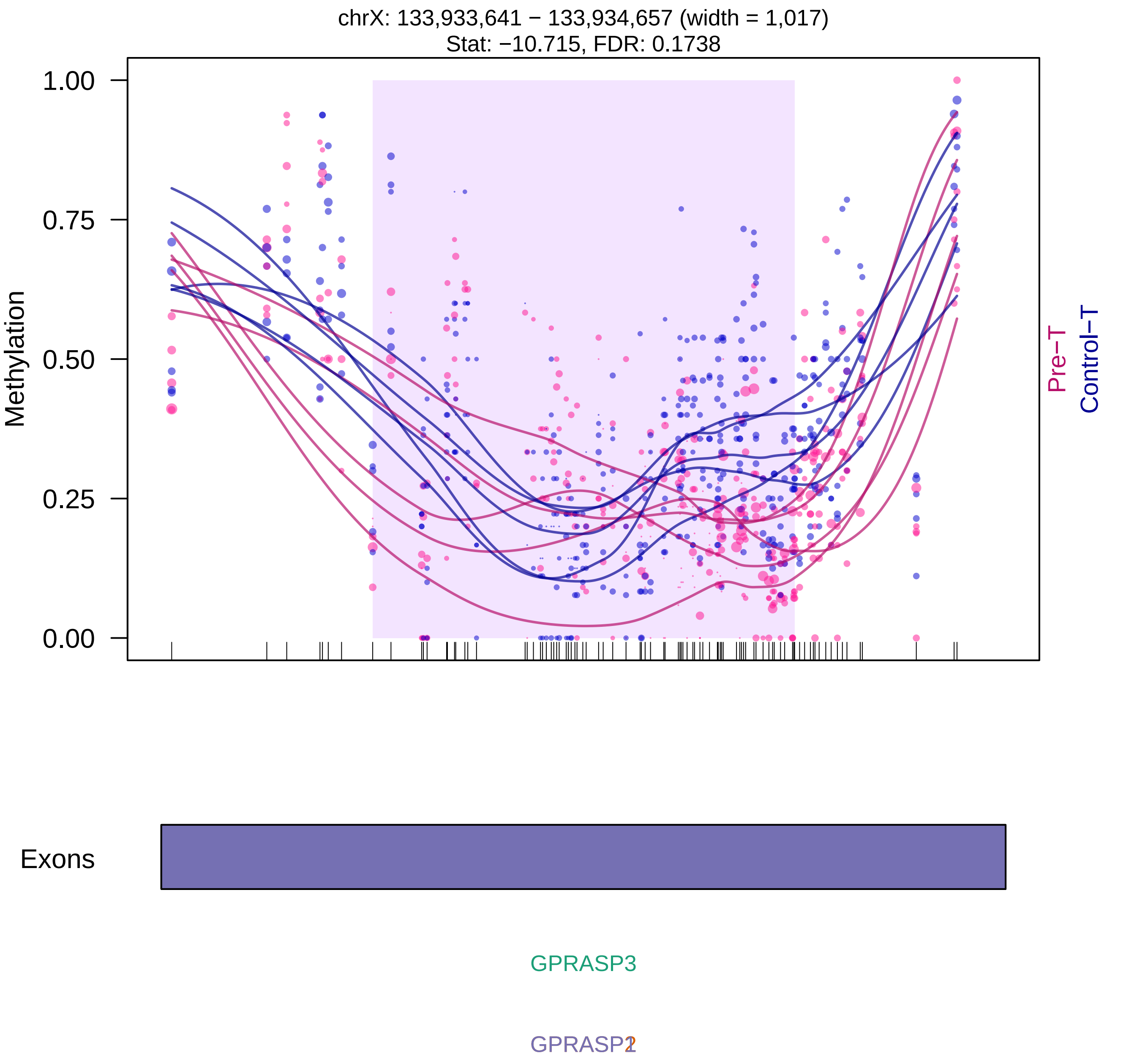

chrX: 133,933,641 − 133,934,657 (width = 1,017)
Stat: −10.715, FDR: 0.1738
1.00
0.75
0.50
0.25
0.00
Methylation
Pre−T
Control−T
Exons
GPRASP3
GPRASP1
GPRASP2

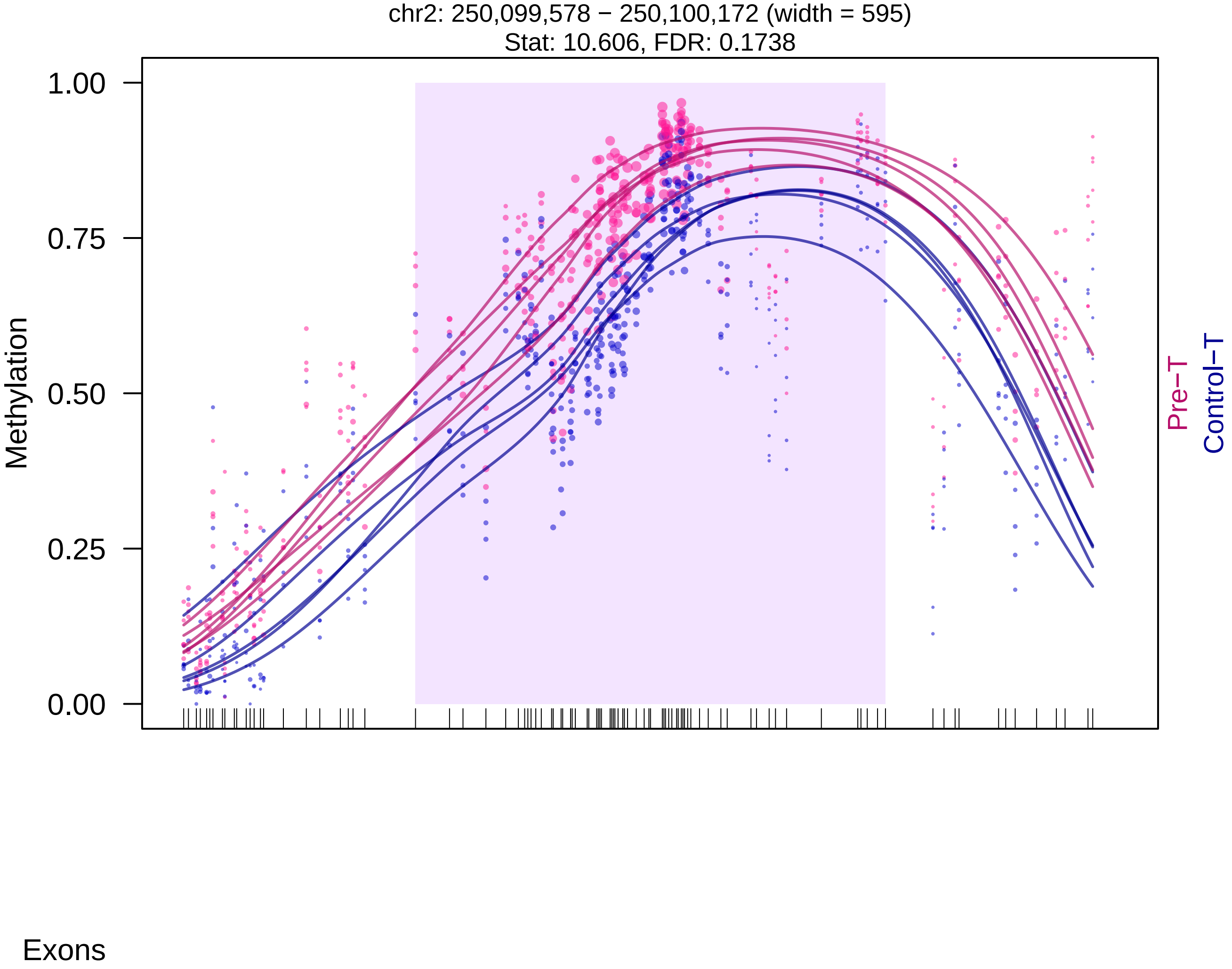

chr2: 250,099,578 − 250,100,172 (width = 595)
Stat: 10.606, FDR: 0.1738
1.00
0.75
0.50
0.25
0.00
Methylation
Pre−T
Control−T
Exons

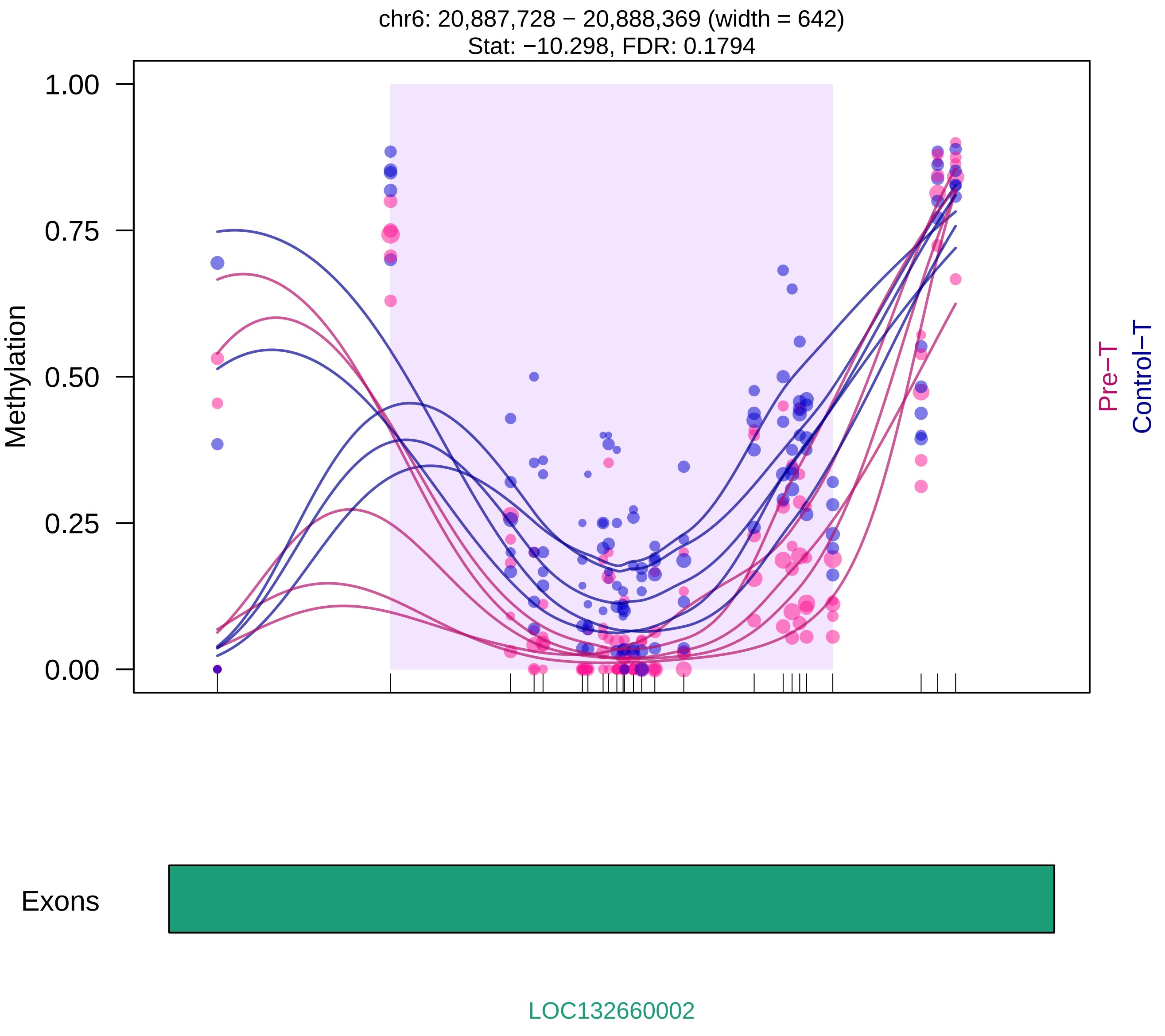
chr6: 20,887,728 − 20,888,369 (width = 642)
Stat: −10.298, FDR: 0.1794
Methylation
1.00
0.75
0.50
0.25
0.00
Pre−T
Control−T
Exons
LOC132660002

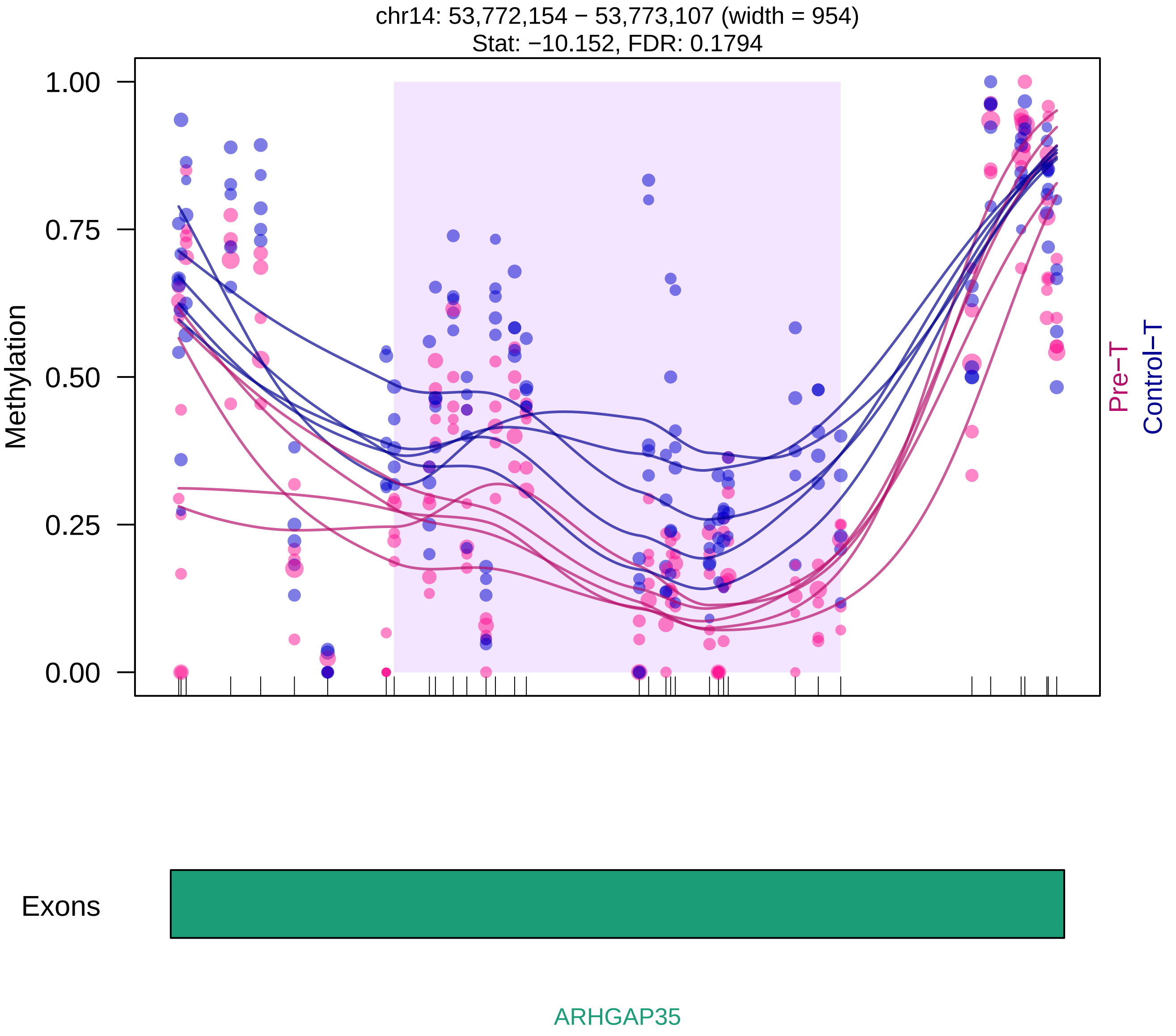

chr14: 53,772,154 − 53,773,107 (width = 954)
Stat: −10.152, FDR: 0.1794
Methylation
1.00
0.75
0.50
0.25
0.00
Pre−T
Control−T
Exons
ARHGAP35

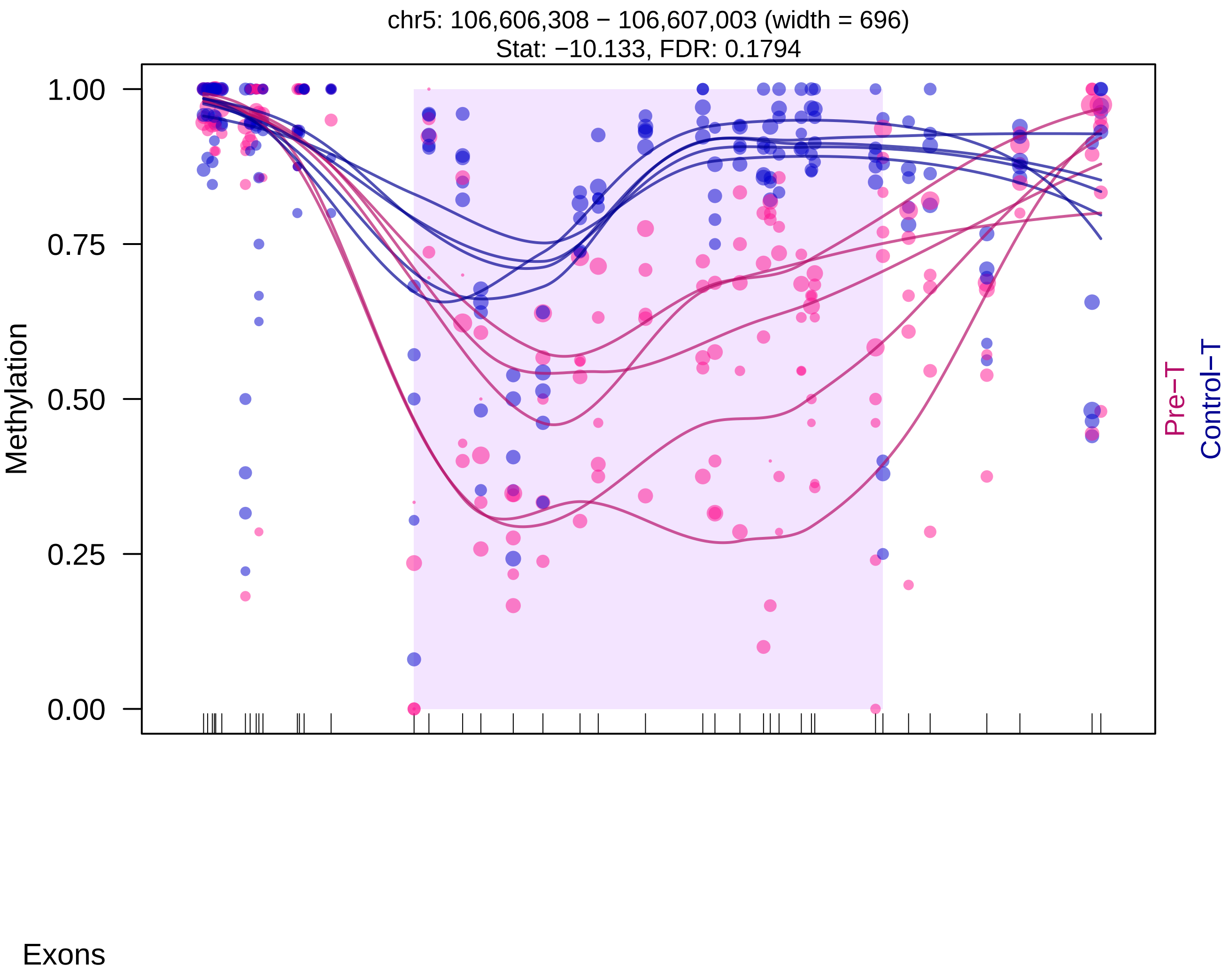
chr5: 106,606,308 − 106,607,003 (width = 696)
Stat: −10.133, FDR: 0.1794
1.00
0.75
0.50
0.25
0.00
Methylation
Pre−T
Control−T
Exons

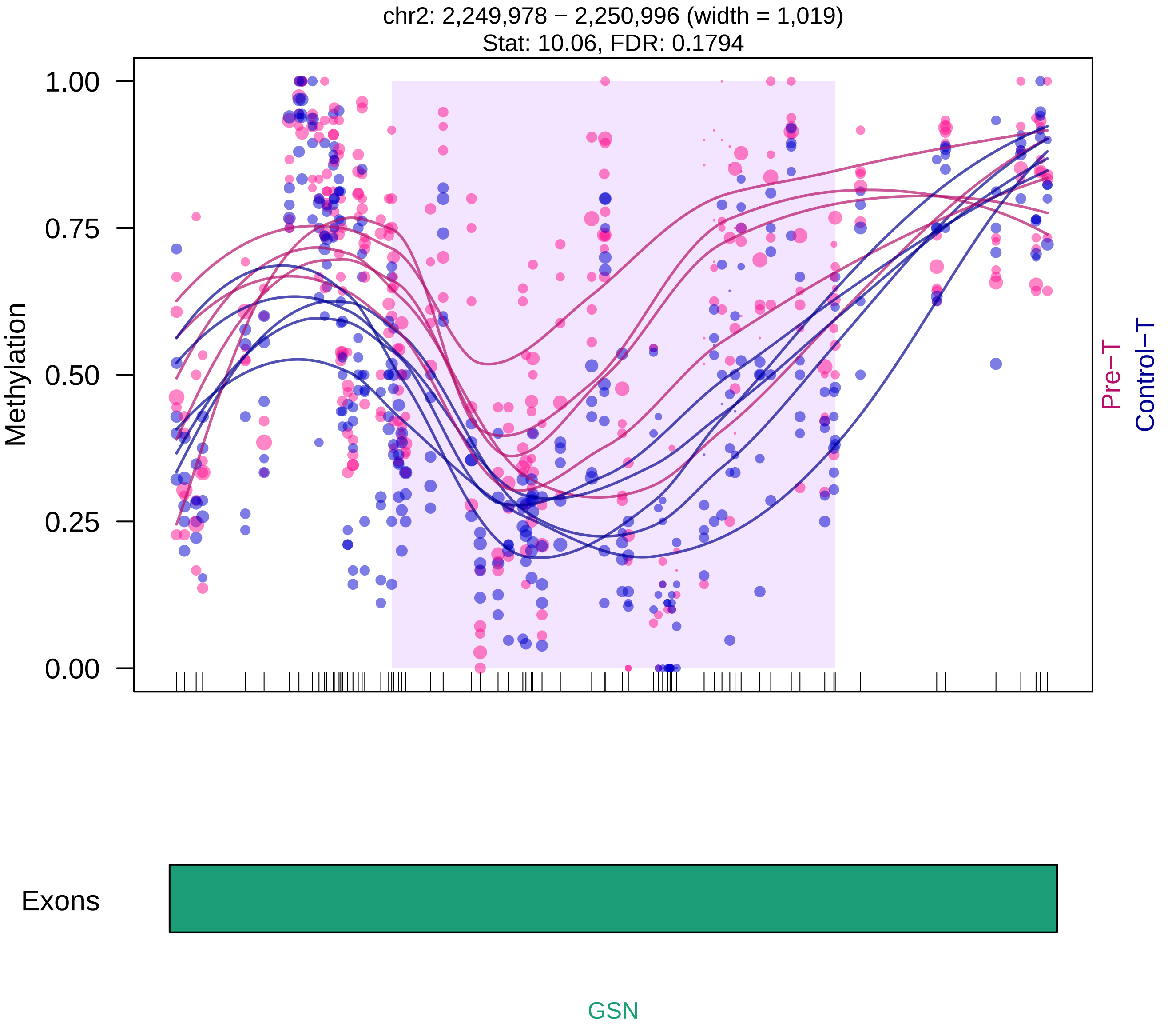
chr2: 2,249,978 − 2,250,996 (width = 1,019)
Stat: 10.06, FDR: 0.1794
Methylation
1.00
0.75
0.50
0.25
0.00
Pre−T
Control−T
Exons
GSN

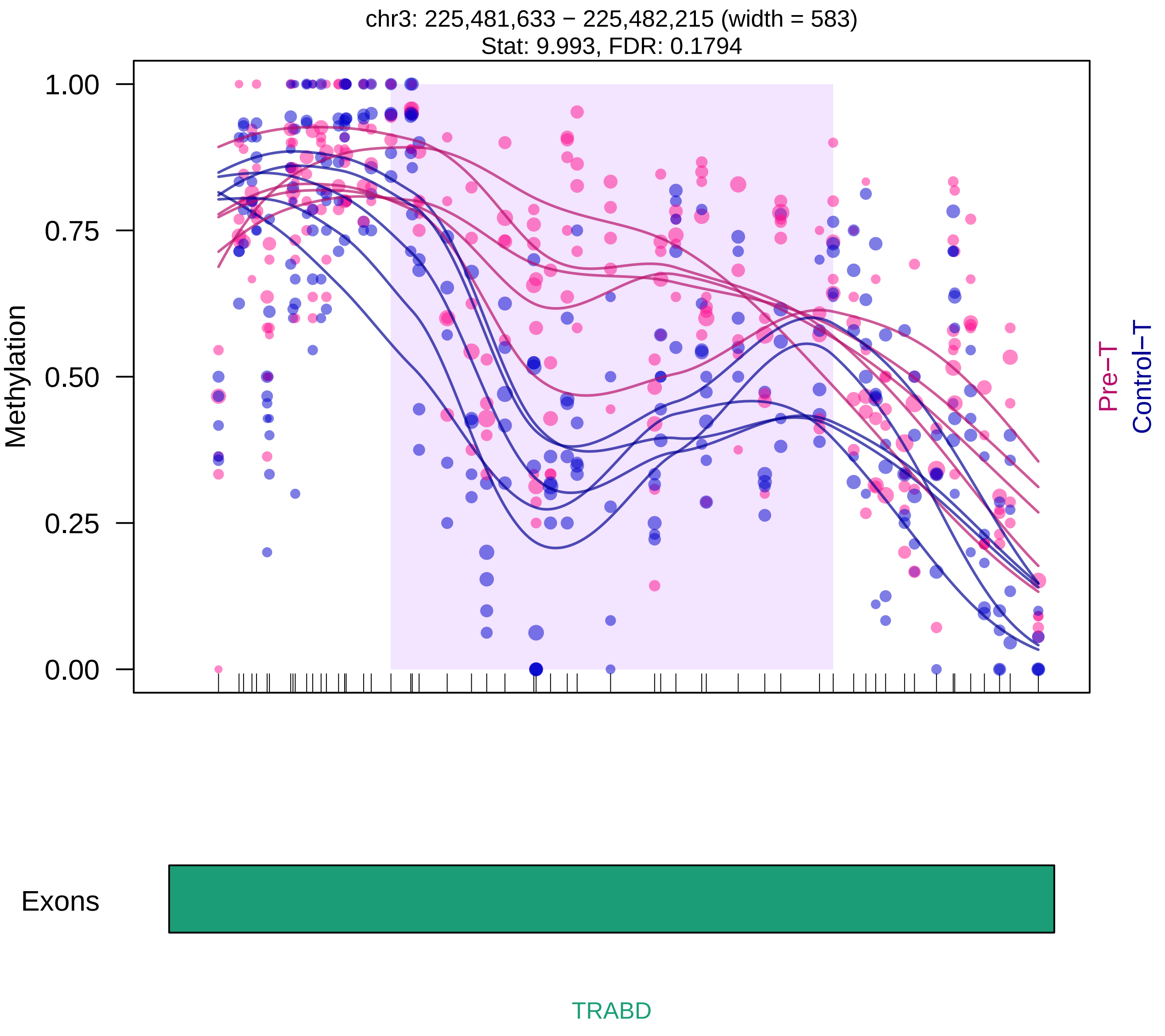

chr3: 225,481,633 − 225,482,215 (width = 583)
Stat: 9.993, FDR: 0.1794
Methylation
1.00
0.75
0.50
0.25
0.00
Pre−T
Control−T
Exons
TRABD

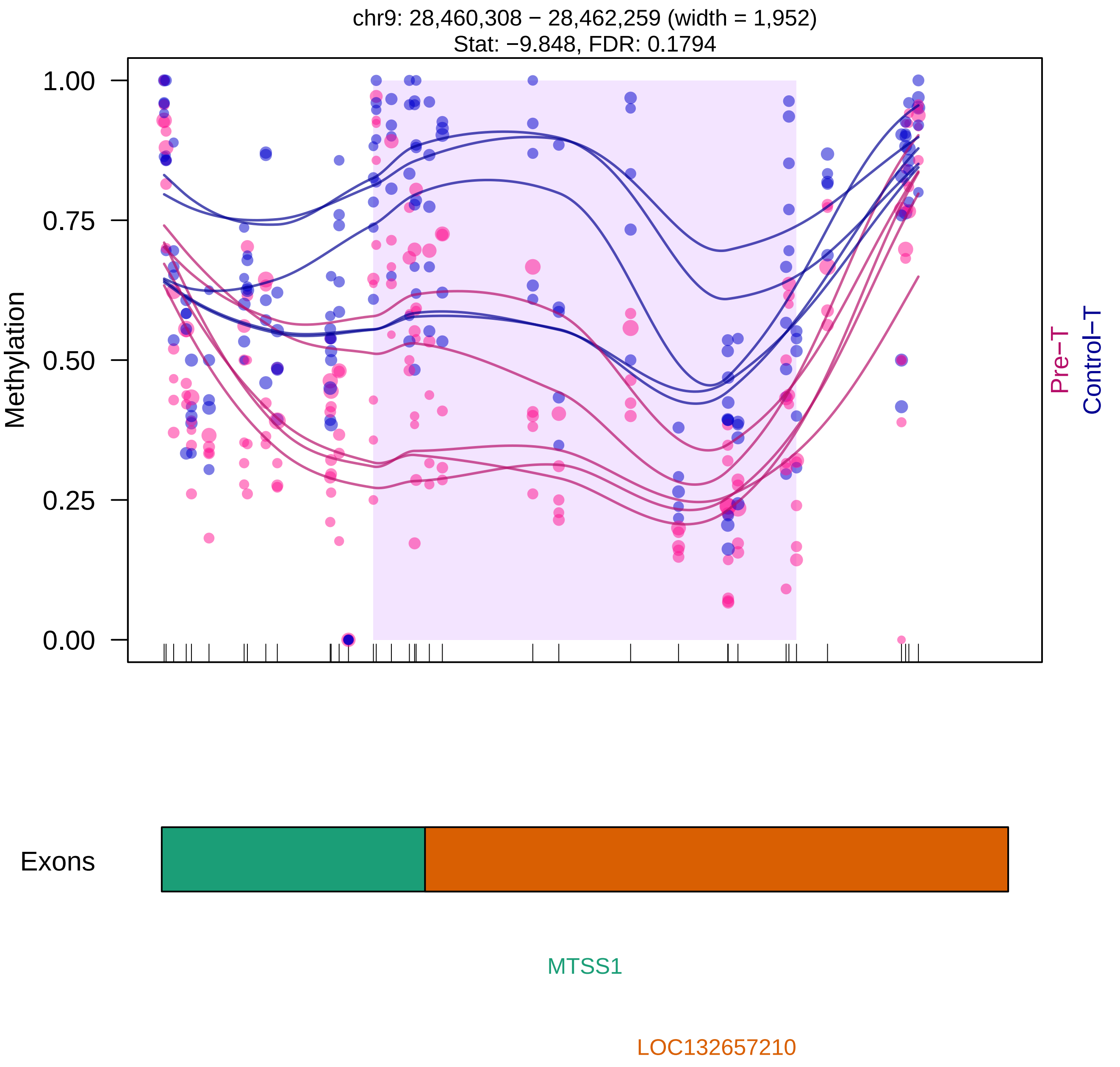
chr9: 28,460,308 − 28,462,259 (width = 1,952)
Stat: −9.848, FDR: 0.1794
Methylation
1.00
0.75
0.50
0.25
0.00
Pre−T
Control−T
Exons
MTSS1
LOC132657210

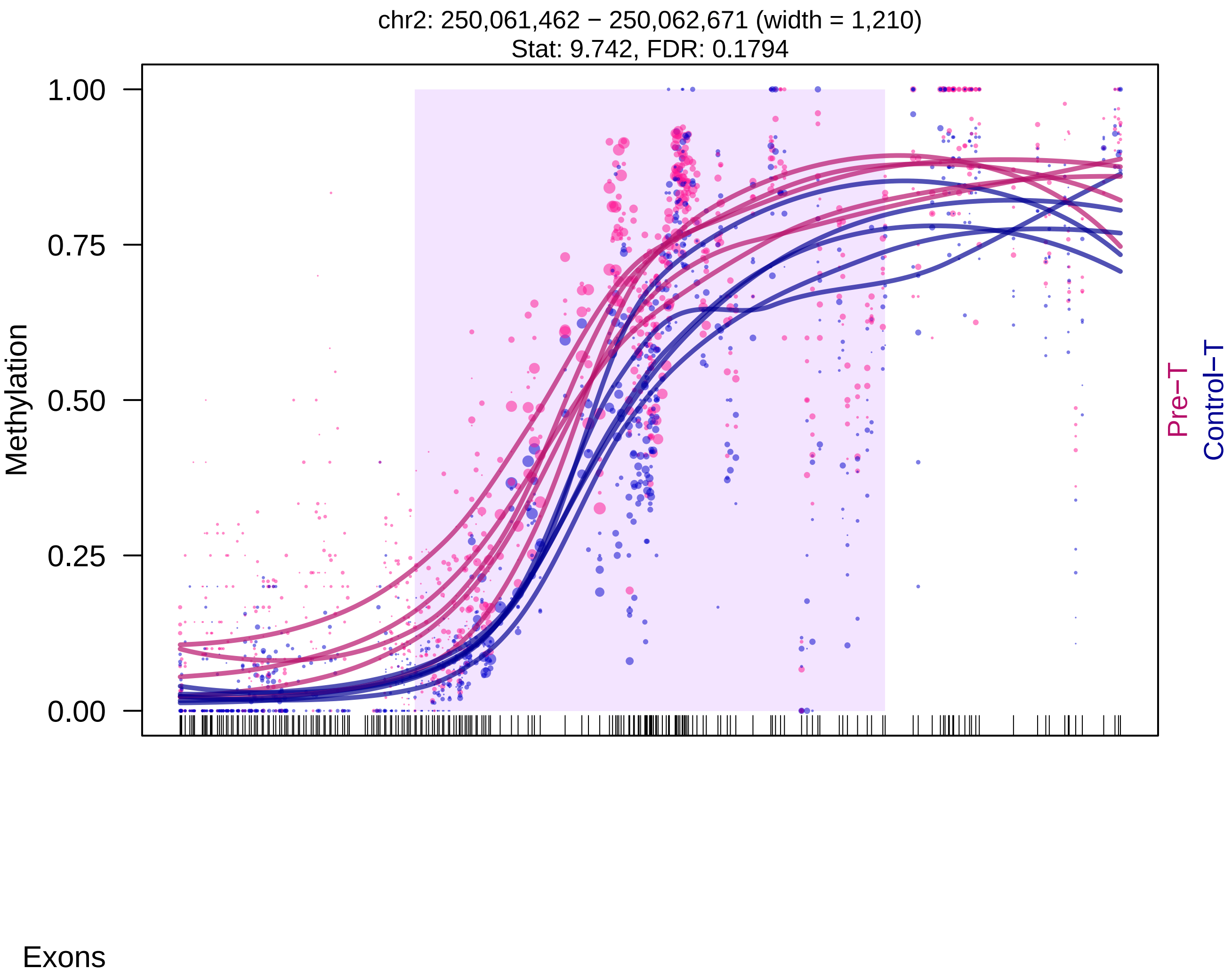
chr2: 250,061,462 − 250,062,671 (width = 1,210)
Stat: 9.742, FDR: 0.1794
Methylation
1.00
0.75
0.50
0.25
0.00
Pre−T
Control−T
Exons

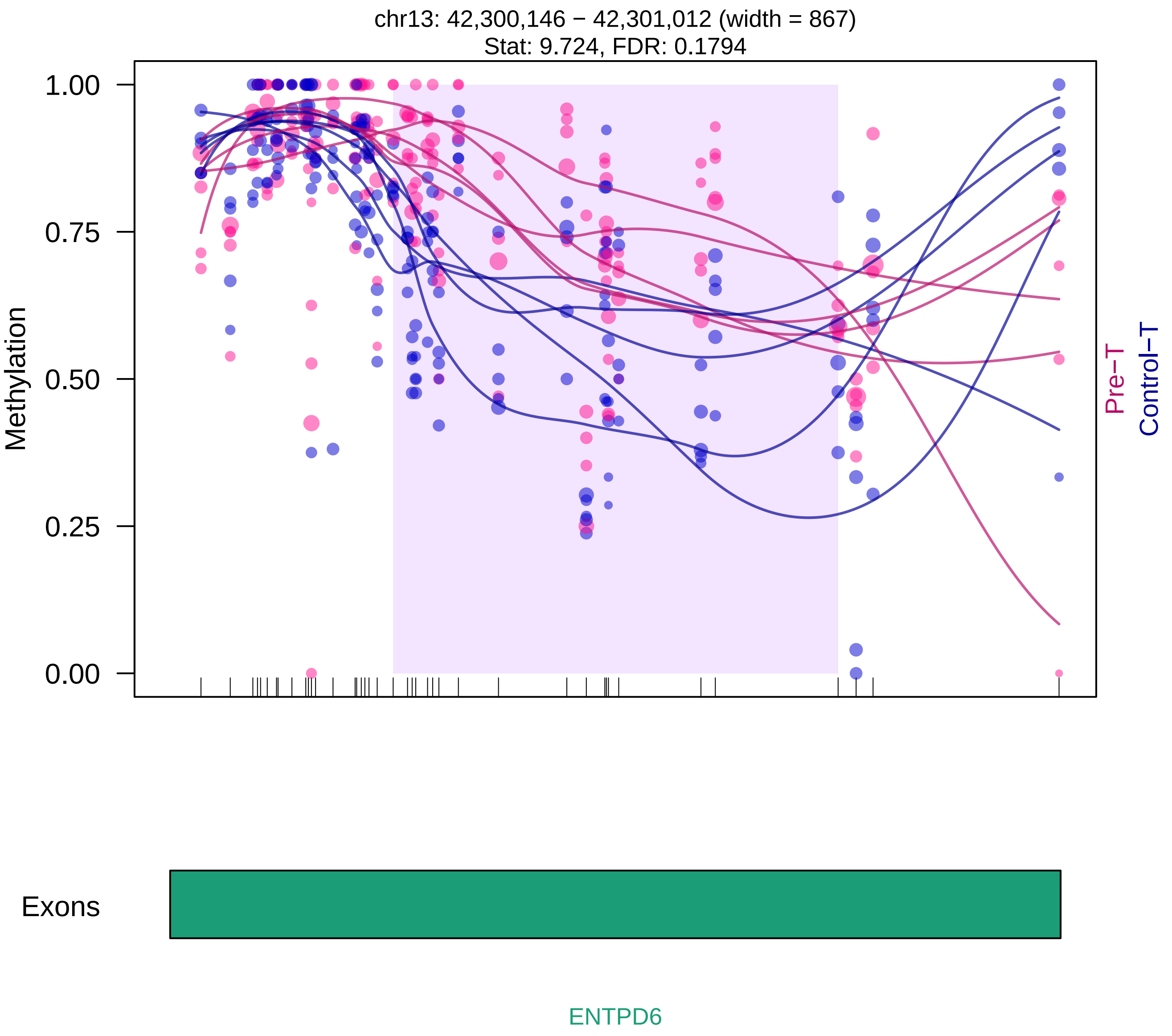

chr13: 42,300,146 − 42,301,012 (width = 867)
Stat: 9.724, FDR: 0.1794
1.00
0.75
0.50
0.25
0.00
Methylation
Pre−T
Control−T
Exons
ENTPD6

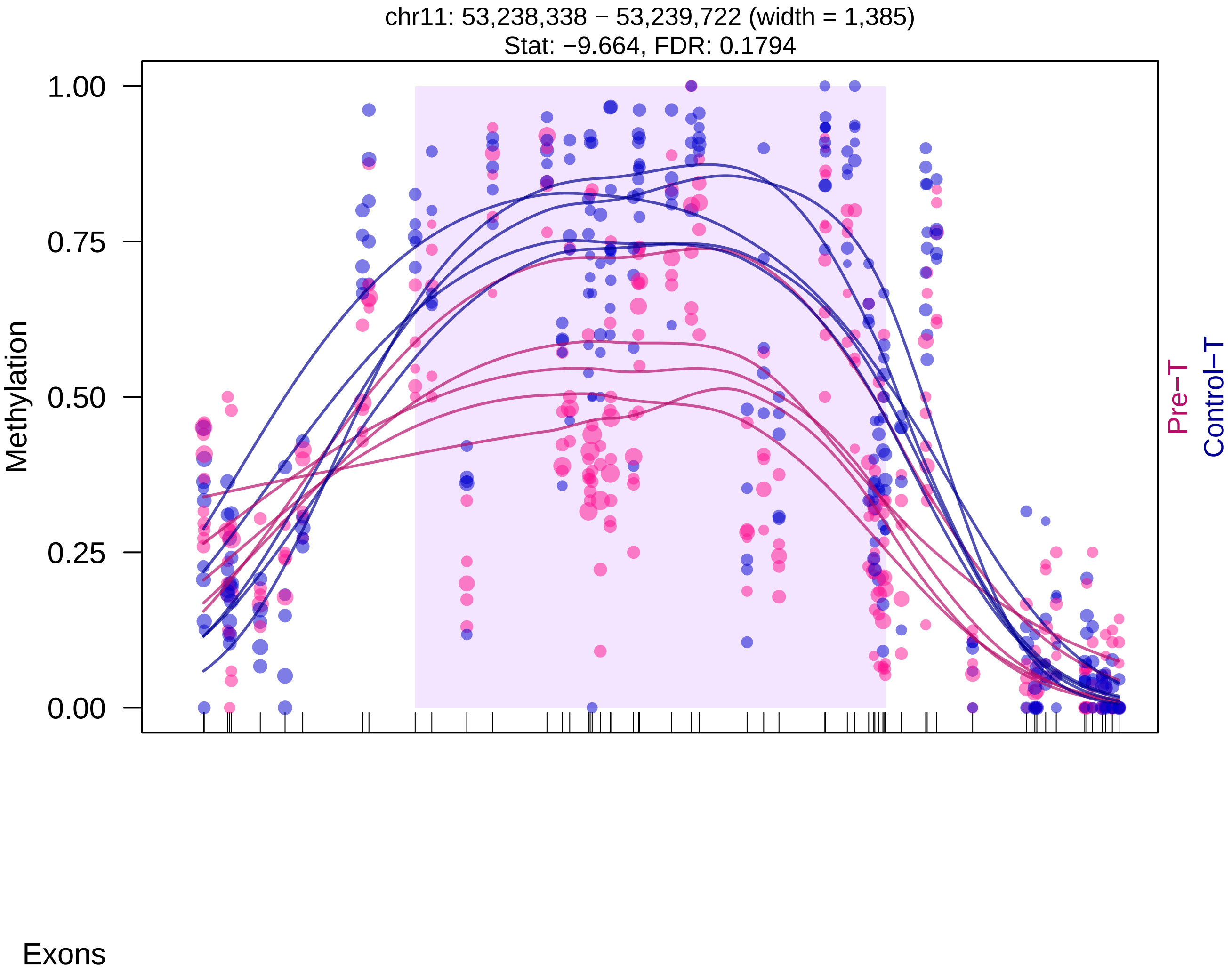
chr11: 53,238,338 − 53,239,722 (width = 1,385)
Stat: −9.664, FDR: 0.1794
Methylation
1.00
0.75
0.50
0.25
0.00
Pre−T
Control−T
Exons

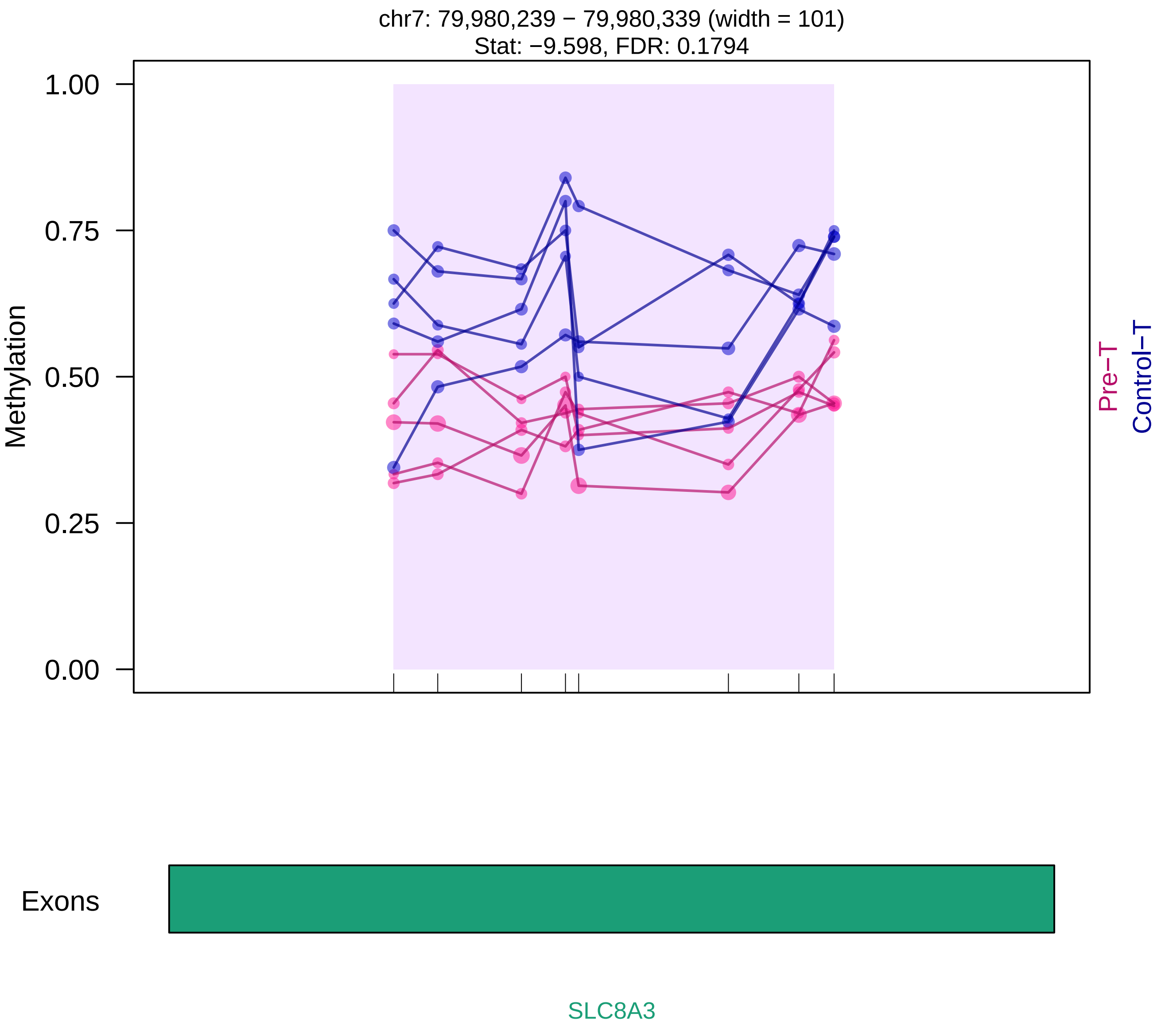

chr7: 79,980,239 − 79,980,339 (width = 101)
Stat: −9.598, FDR: 0.1794
Methylation
1.00
0.75
0.50
0.25
0.00
Pre−T
Control−T
Exons
SLC8A3

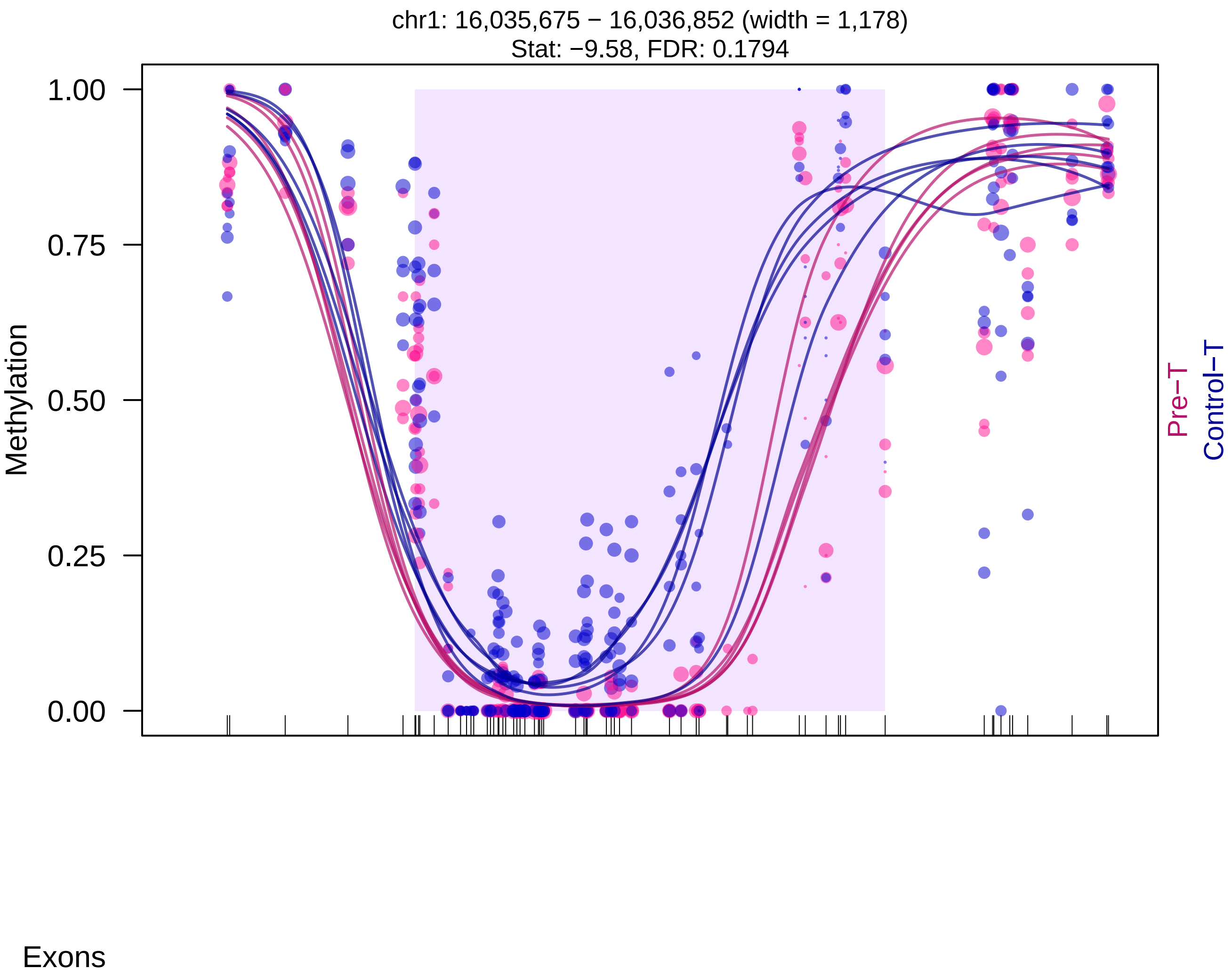

chr1: 16,035,675 − 16,036,852 (width = 1,178)
Stat: −9.58, FDR: 0.1794
1.00
0.75
0.50
0.25
0.00
Methylation
Pre−T
Control−T
Exons

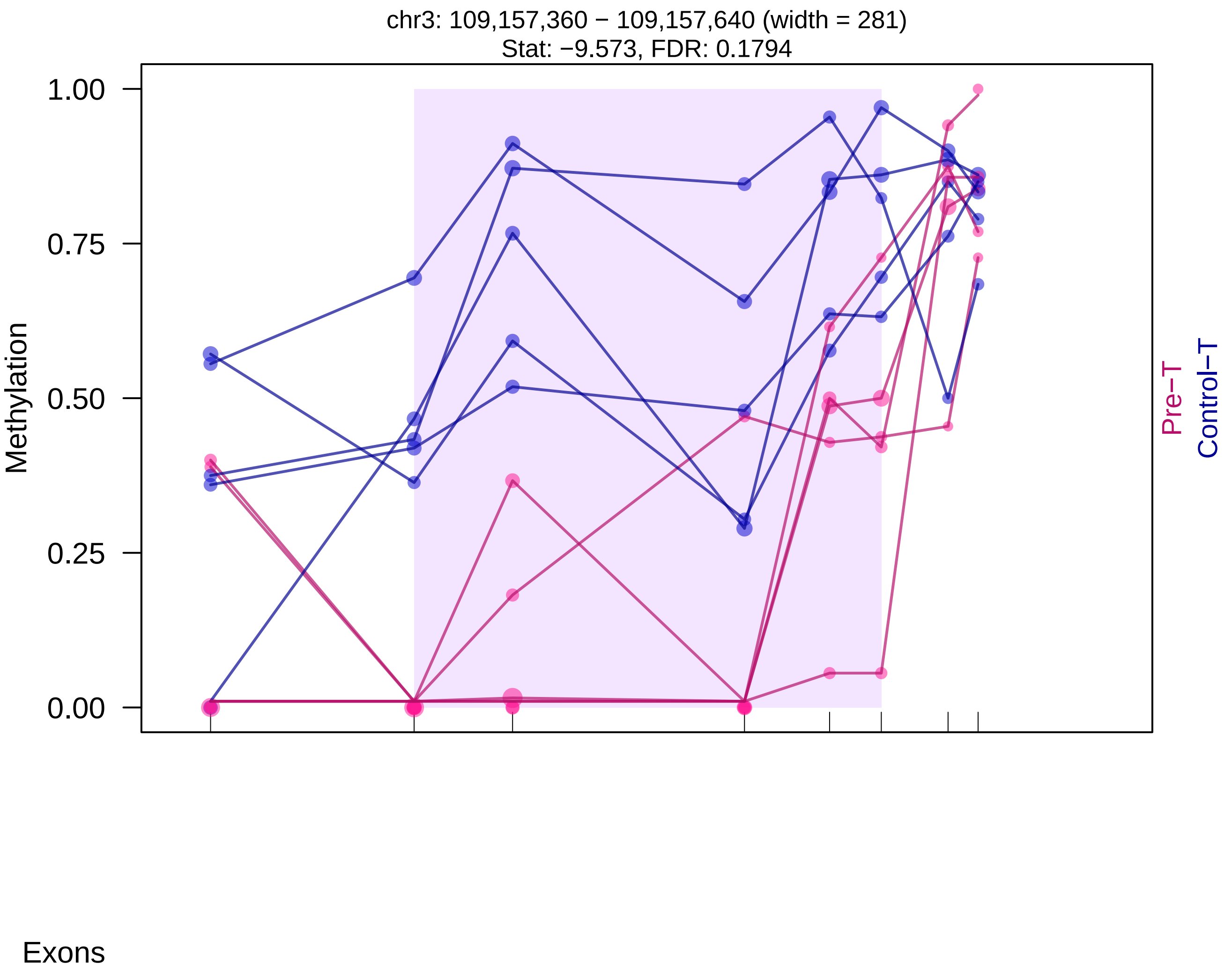
chr3: 109,157,360 − 109,157,640 (width = 281)
Stat: −9.573, FDR: 0.1794
1.00
0.75
0.50
0.25
0.00
Methylation
Pre−T
Control−T
Exons

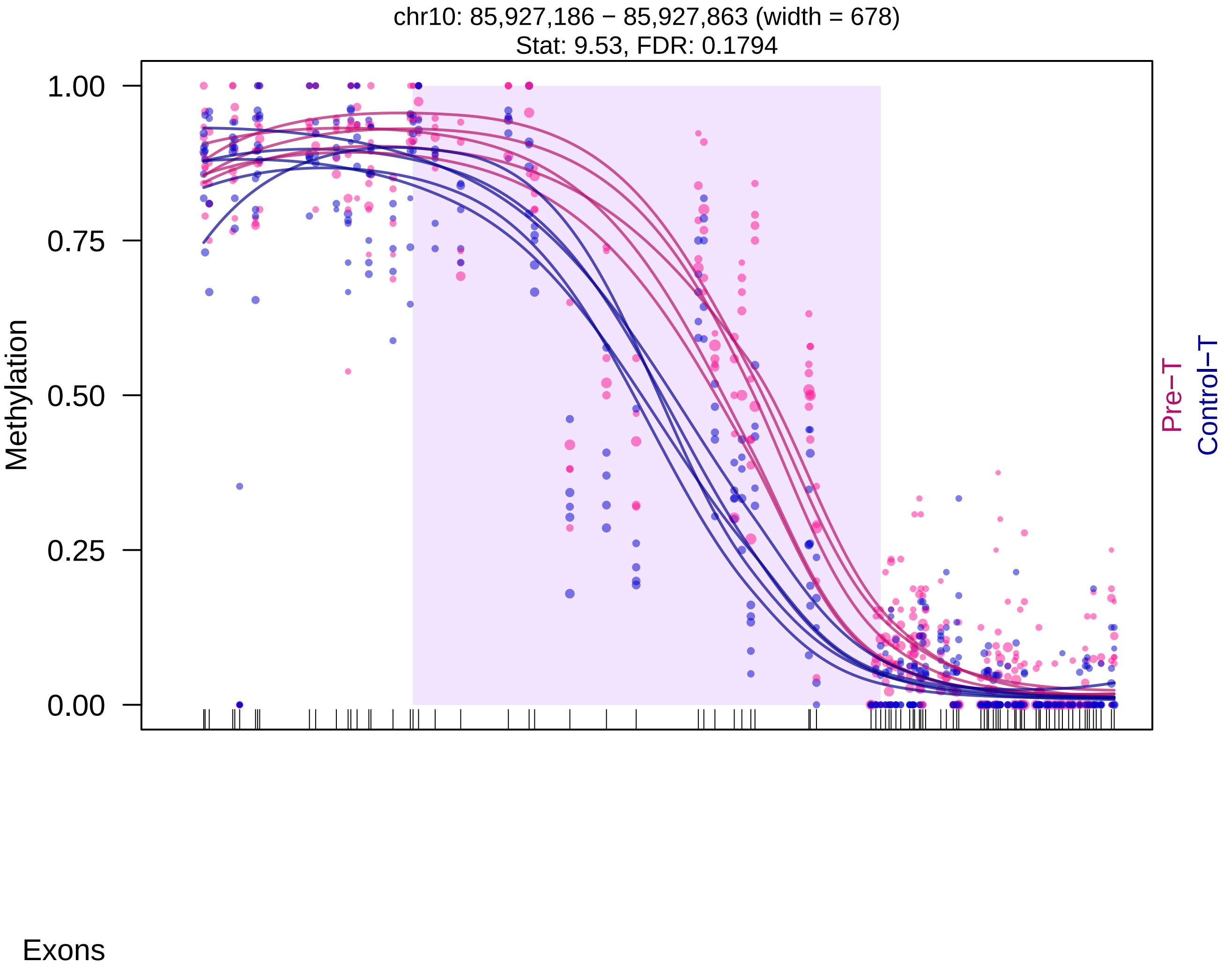
chr10: 85,927,186 − 85,927,863 (width = 678)
Stat: 9.53, FDR: 0.1794
Methylation
1.00
0.75
0.50
0.25
0.00
Pre−T
Control−T
Exons

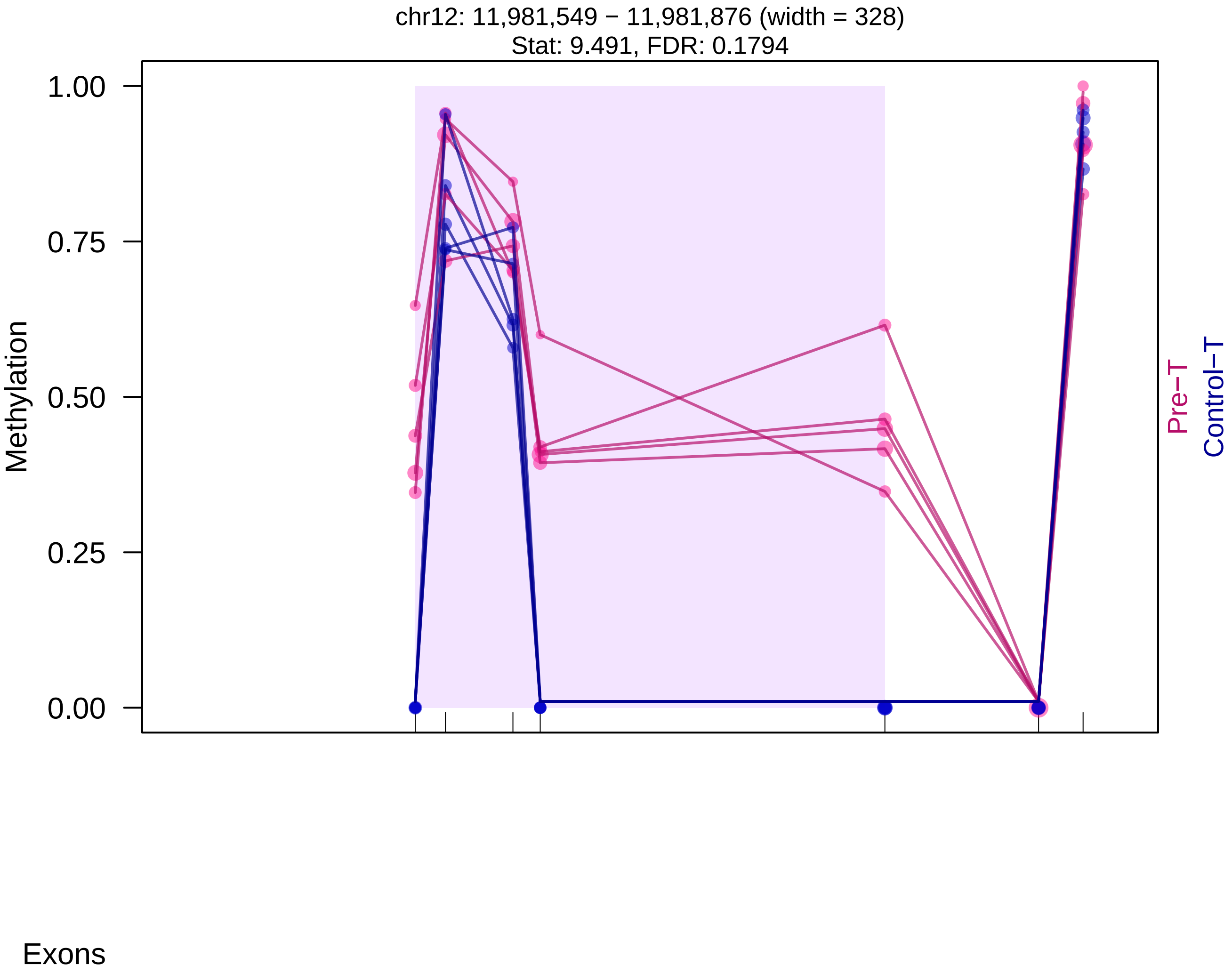

chr12: 11,981,549 − 11,981,876 (width = 328)
Stat: 9.491, FDR: 0.1794
Methylation
1.00
0.75
0.50
0.25
0.00
Pre−T
Control−T
Exons

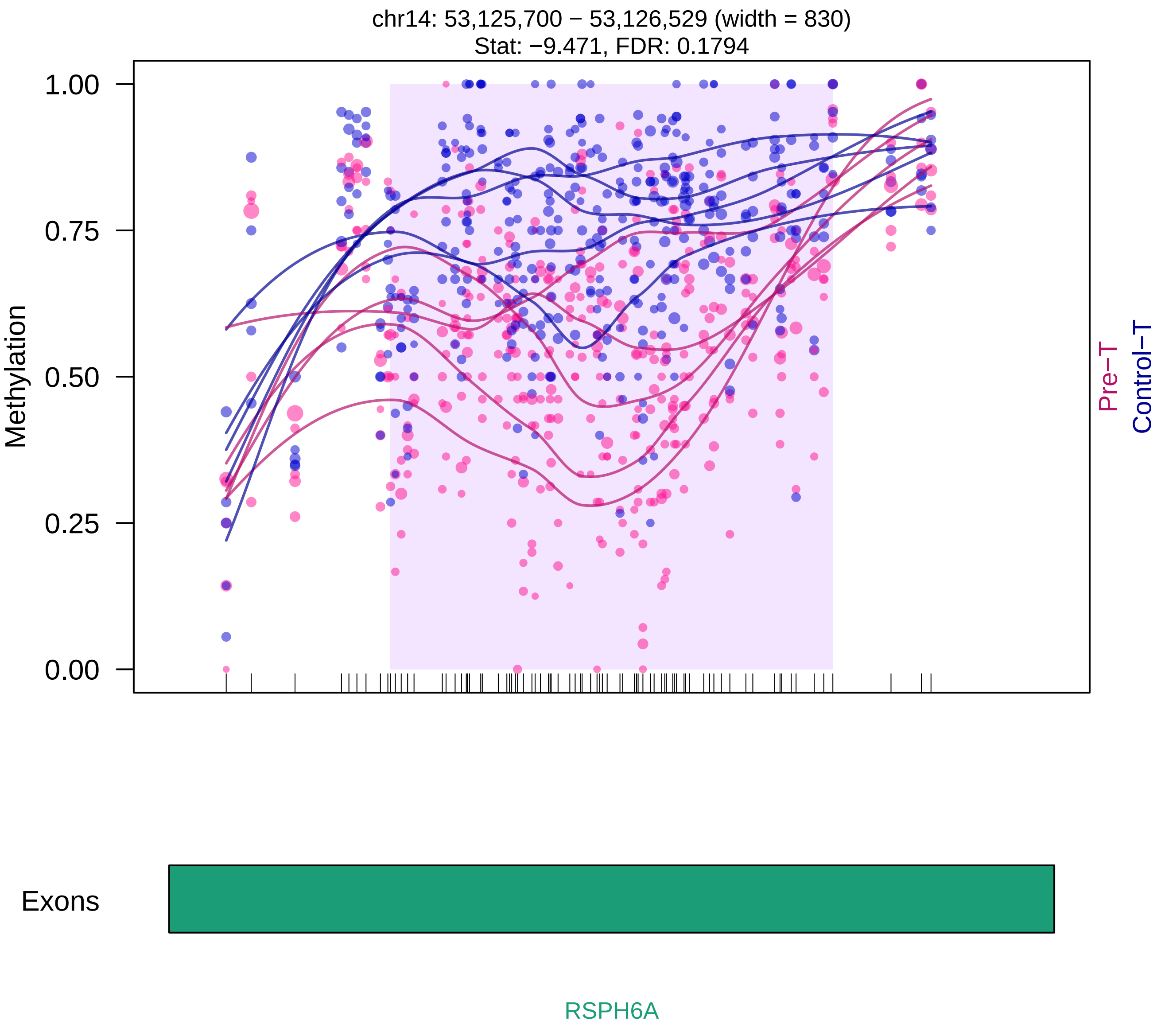
chr14: 53,125,700 − 53,126,529 (width = 830)
Stat: −9.471, FDR: 0.1794
Methylation
1.00
0.75
0.50
0.25
0.00
Pre−T
Control−T
Exons
RSPH6A

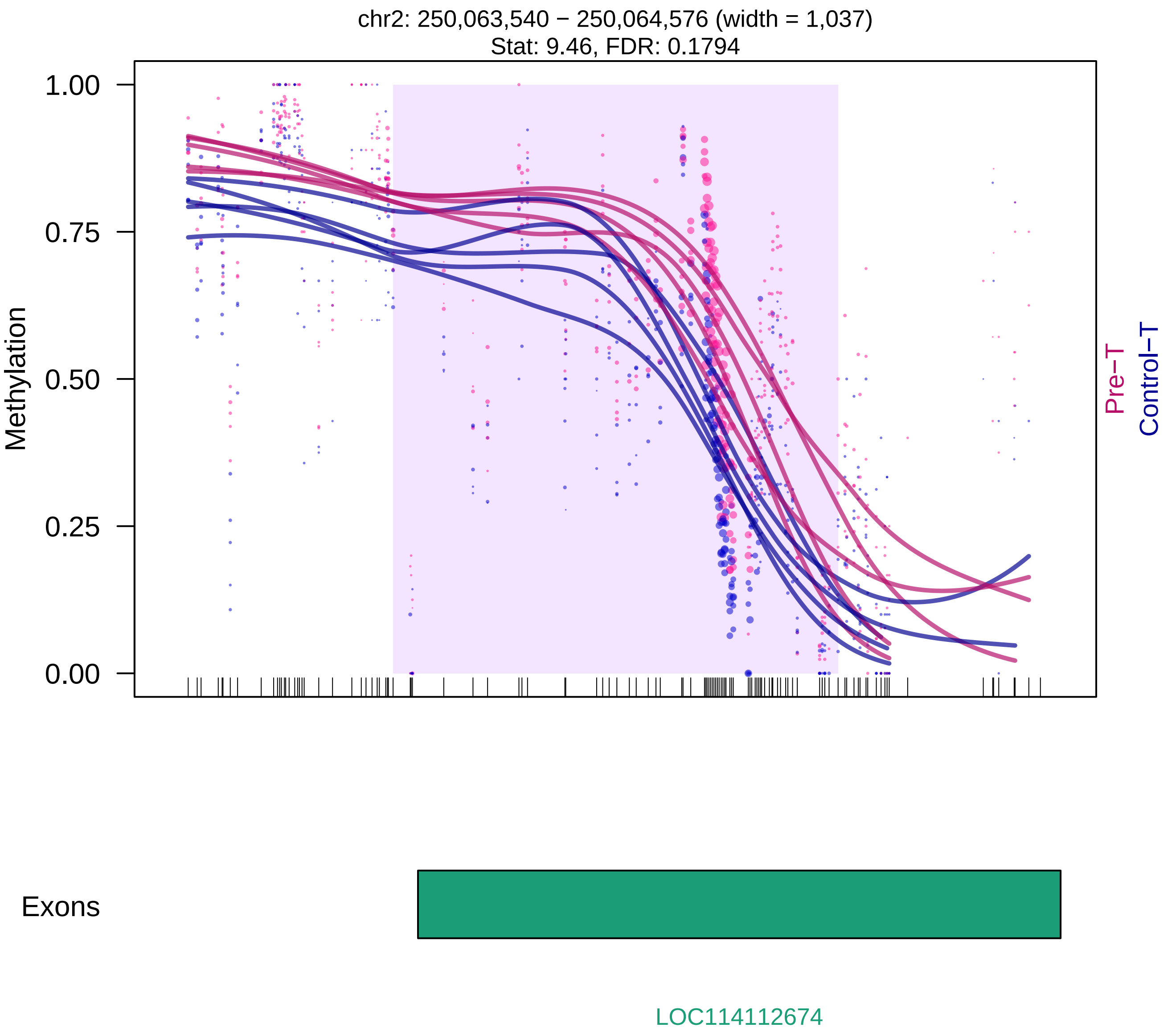
chr2: 250,063,540 − 250,064,576 (width = 1,037)
Stat: 9.46, FDR: 0.1794
Methylation
1.00
0.75
0.50
0.25
0.00
Pre−T
Control−T
Exons
LOC114112674

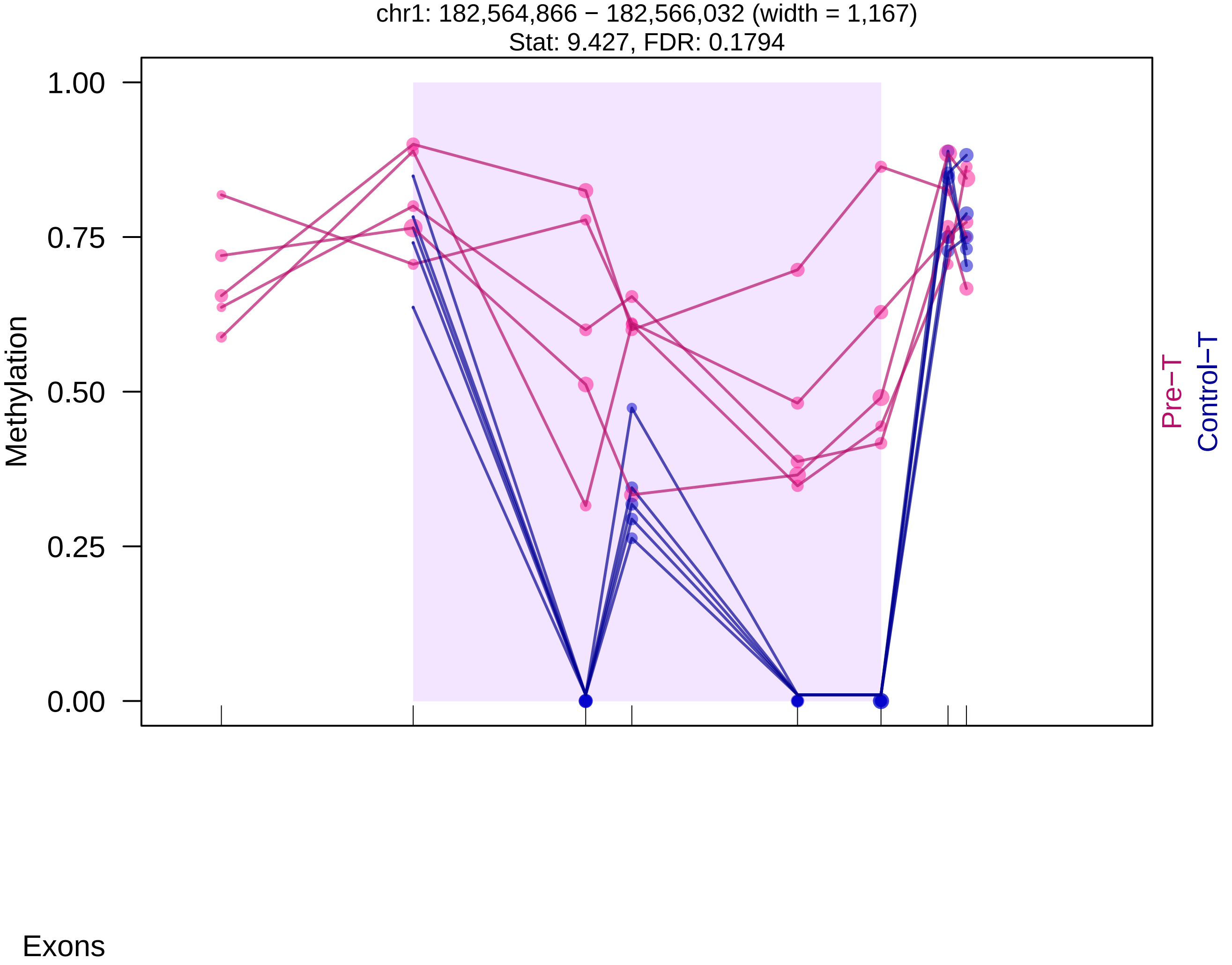

chr1: 182,564,866 − 182,566,032 (width = 1,167)
Stat: 9.427, FDR: 0.1794
1.00
0.75
0.50
0.25
0.00
Methylation
Pre−T
Control−T
Exons

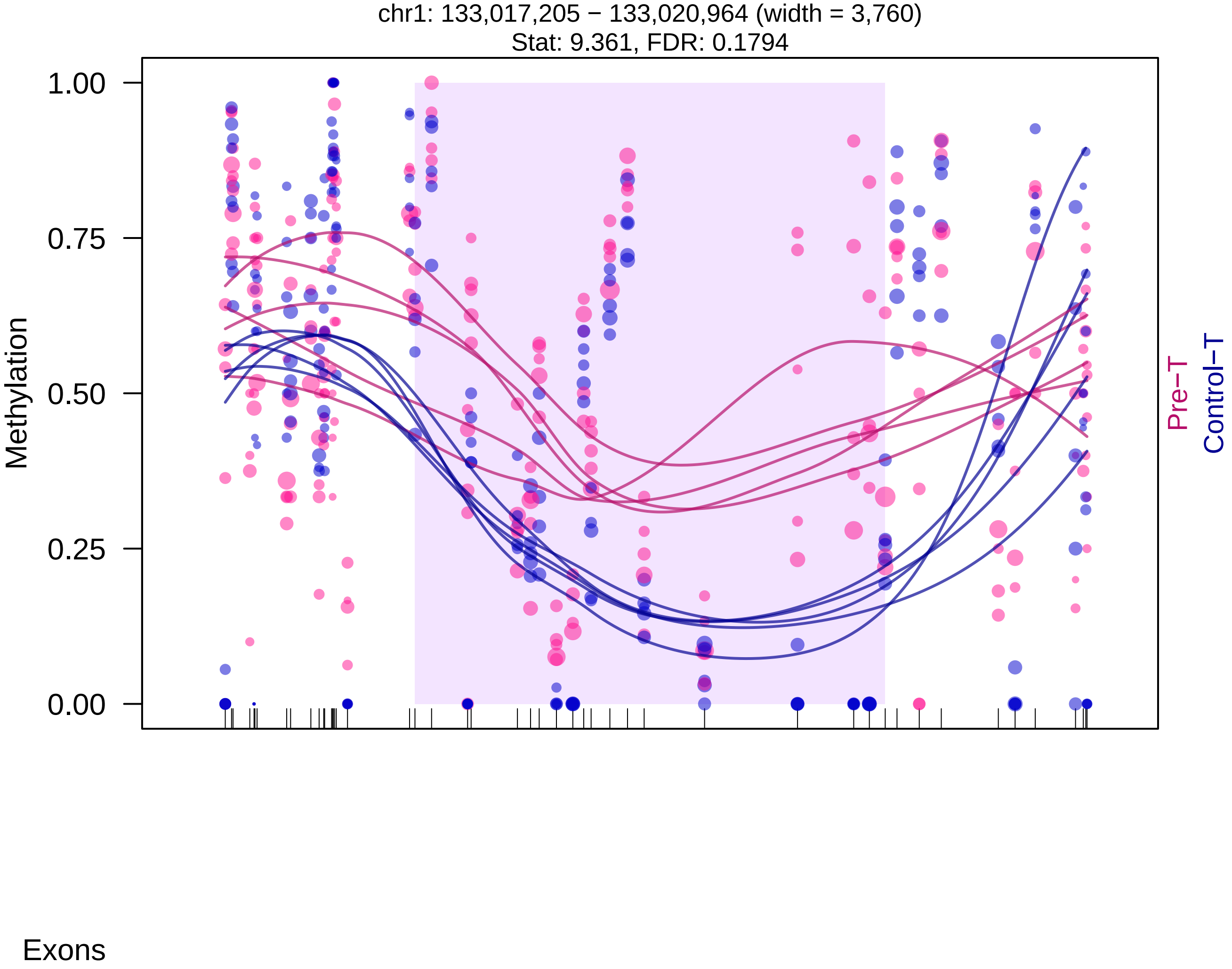

chr1: 133,017,205 − 133,020,964 (width = 3,760)
Stat: 9.361, FDR: 0.1794
Methylation
1.00
0.75
0.50
0.25
0.00
Pre−T
Control−T
Exons

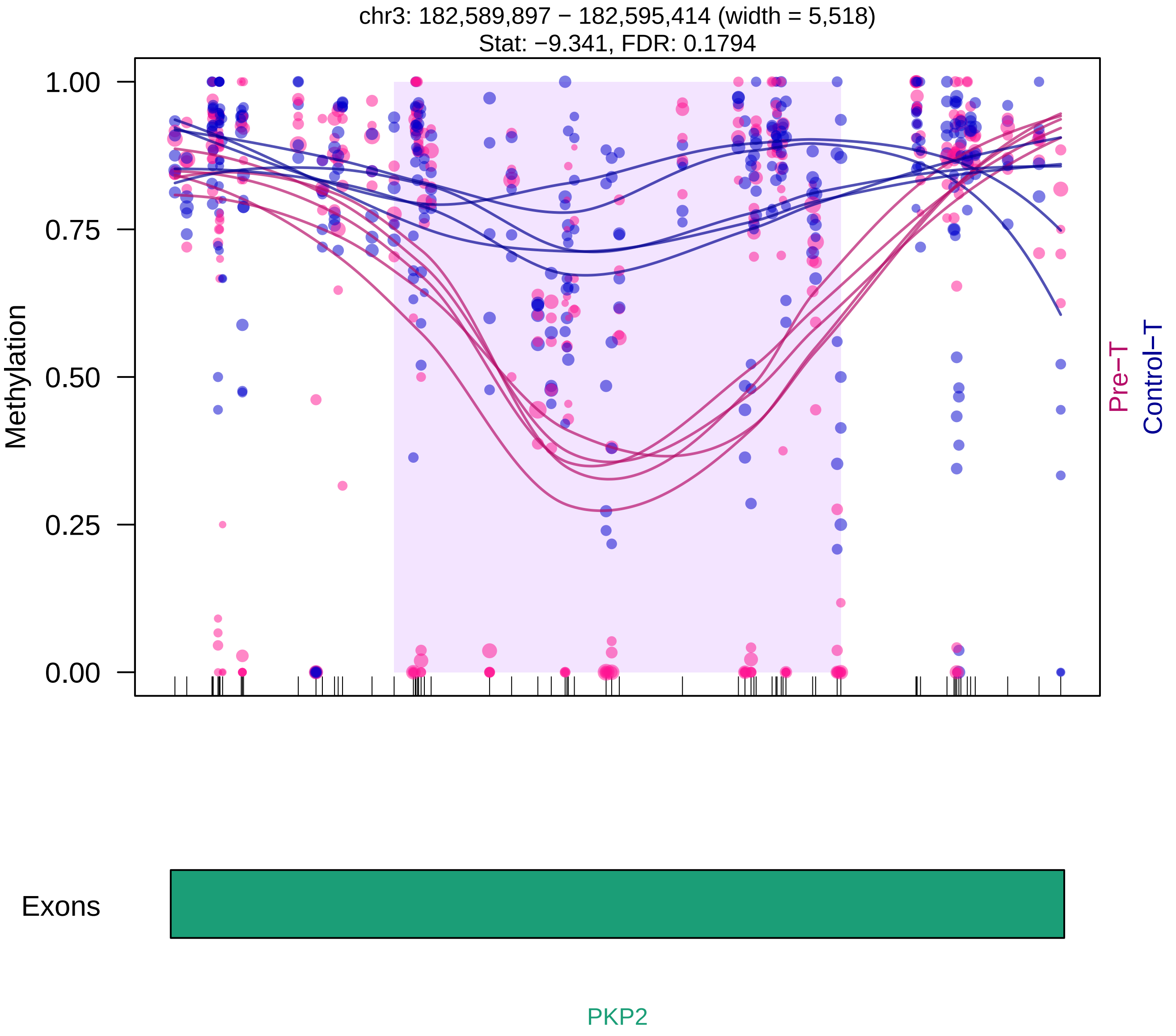
chr3: 182,589,897 − 182,595,414 (width = 5,518)
Stat: −9.341, FDR: 0.1794
Methylation
1.00
0.75
0.50
0.25
0.00
Pre−T
Control−T
Exons
PKP2

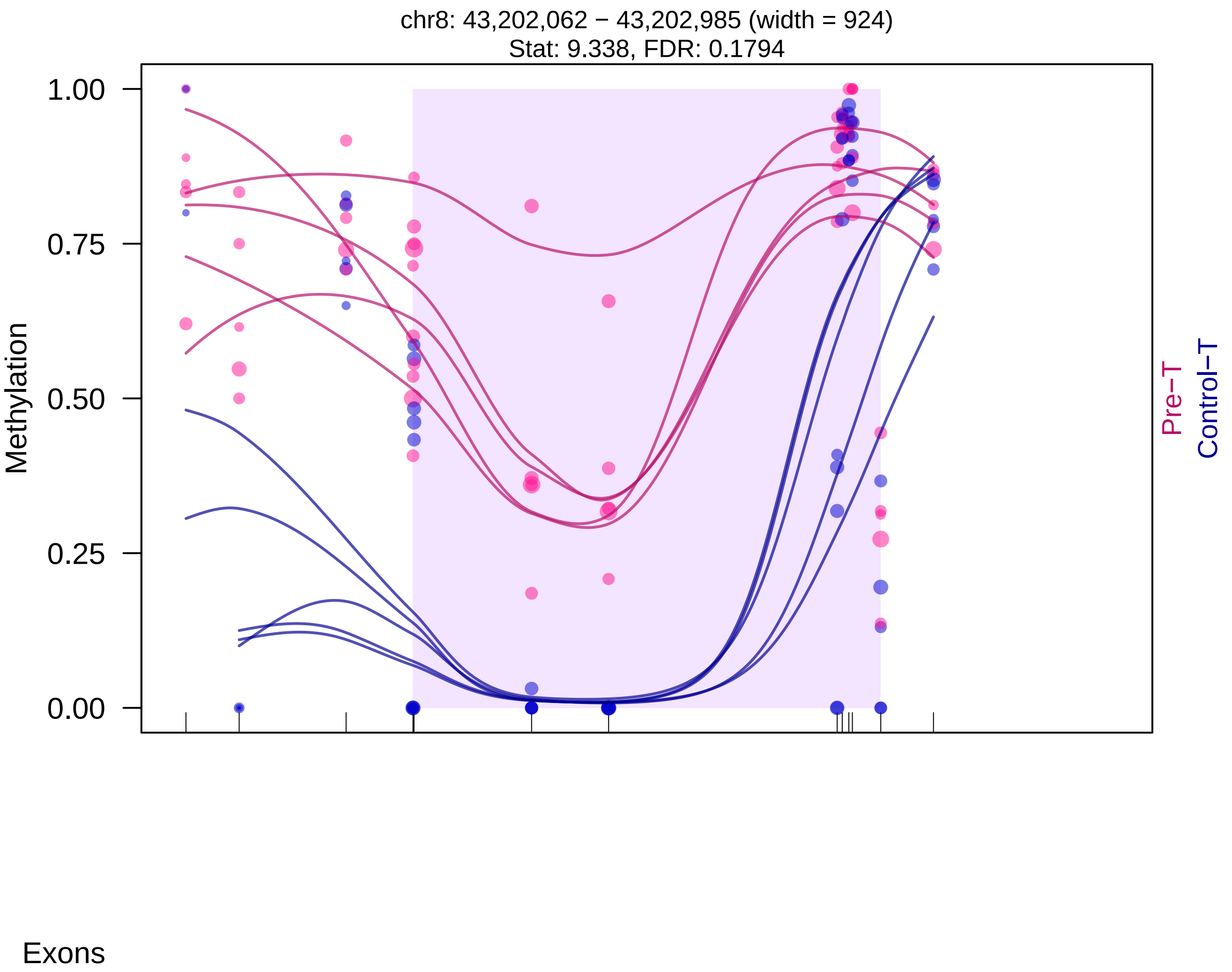

chr8: 43,202,062 − 43,202,985 (width = 924)
Stat: 9.338, FDR: 0.1794
Methylation
1.00
0.75
0.50
0.25
0.00
Pre−T
Control−T
Exons

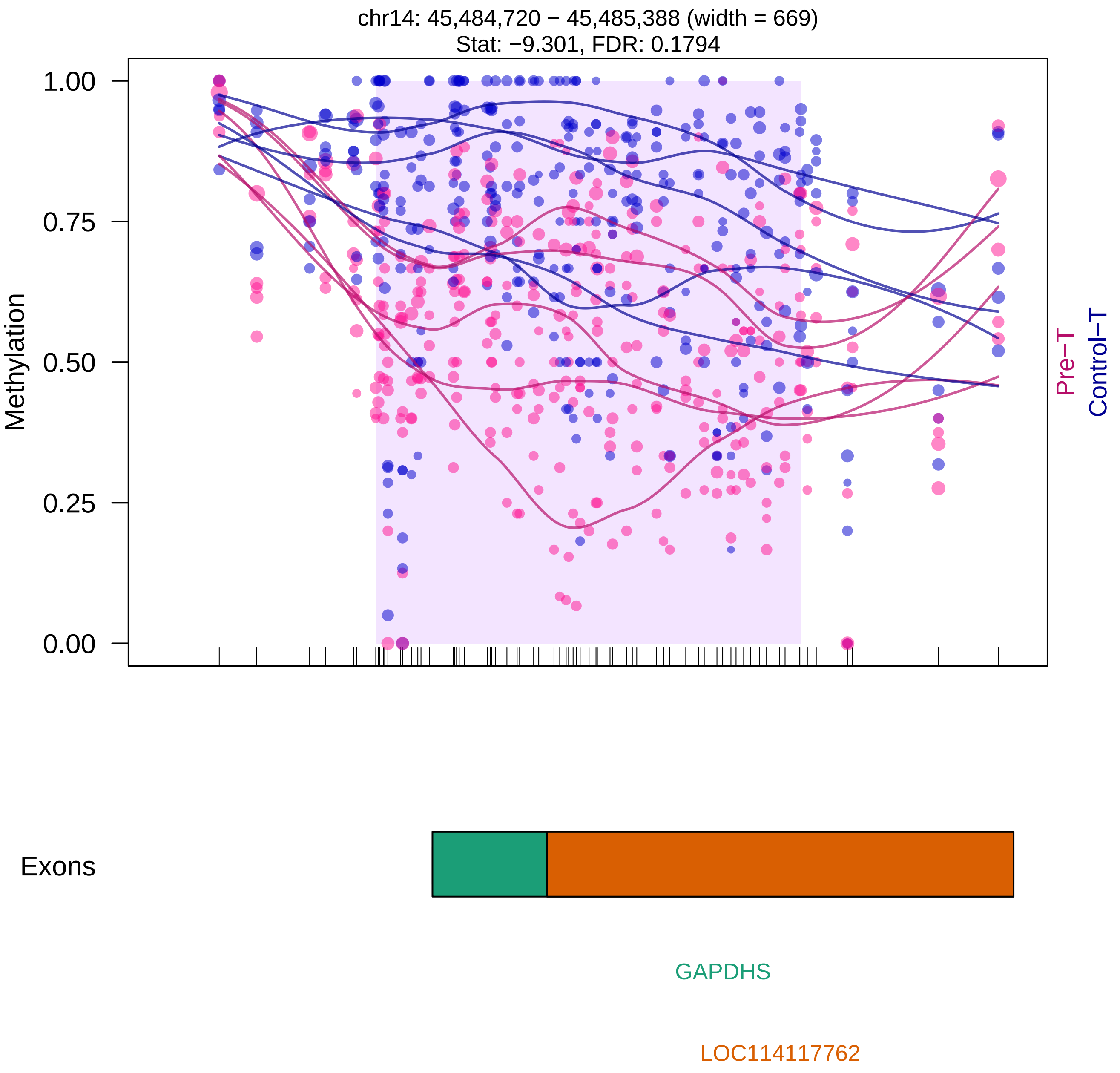
chr14: 45,484,720 − 45,485,388 (width = 669)
Stat: −9.301, FDR: 0.1794
Methylation
1.00
0.75
0.50
0.25
0.00
Pre−T
Control−T
Exons
GAPDHS
LOC114117762

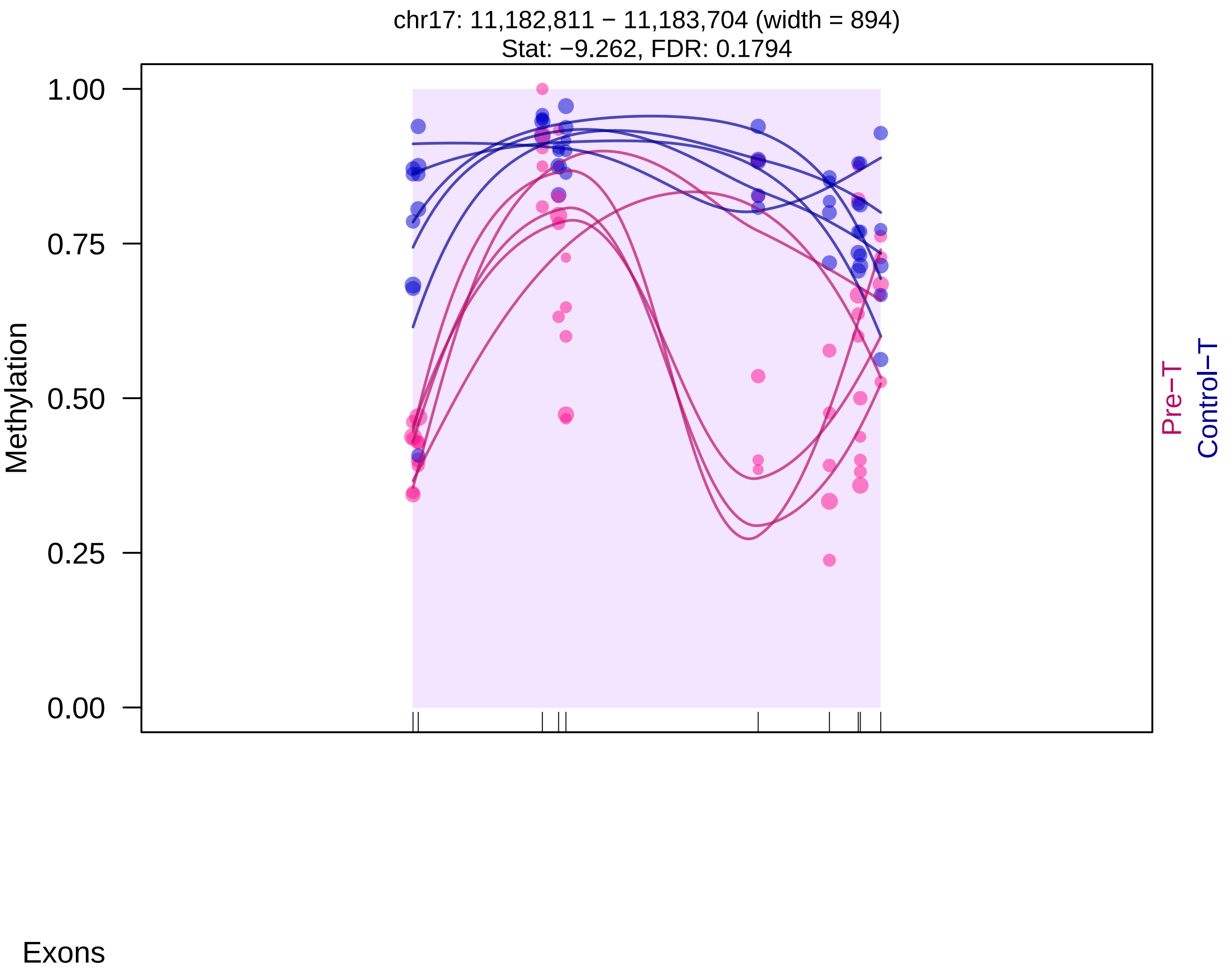

chr17: 11,182,811 − 11,183,704 (width = 894)
Stat: −9.262, FDR: 0.1794
1.00
0.75
0.50
0.25
0.00
Methylation
Pre−T
Control−T
Exons

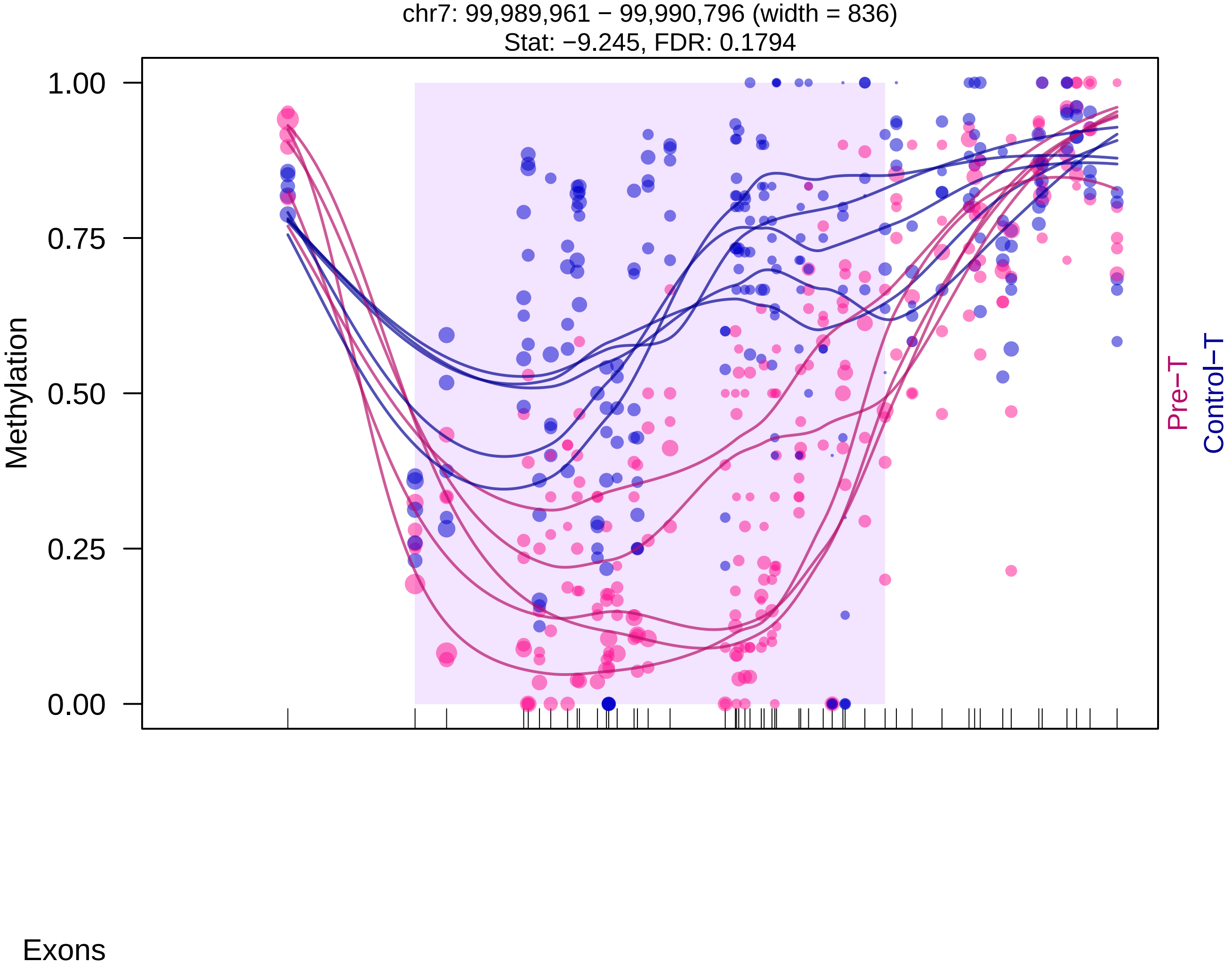
chr7: 99,989,961 − 99,990,796 (width = 836)
Stat: −9.245, FDR: 0.1794
1.00
0.75
0.50
0.25
0.00
Methylation
Pre−T
Control−T
Exons

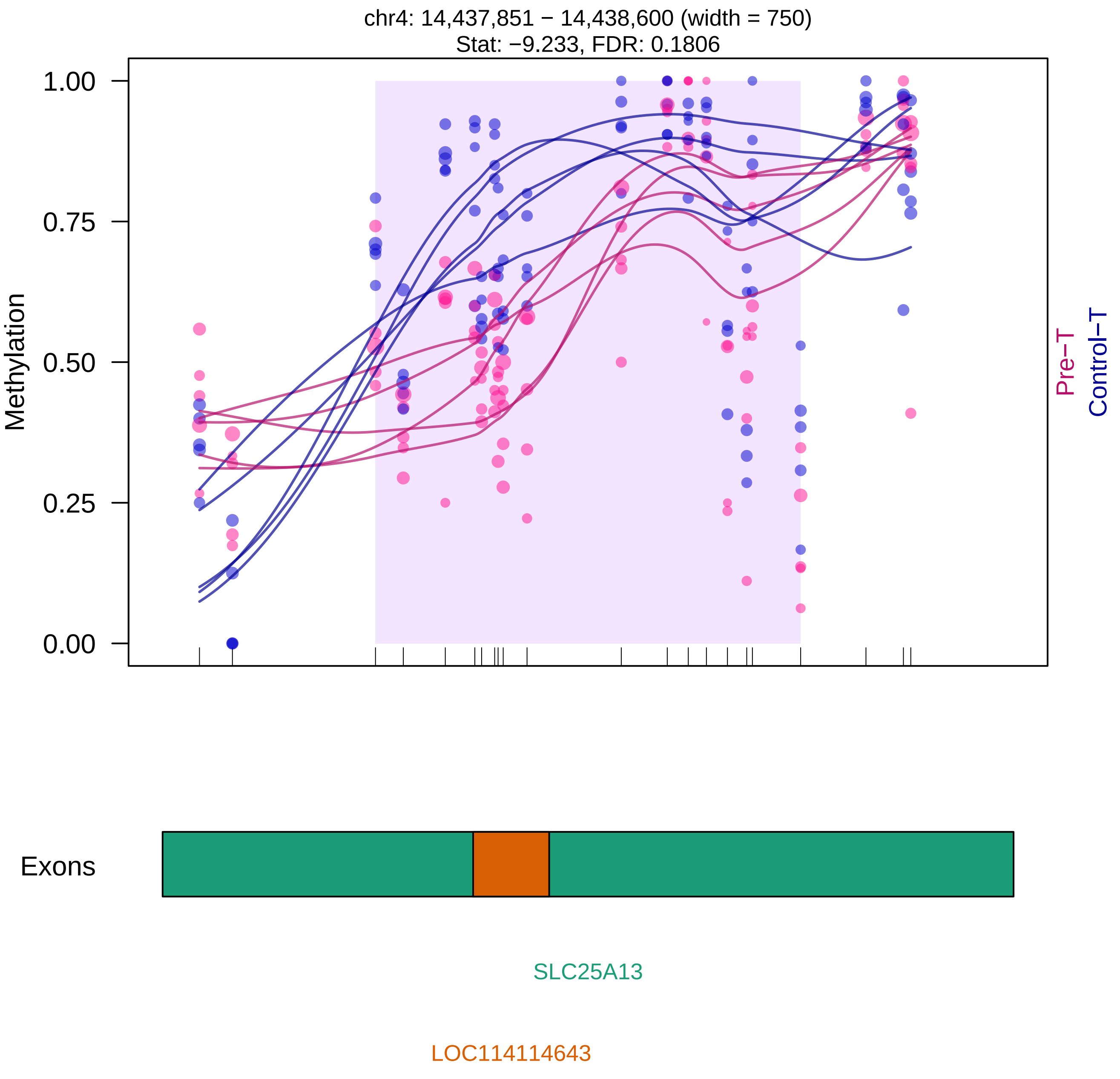
chr4: 14,437,851 − 14,438,600 (width = 750)
Stat: −9.233, FDR: 0.1806
Methylation
1.00
0.75
0.50
0.25
0.00
Pre−T
Control−T
Exons
SLC25A13
LOC114114643

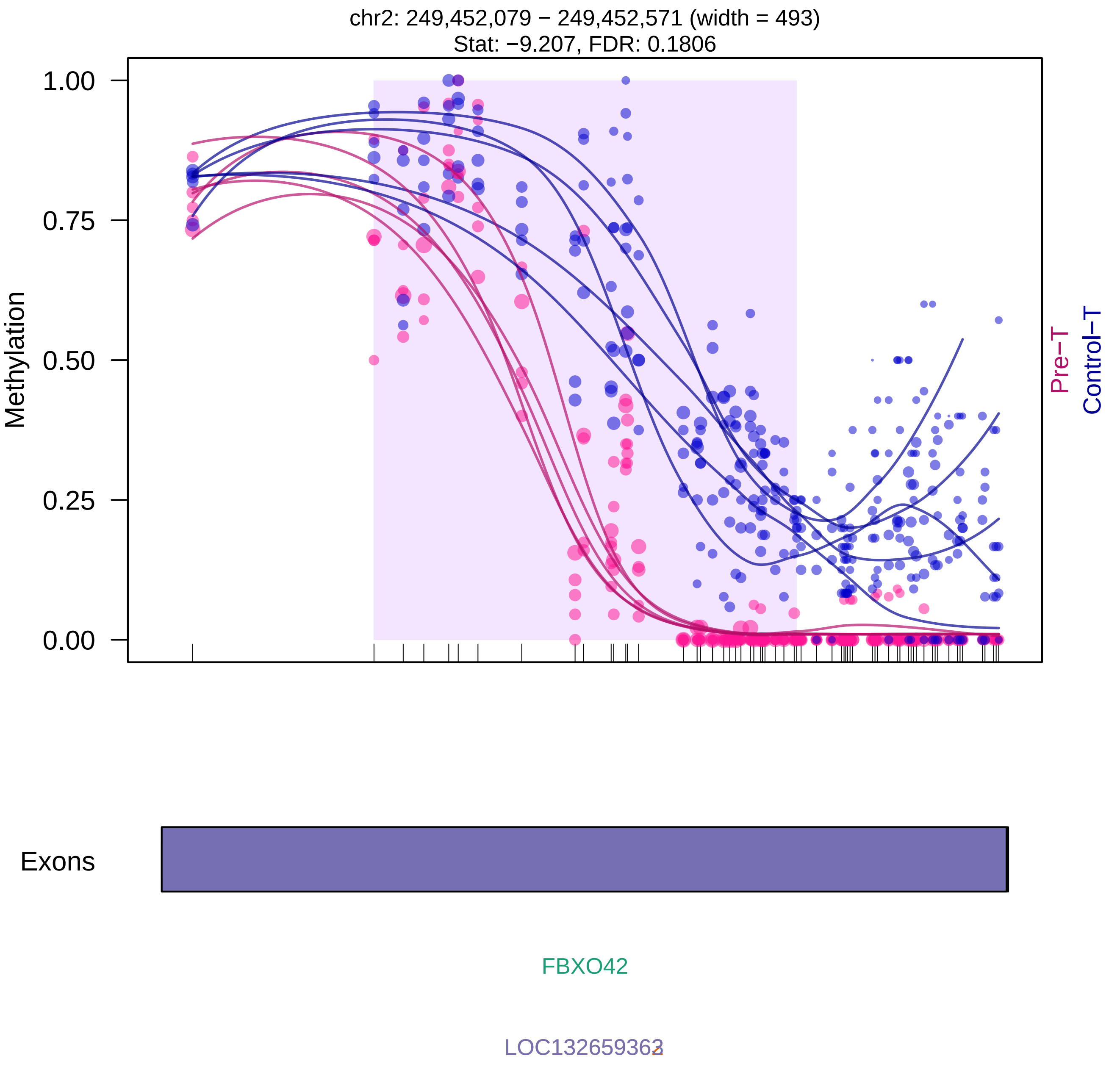
chr2: 249,452,079 − 249,452,571 (width = 493)
Stat: −9.207, FDR: 0.1806
1.00
0.75
0.50
0.25
0.00
Methylation
Pre−T
Control−T
Exons
FBXO42
LOC132659362

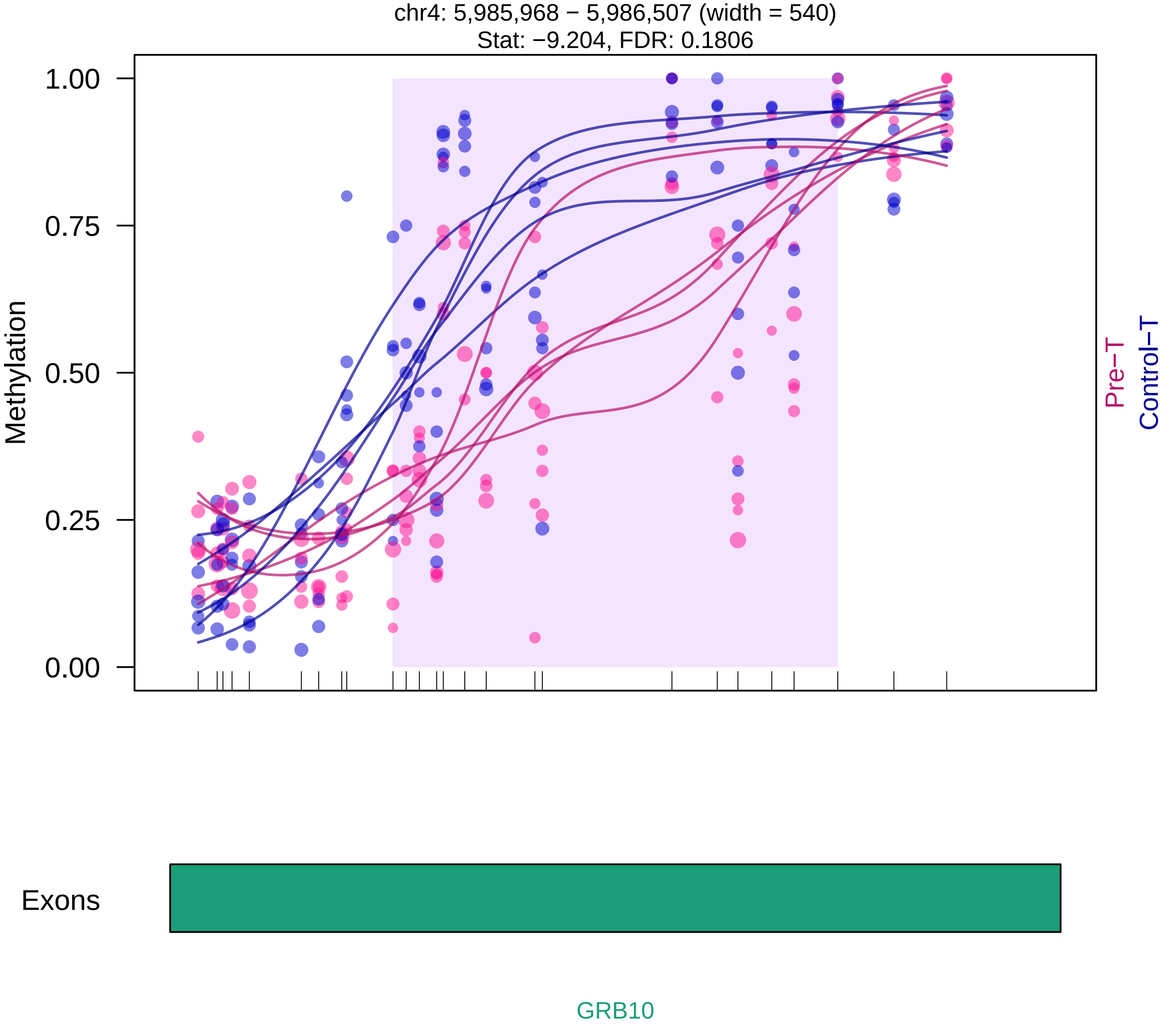
chr4: 5,985,968 − 5,986,507 (width = 540)
Stat: −9.204, FDR: 0.1806
1.00
0.75
0.50
0.25
0.00
Methylation
Pre−T
Control−T
Exons
GRB10

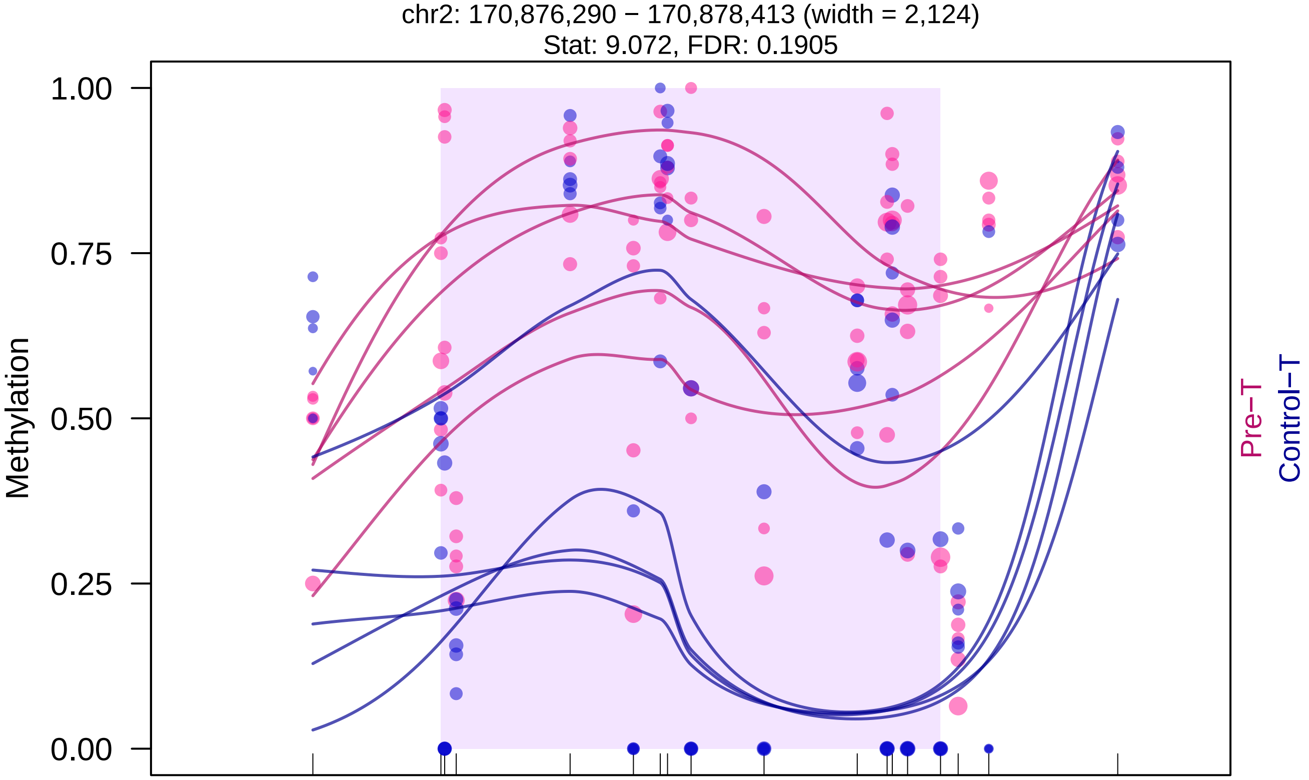

chr2: 170,876,290 − 170,878,413 (width = 2,124)
Stat: 9.072, FDR: 0.1905
1.00
0.75
0.50
0.25
0.00
Methylation
Pre−T
Control−T

Exons

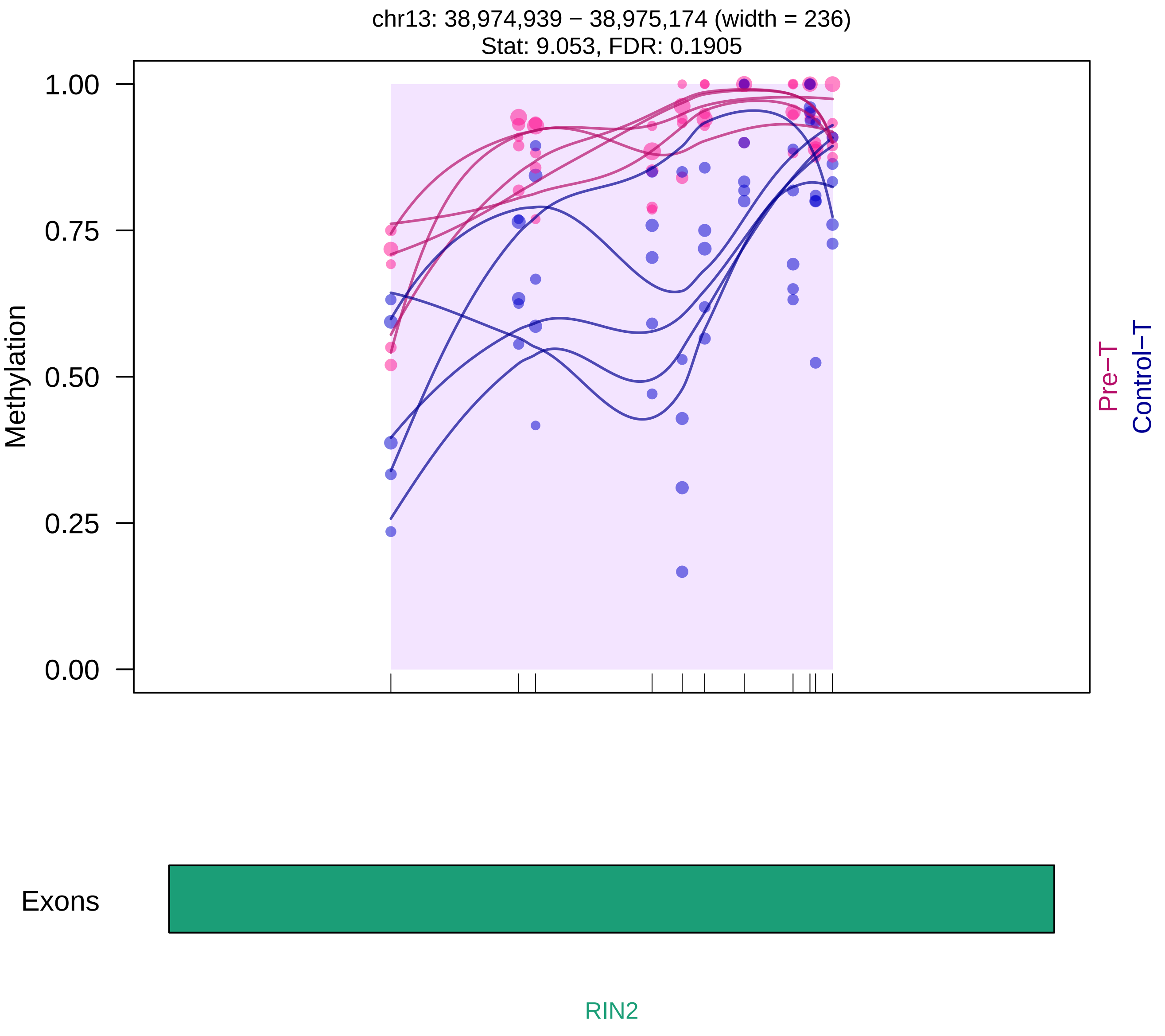
chr13: 38,974,939 − 38,975,174 (width = 236)
Stat: 9.053, FDR: 0.1905
Methylation
1.00
0.75
0.50
0.25
0.00
Pre−T
Control−T
Exons
RIN2

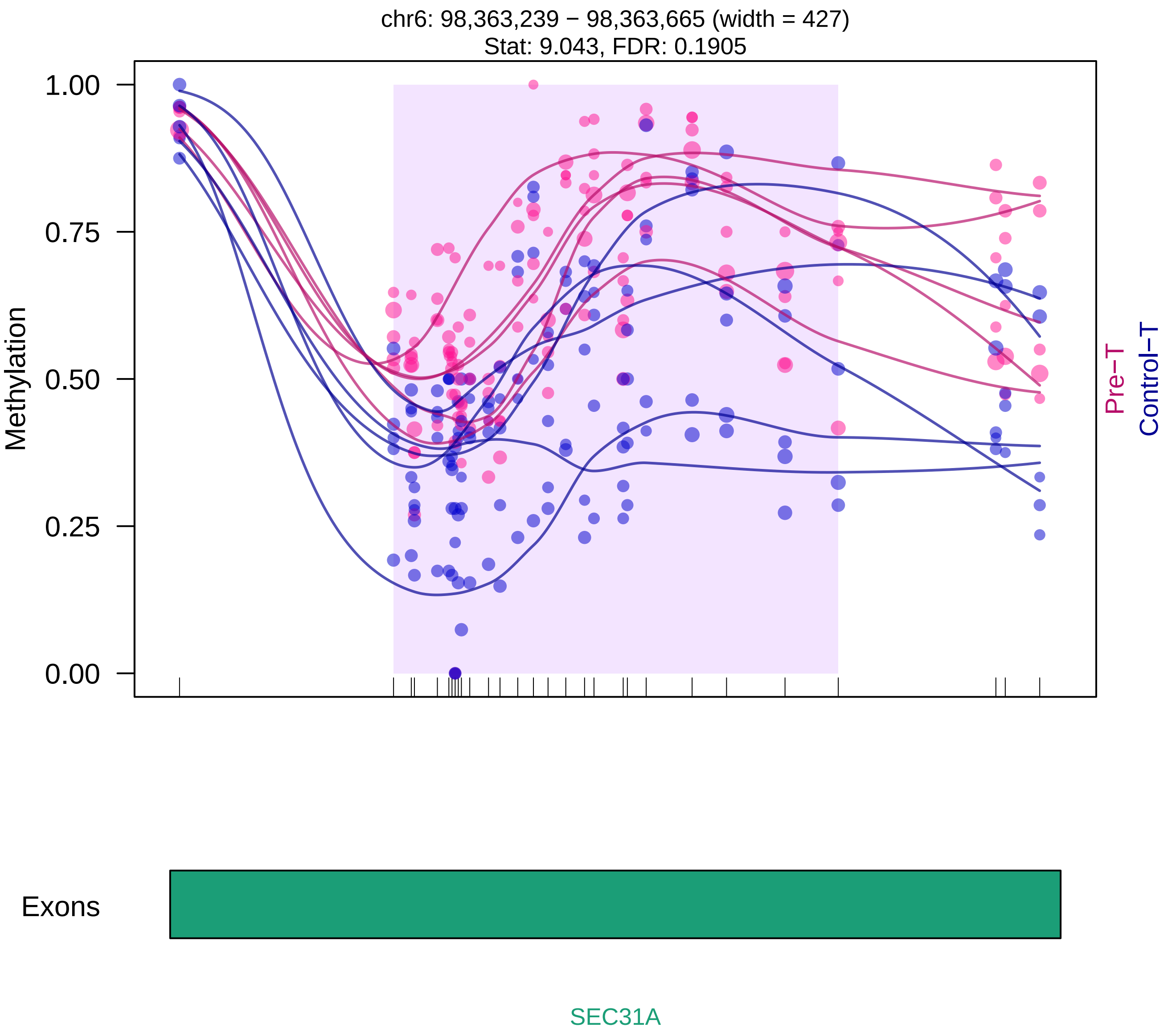

chr6: 98,363,239 − 98,363,665 (width = 427)
Stat: 9.043, FDR: 0.1905
Methylation
1.00
0.75
0.50
0.25
0.00
Pre−T
Control−T
Exons
SEC31A

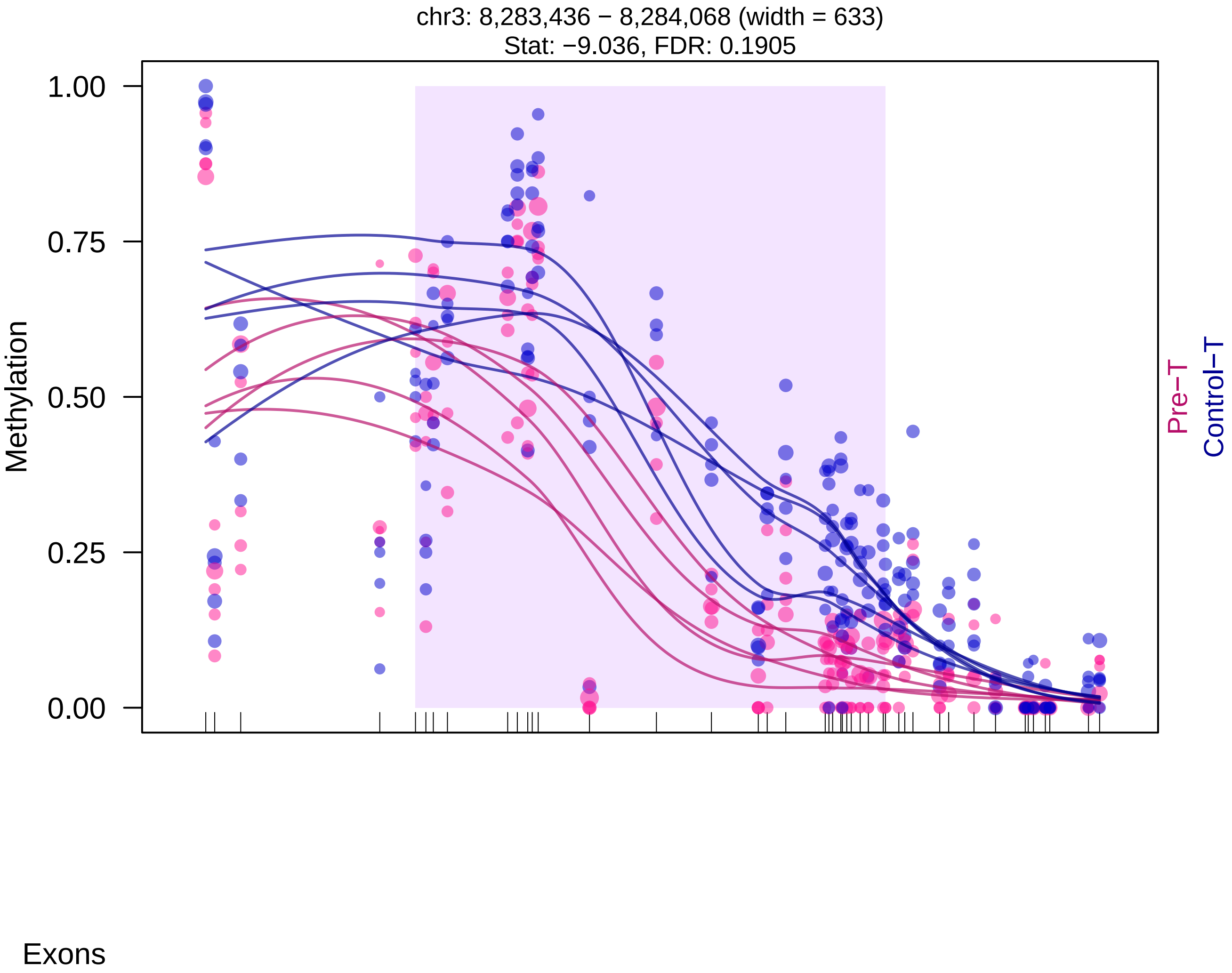
chr3: 8,283,436 − 8,284,068 (width = 633)
Stat: −9.036, FDR: 0.1905
1.00
0.75
0.50
0.25
0.00
Methylation
Pre−T
Control−T
Exons

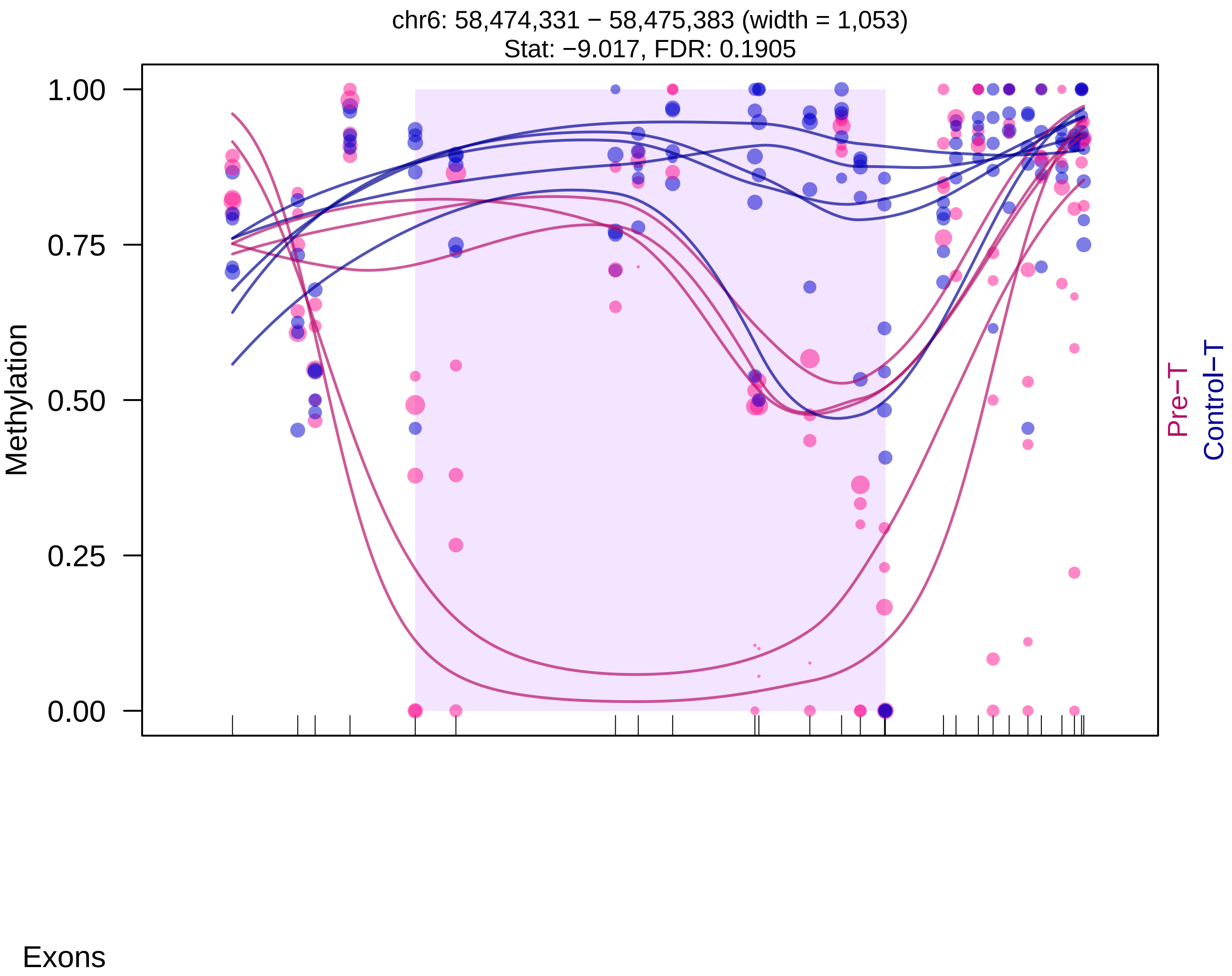

chr6: 58,474,331 − 58,475,383 (width = 1,053)
Stat: −9.017, FDR: 0.1905
1.00
0.75
0.50
0.25
0.00
Methylation
Pre−T
Control−T
Exons

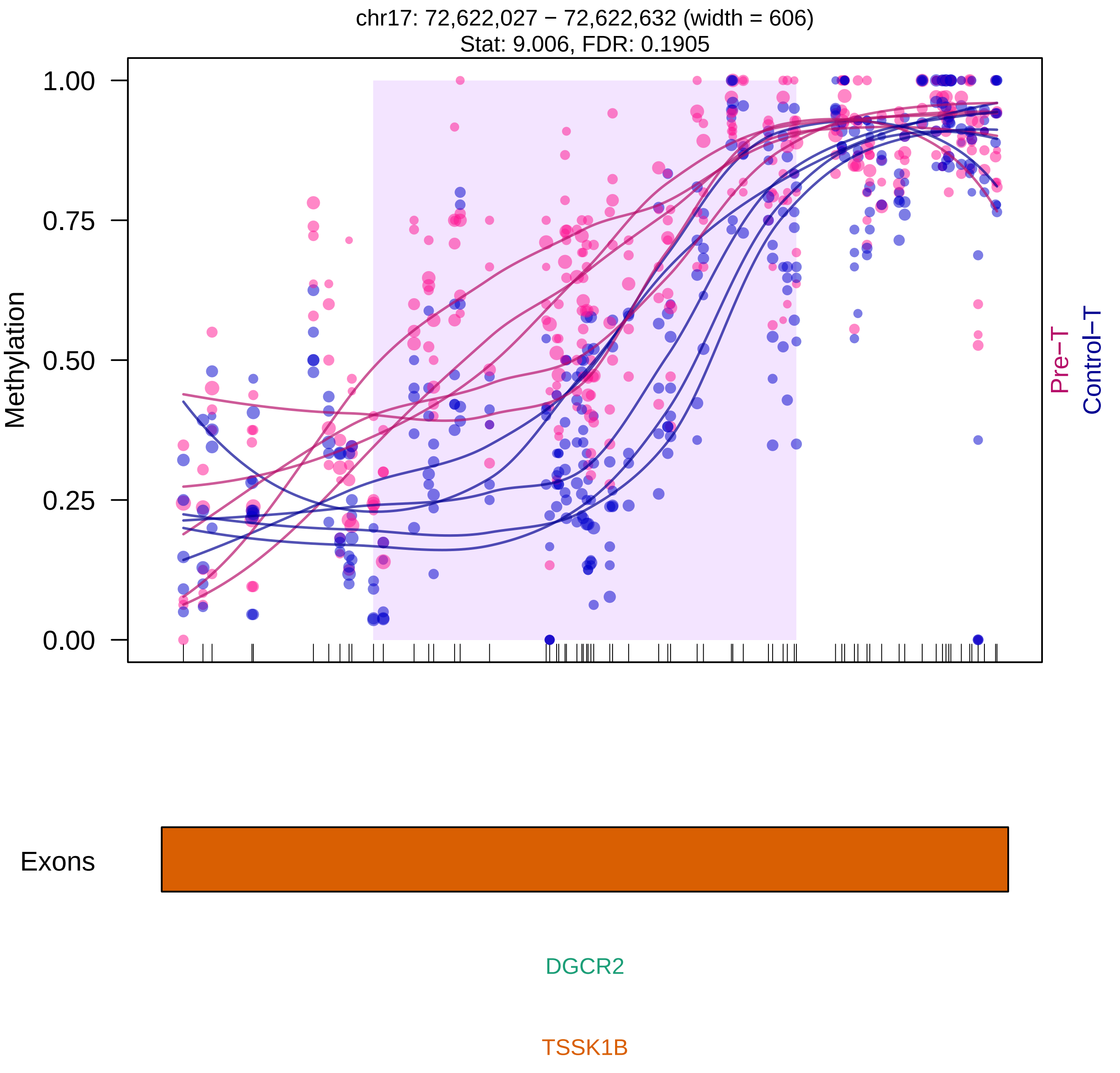

chr17: 72,622,027 − 72,622,632 (width = 606)
Stat: 9.006, FDR: 0.1905
Methylation
1.00
0.75
0.50
0.25
0.00
Pre−T
Control−T
Exons
DGCR2
TSSK1B

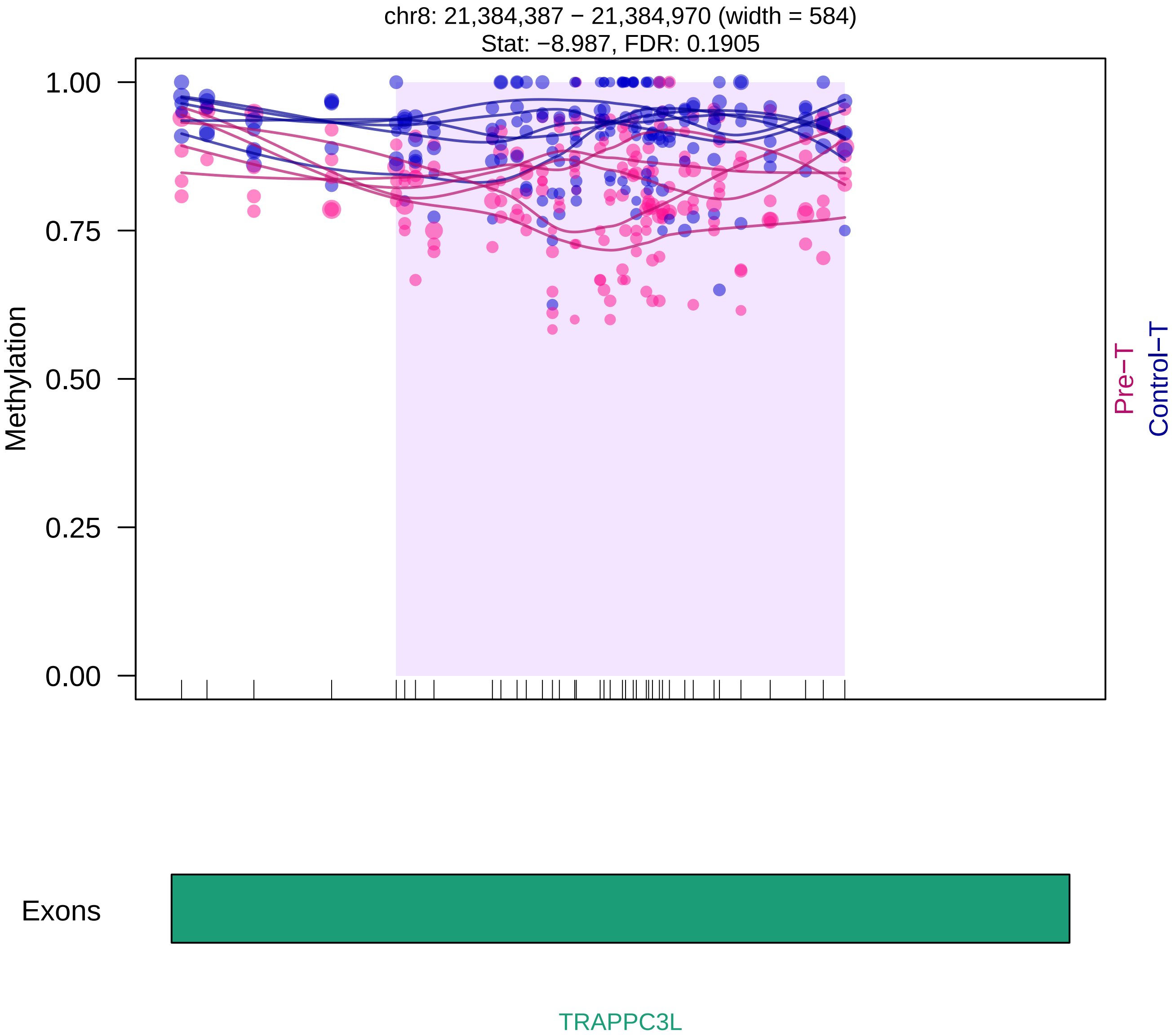
chr8: 21,384,387 − 21,384,970 (width = 584)
Stat: −8.987, FDR: 0.1905
Methylation
1.00
0.75
0.50
0.25
0.00
Pre−T
Control−T
Exons
TRAPPC3L

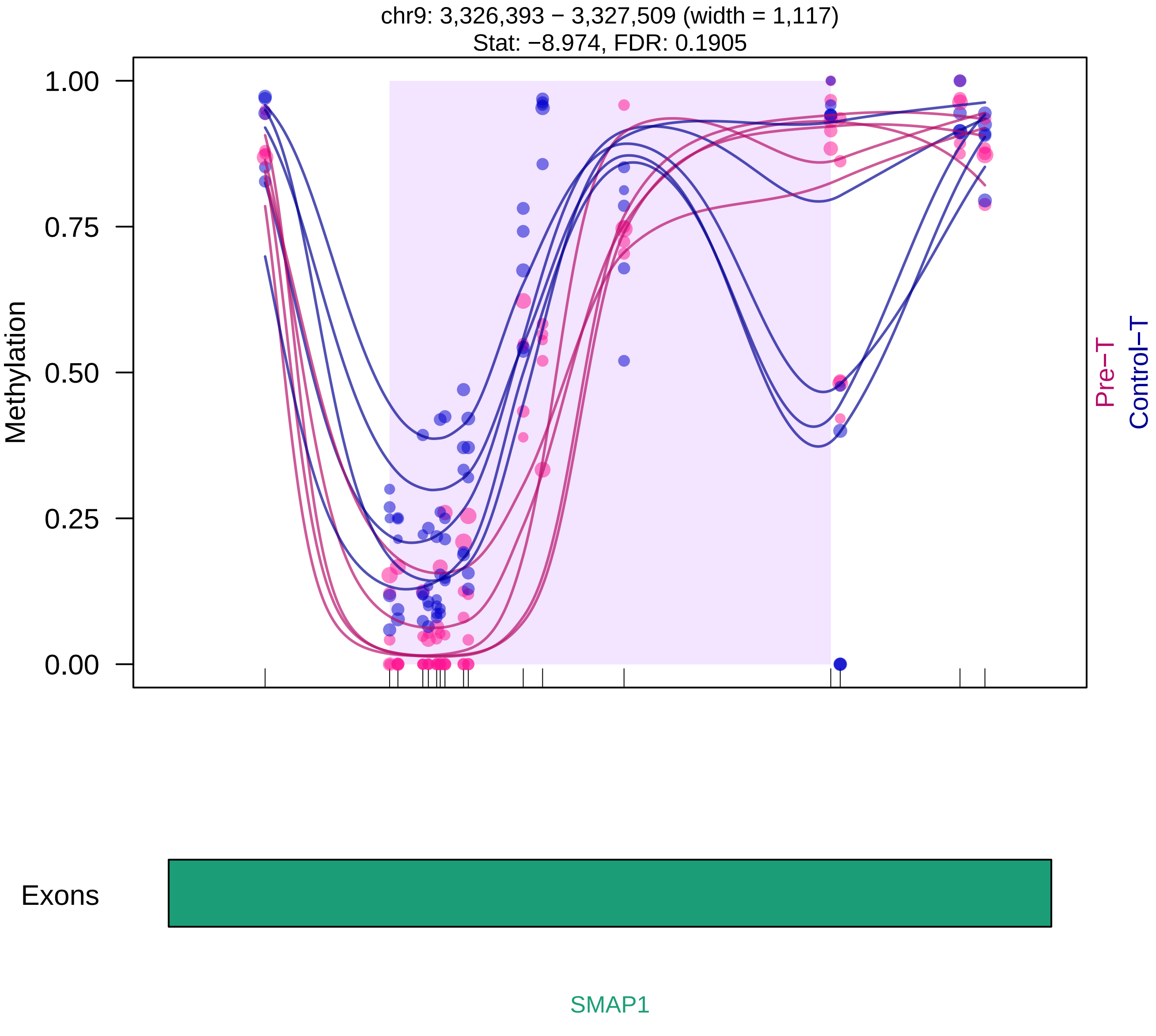

chr9: 3,326,393 − 3,327,509 (width = 1,117)
Stat: −8.974, FDR: 0.1905
Methylation
1.00
0.75
0.50
0.25
0.00
Pre−T
Control−T
Exons
SMAP1

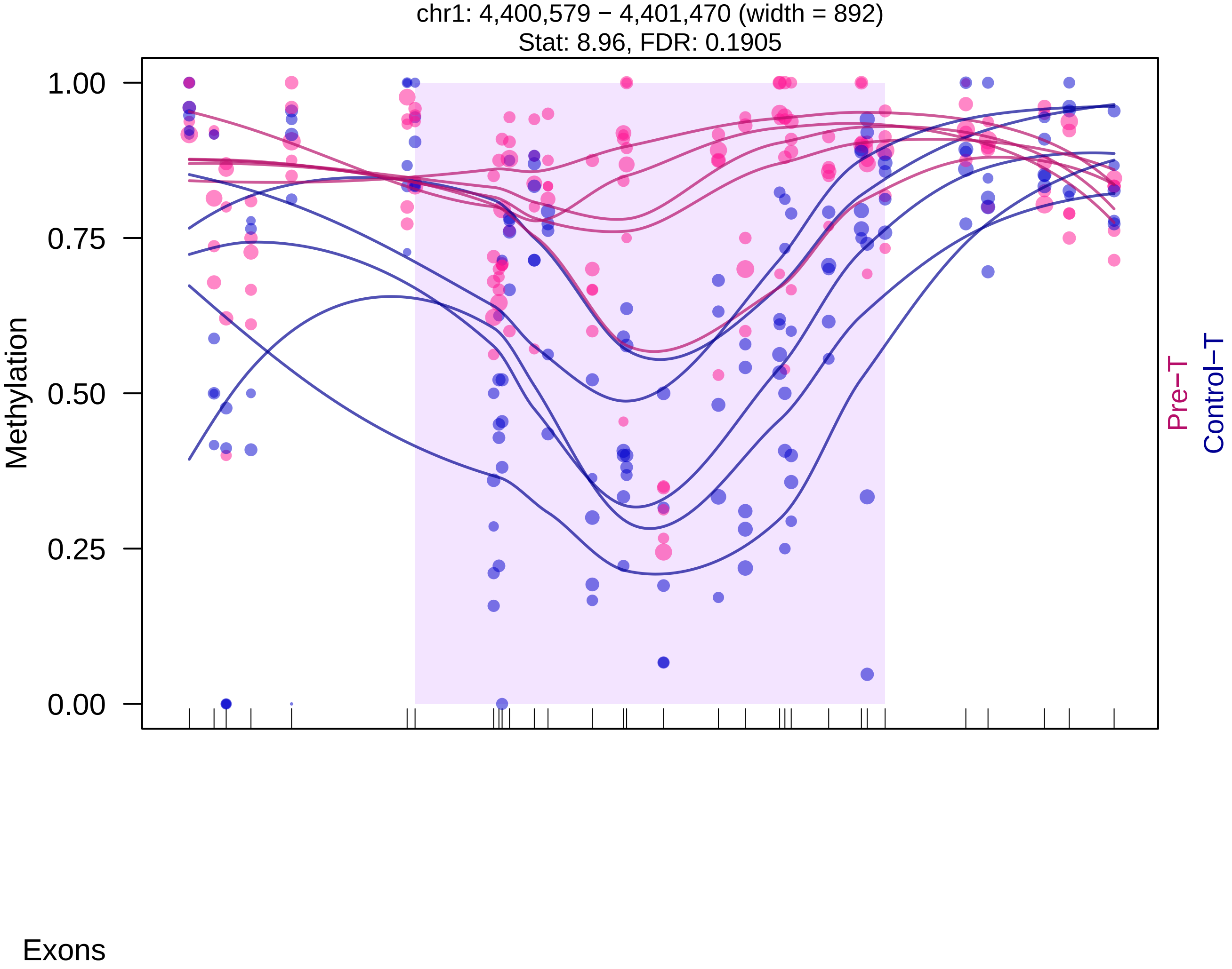

chr1: 4,400,579 − 4,401,470 (width = 892)
Stat: 8.96, FDR: 0.1905
1.00
0.75
0.50
0.25
0.00
Methylation
Pre−T
Control−T
Exons

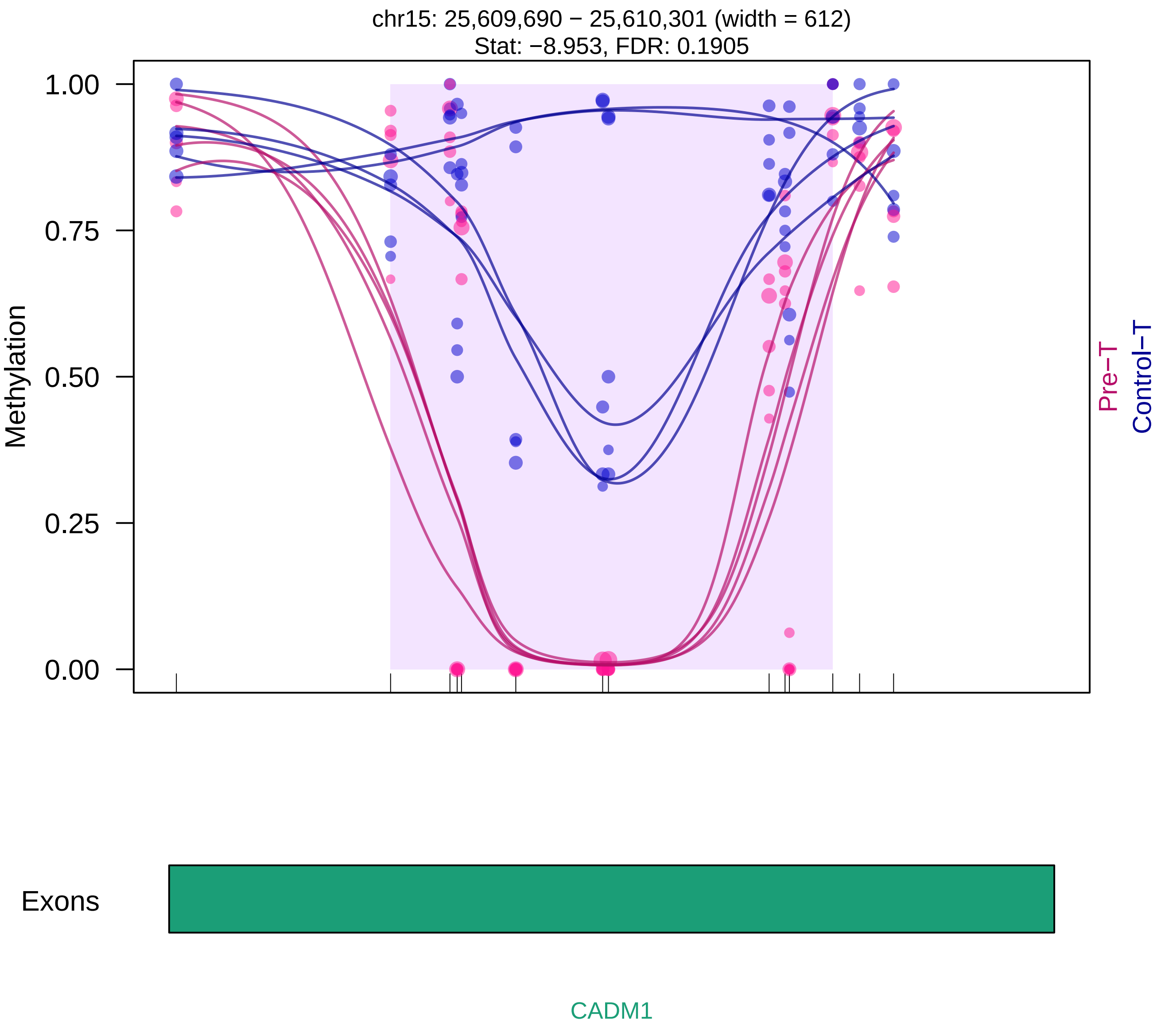

chr15: 25,609,690 − 25,610,301 (width = 612)
Stat: −8.953, FDR: 0.1905
1.00
0.75
0.50
0.25
0.00
Methylation
Pre−T
Control−T
Exons
CADM1

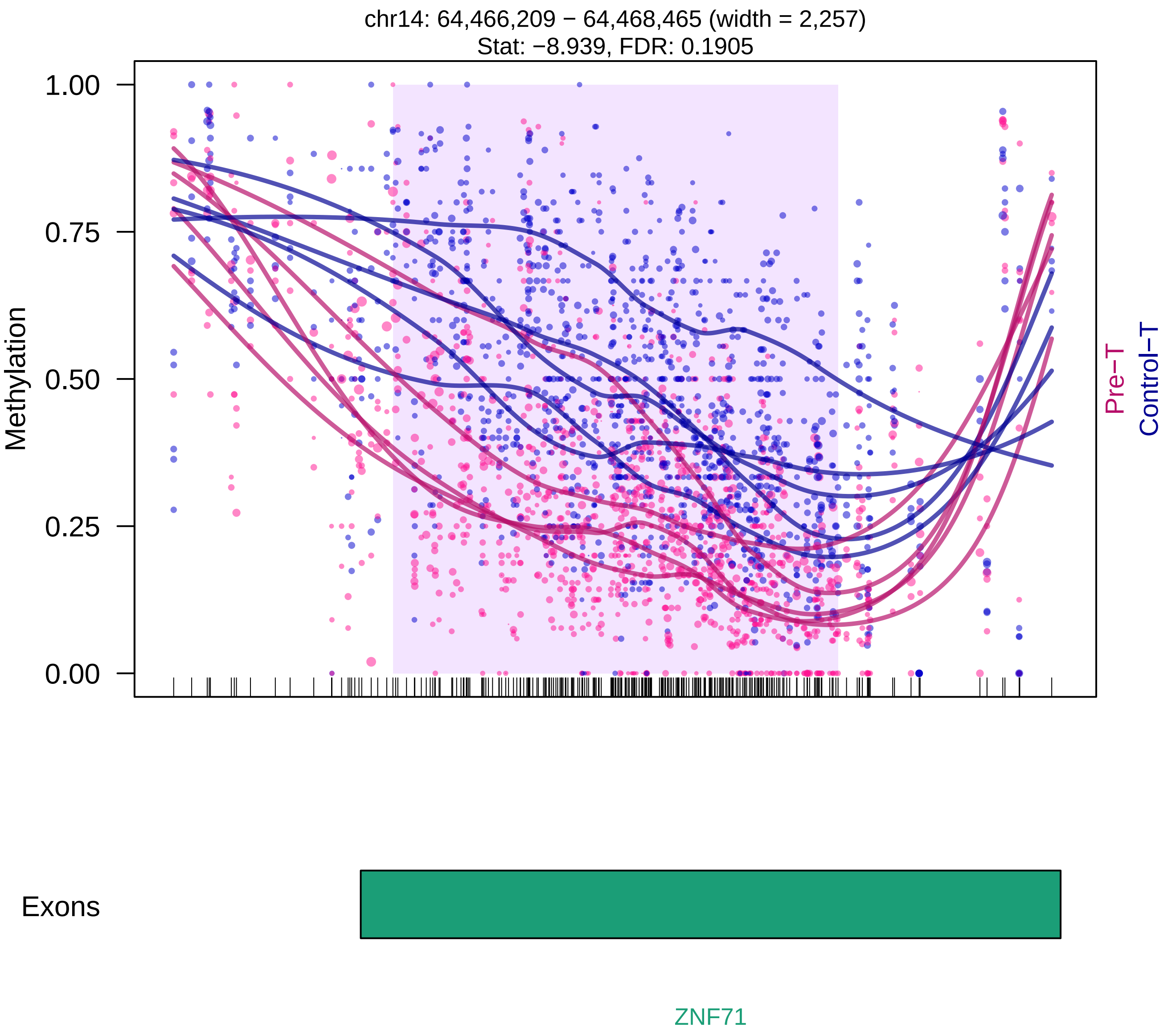
chr14: 64,466,209 − 64,468,465 (width = 2,257)
Stat: −8.939, FDR: 0.1905
Methylation
1.00
0.75
0.50
0.25
0.00
Pre−T
Control−T
Exons
ZNF71

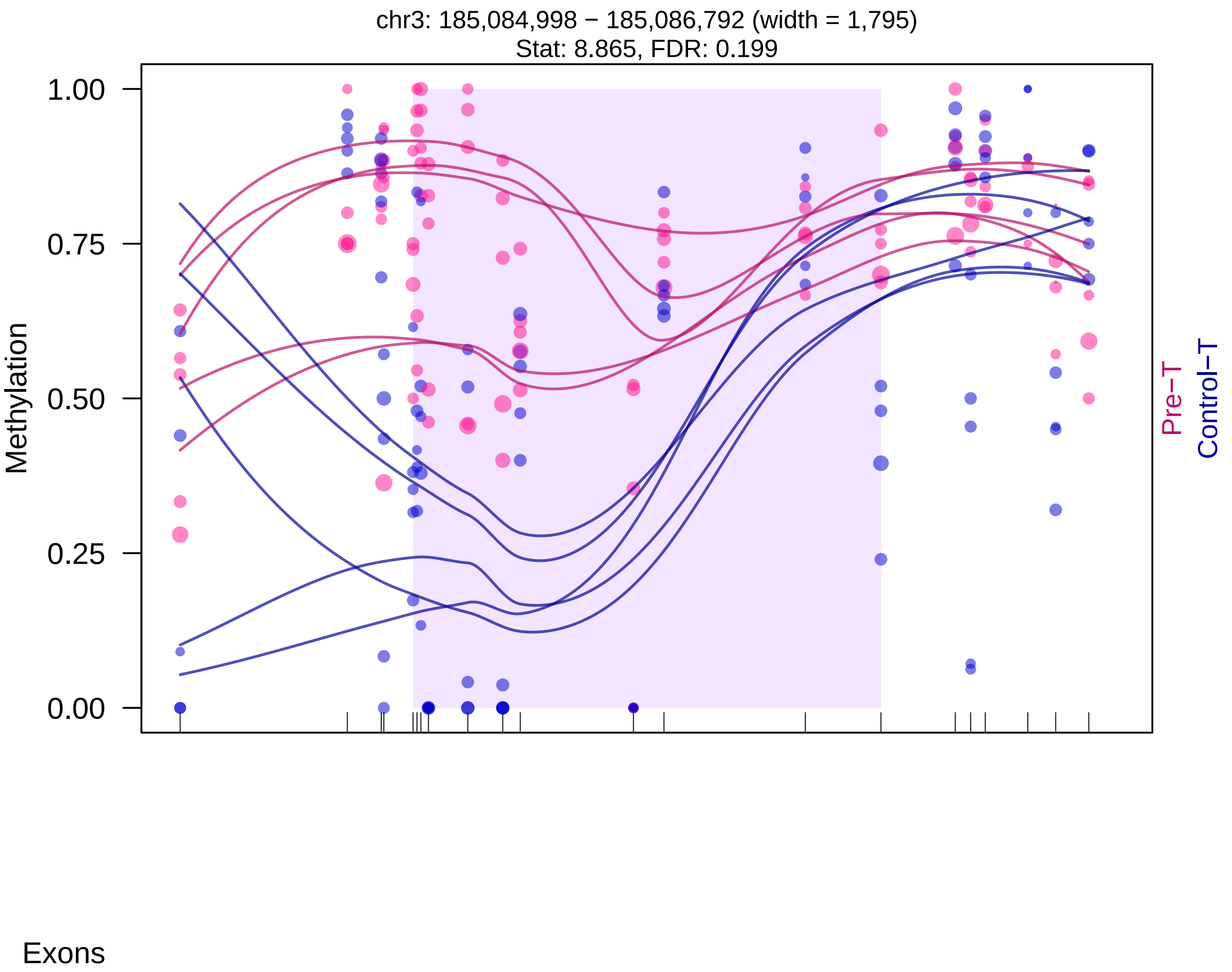

chr3: 185,084,998 − 185,086,792 (width = 1,795)
Stat: 8.865, FDR: 0.199
Methylation
1.00
0.75
0.50
0.25
0.00
Pre−T
Control−T
Exons

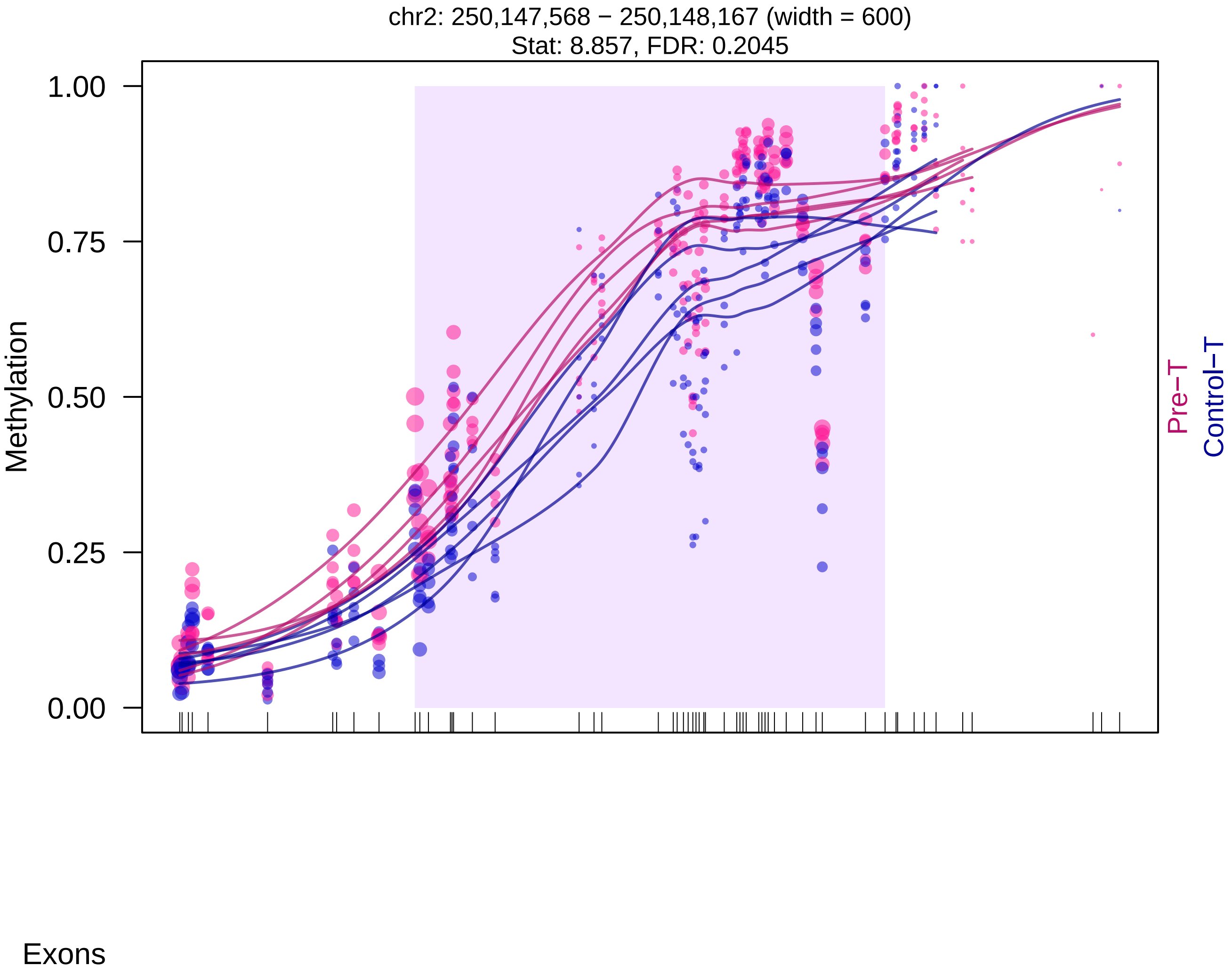
chr2: 250,147,568 − 250,148,167 (width = 600)
Stat: 8.857, FDR: 0.2045
Methylation
1.00
0.75
0.50
0.25
0.00
Pre−T
Control−T
Exons

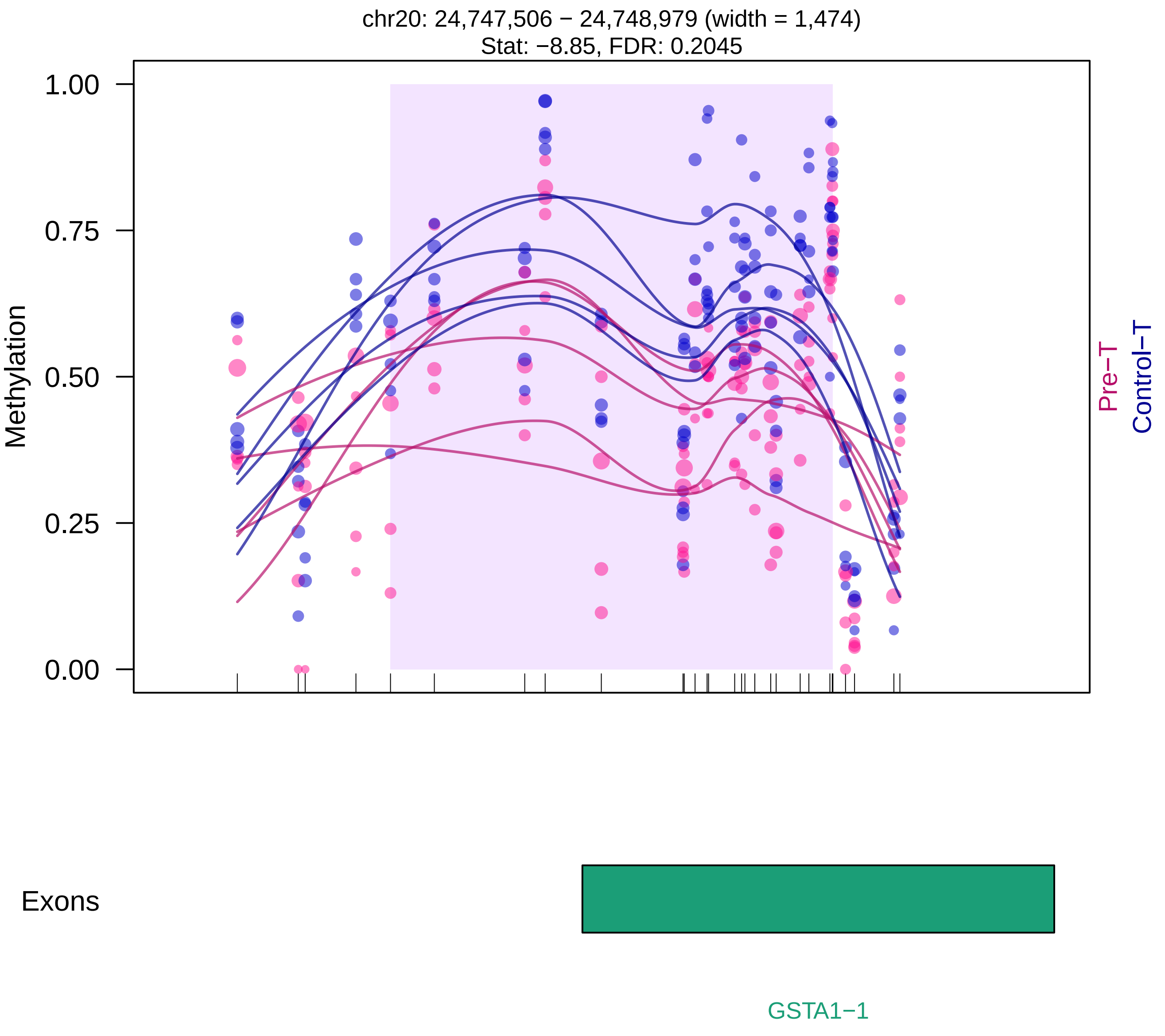
chr20: 24,747,506 − 24,748,979 (width = 1,474)
Stat: −8.85, FDR: 0.2045
1.00
0.75
0.50
0.25
0.00
Methylation
Pre−T
Control−T
Exons
GSTA1−1

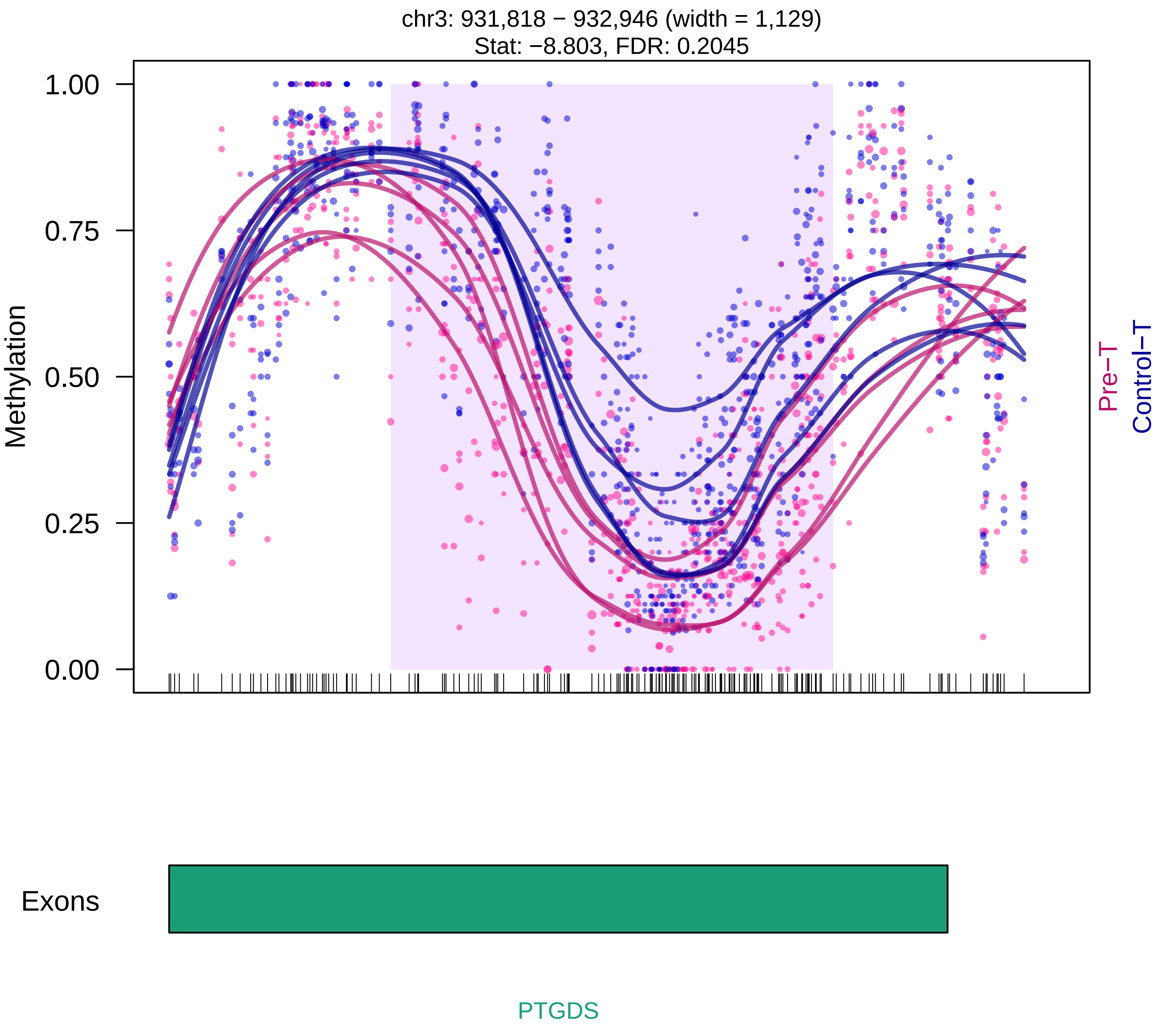

chr3: 931,818 − 932,946 (width = 1,129)
Stat: −8.803, FDR: 0.2045
Methylation
1.00
0.75
0.50
0.25
0.00
Pre−T
Control−T
Exons
PTGDS

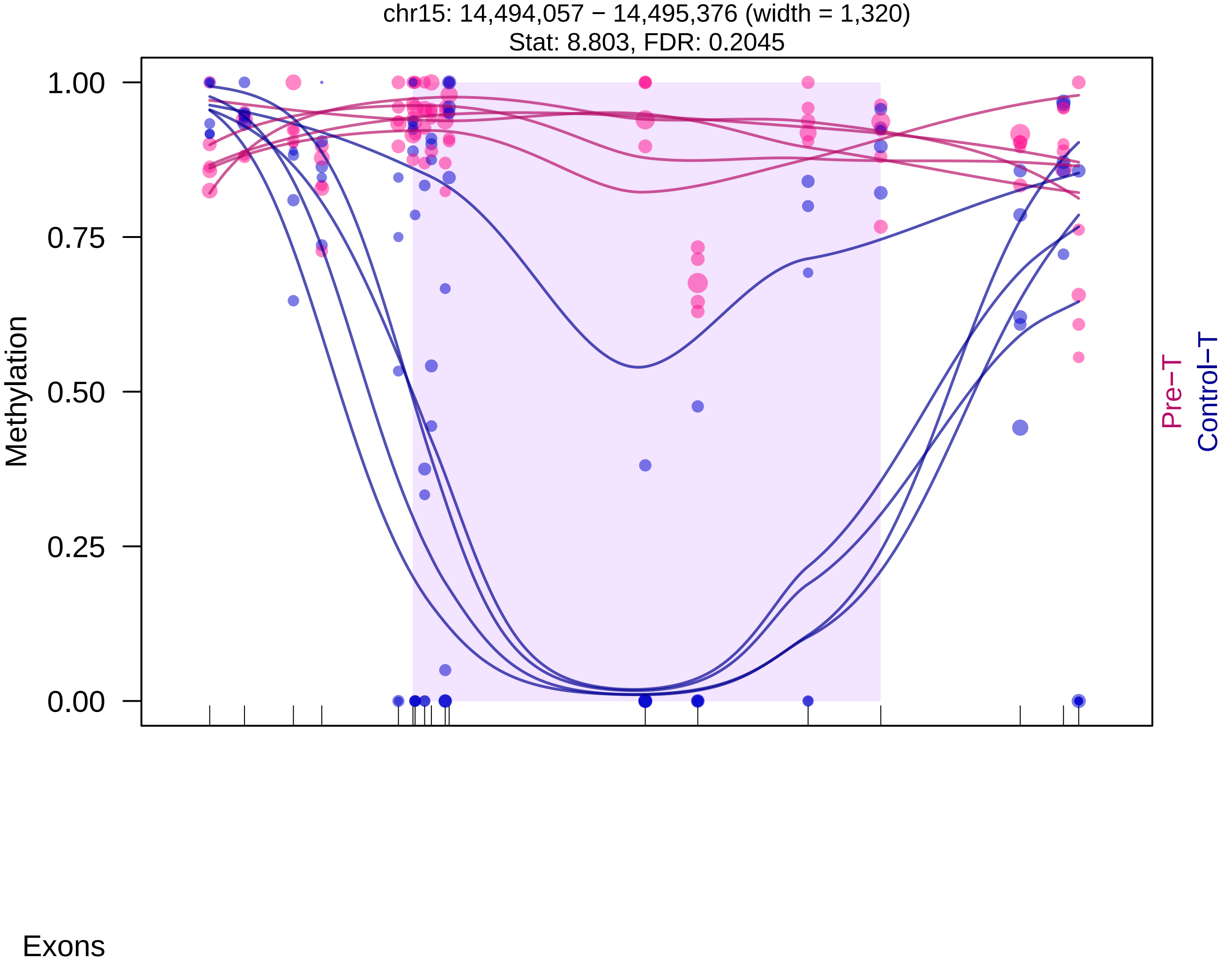

chr15: 14,494,057 − 14,495,376 (width = 1,320)
Stat: 8.803, FDR: 0.2045
Methylation
1.00
0.75
0.50
0.25
0.00
Pre−T
Control−T
Exons

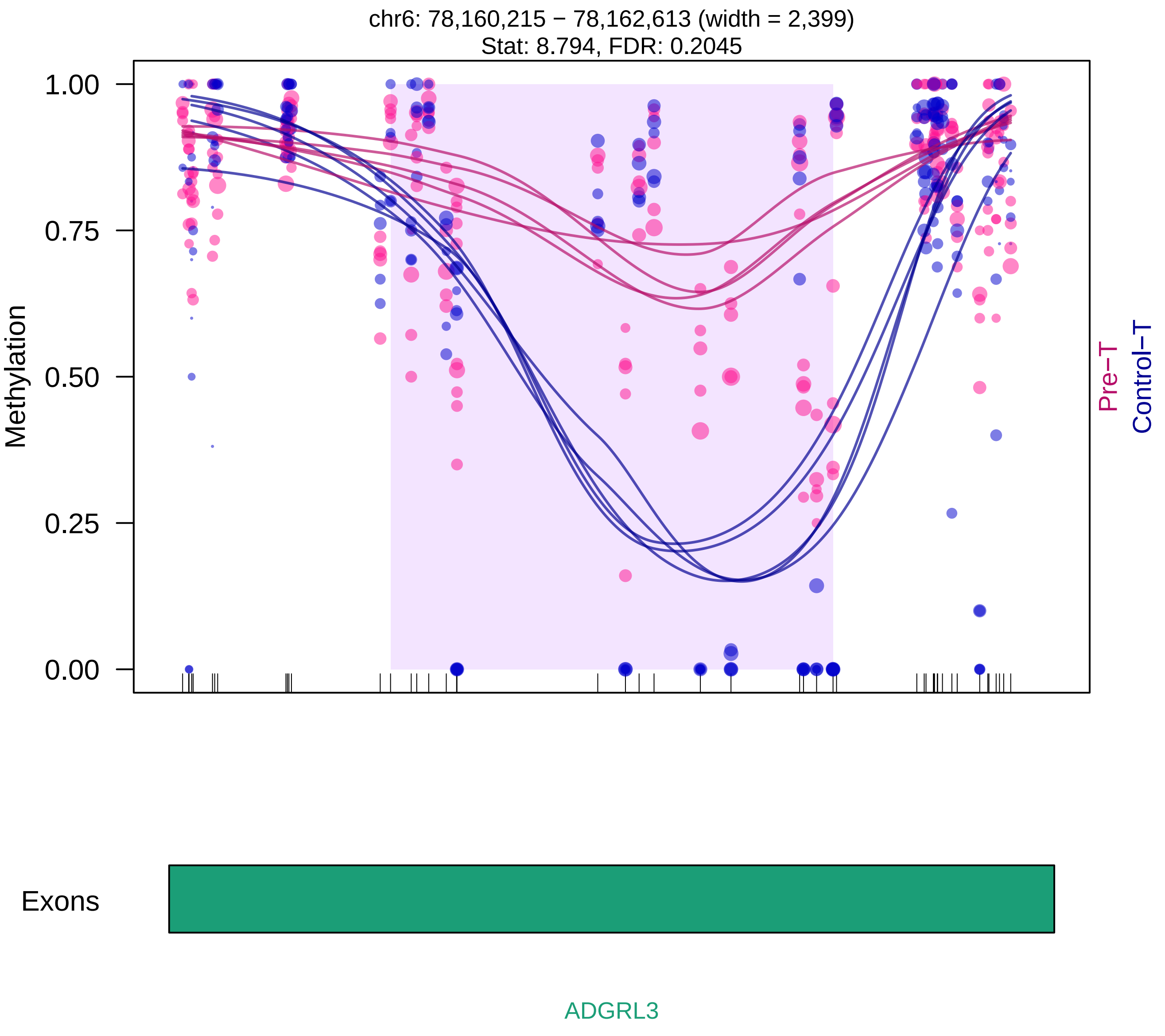
chr6: 78,160,215 − 78,162,613 (width = 2,399)
Stat: 8.794, FDR: 0.2045
1.00
0.75
0.50
0.25
0.00
Methylation
Pre−T
Control−T
Exons
ADGRL3

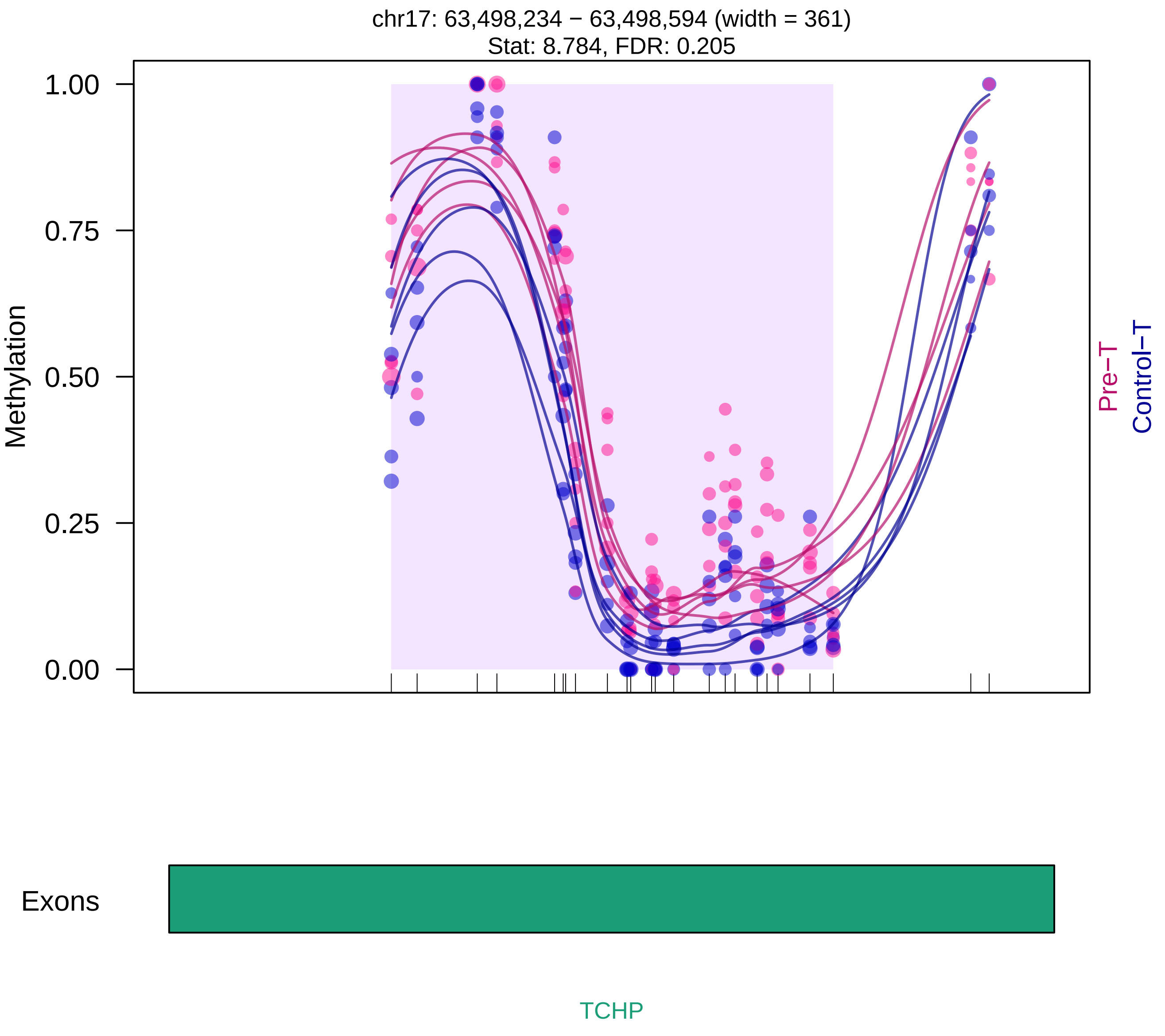
chr17: 63,498,234 − 63,498,594 (width = 361)
Stat: 8.784, FDR: 0.205
Methylation
1.00
0.75
0.50
0.25
0.00
Pre−T
Control−T
Exons
TCHP

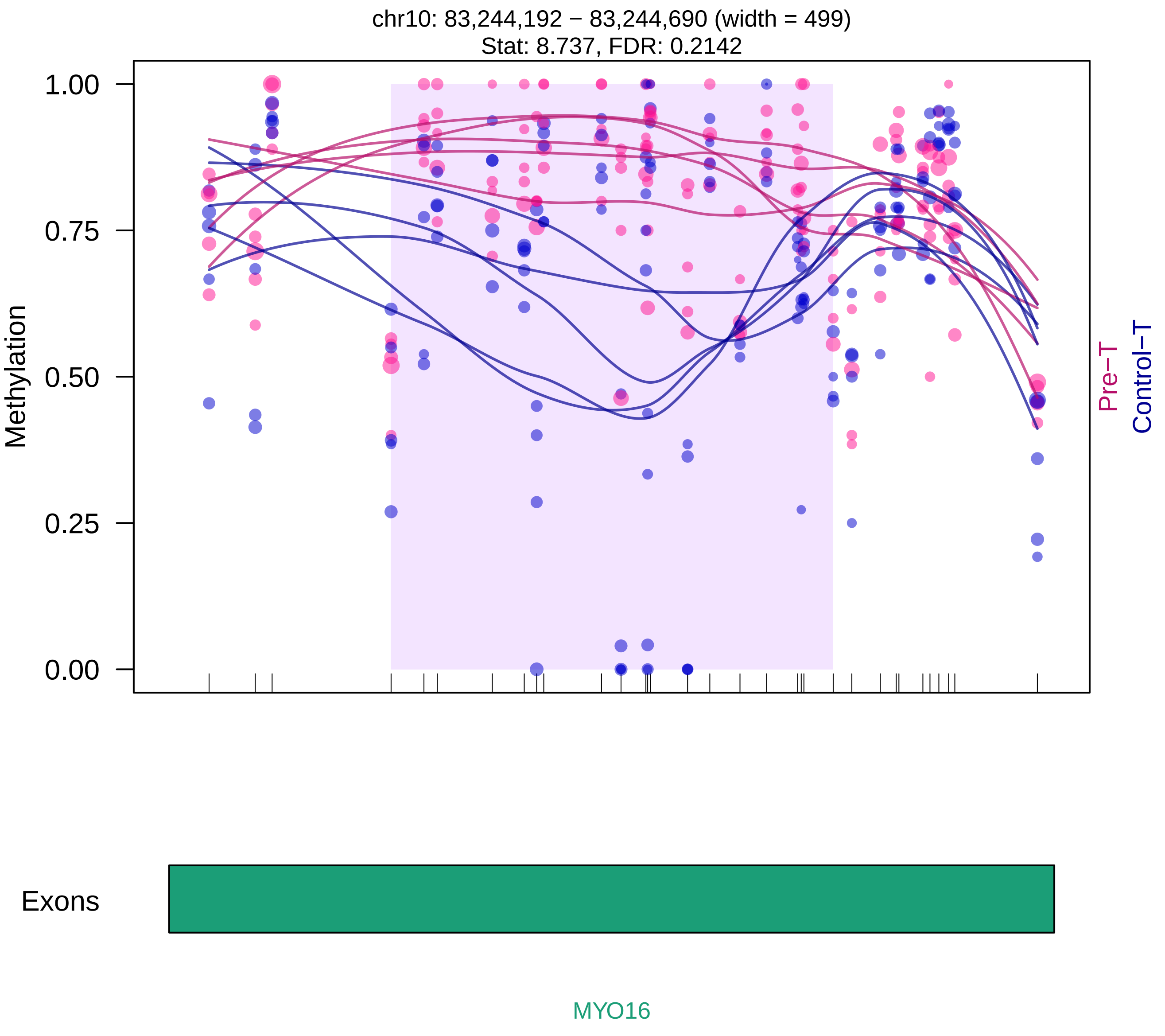

chr10: 83,244,192 − 83,244,690 (width = 499)
Stat: 8.737, FDR: 0.2142
Methylation
1.00
0.75
0.50
0.25
0.00
Pre−T
Control−T
Exons
MYO16
