# Supplementary material for: Developmental programming: Differing impact of prenatal testosterone and prenatal bisphenol-A -treatment on hepatic methylome in female sheep
Source: Mol Cell Endocrinol. Author manuscript; Available in PMC 2025 Dec 1. (PMC12668269; doi:10.1016/j.mce.2025.112655)
Supplement: Supplementary File 2 [file NIHMS2117496-supplement-Supplementary_File_2.pdf]

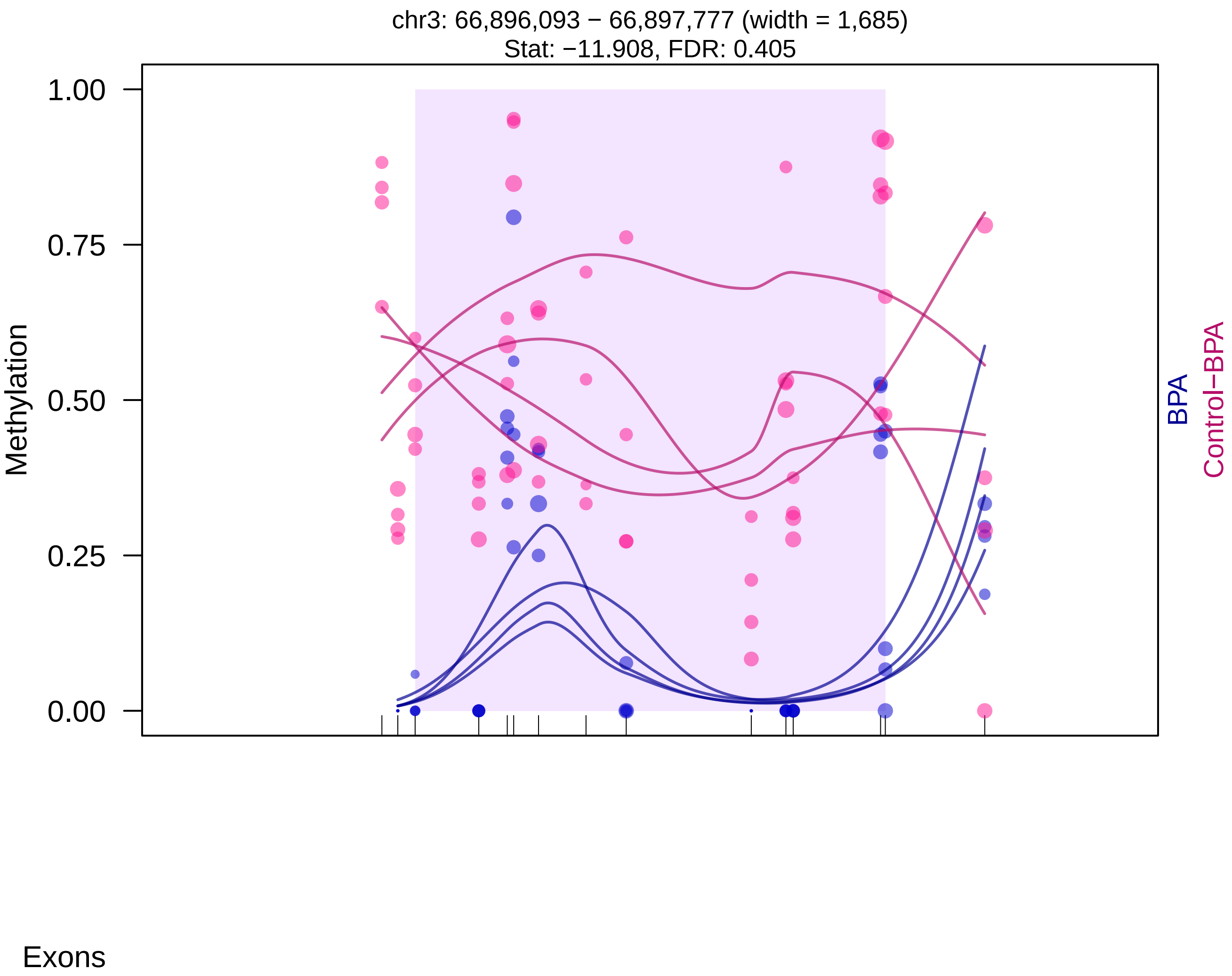
chr3: 66,896,093 − 66,897,777 (width = 1,685)
Stat: −11.908, FDR: 0.405
1.00
0.75
0.50
0.25
0.00
Methylation
BPA
Control−BPA
Exons

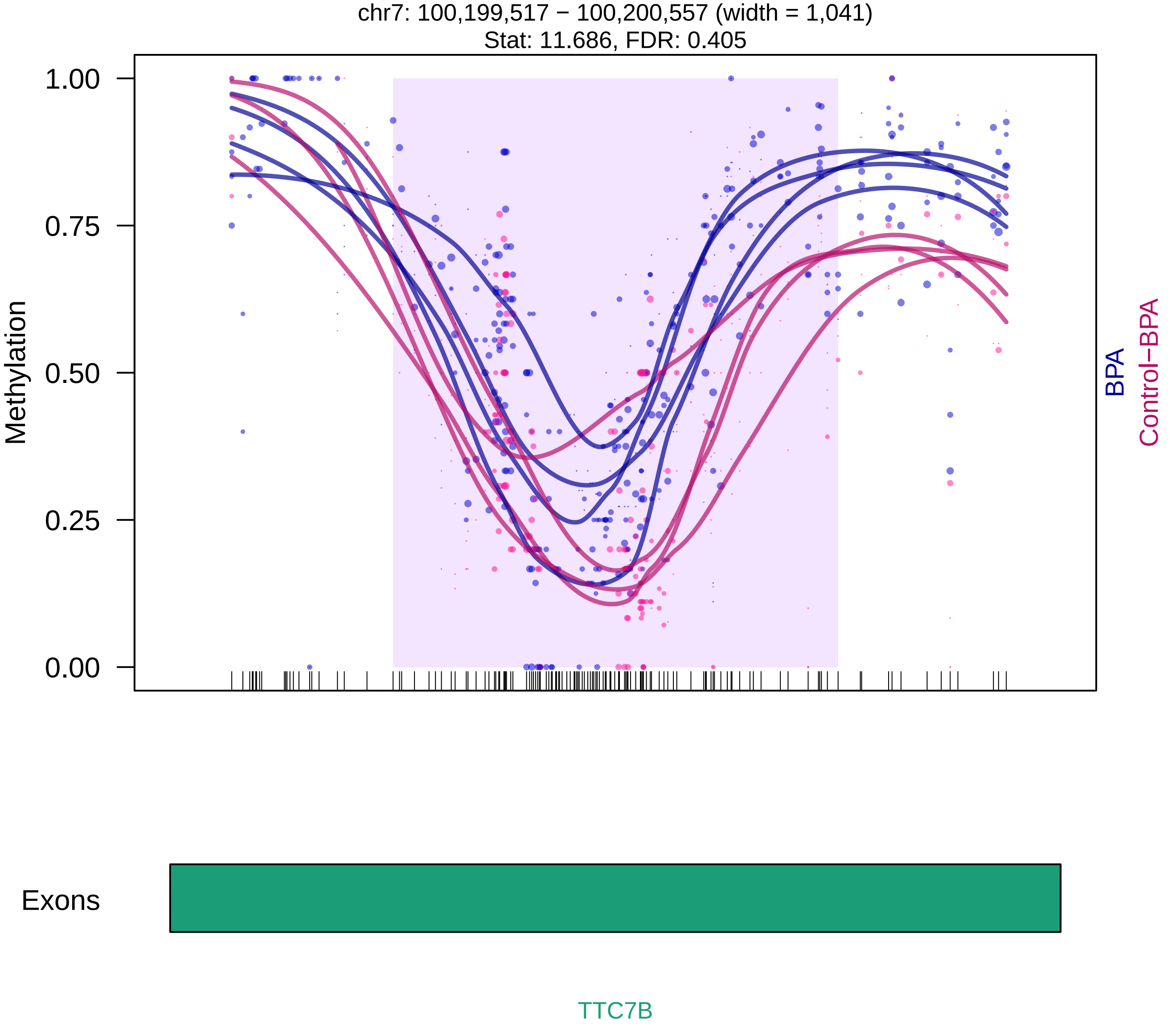
chr7: 100,199,517 − 100,200,557 (width = 1,041)
Stat: 11.686, FDR: 0.405
1.00
0.75
0.50
0.25
0.00
Methylation
BPA
Control−BPA
Exons
TTC7B

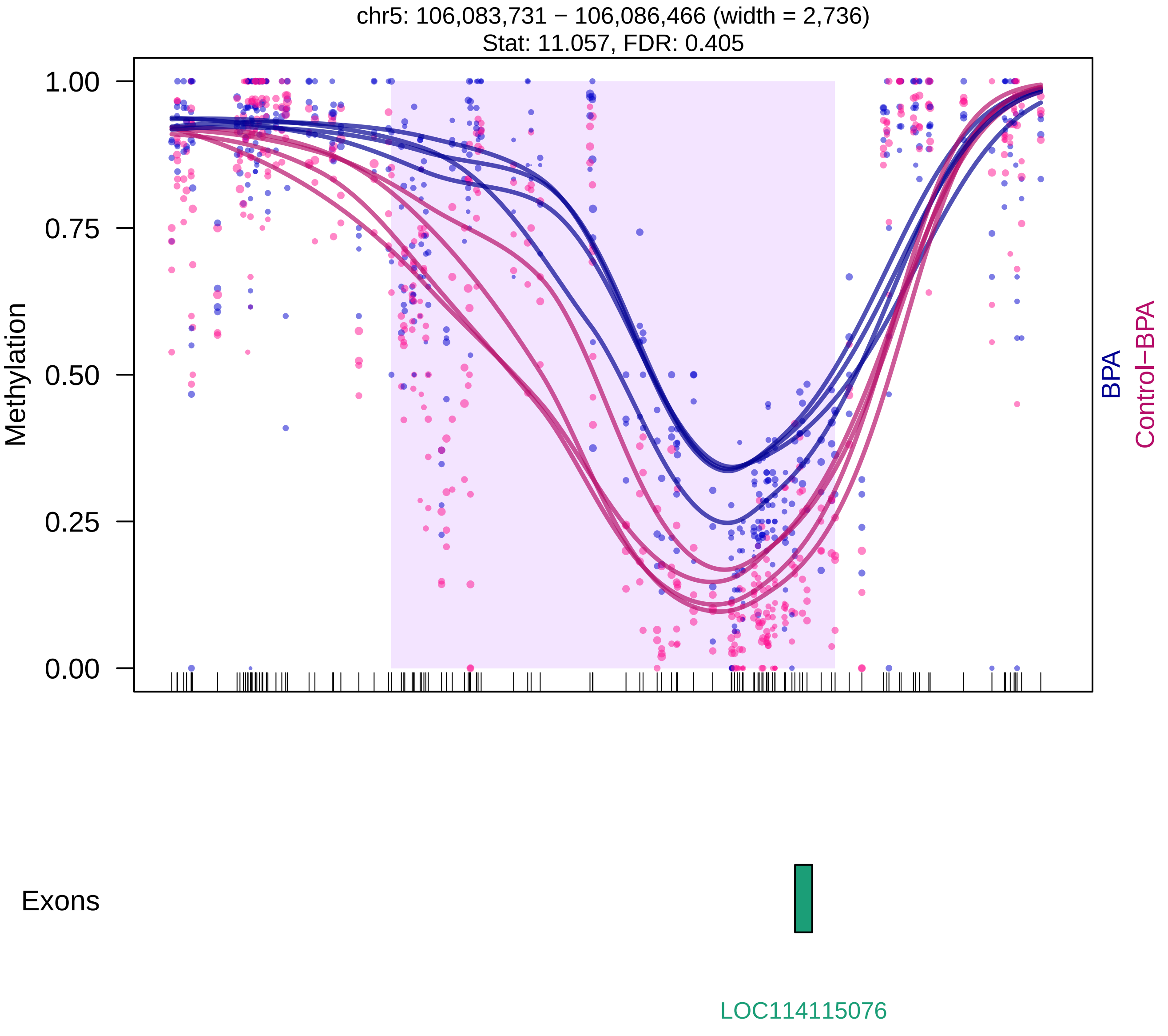
chr5: 106,083,731 − 106,086,466 (width = 2,736)
Stat: 11.057, FDR: 0.405
Methylation
1.00
0.75
0.50
0.25
0.00
BPA
Control−BPA
Exons
LOC114115076

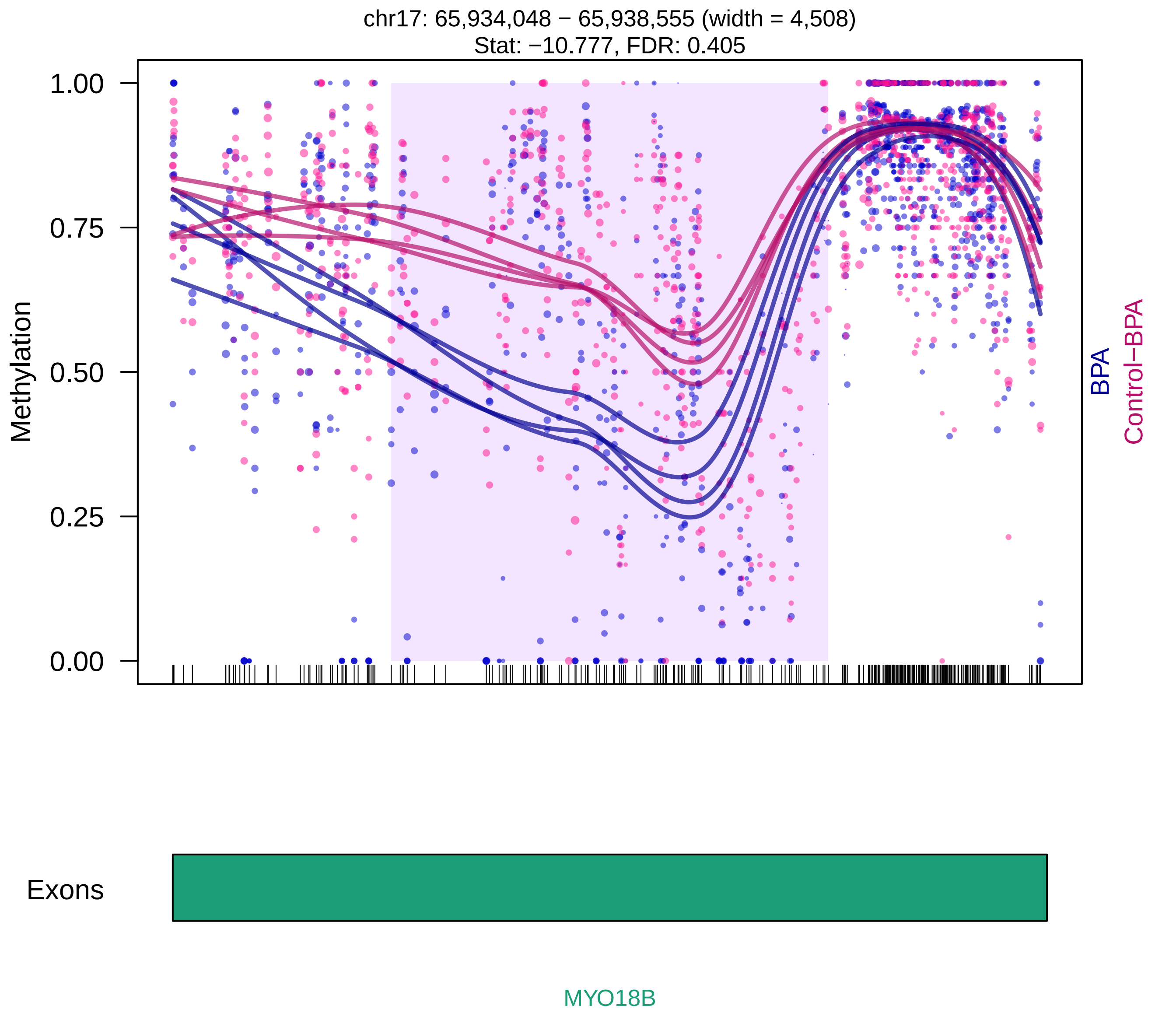

chr17: 65,934,048 − 65,938,555 (width = 4,508)
Stat: −10.777, FDR: 0.405
Methylation
1.00
0.75
0.50
0.25
0.00
BPA
Control−BPA
Exons
MYO18B

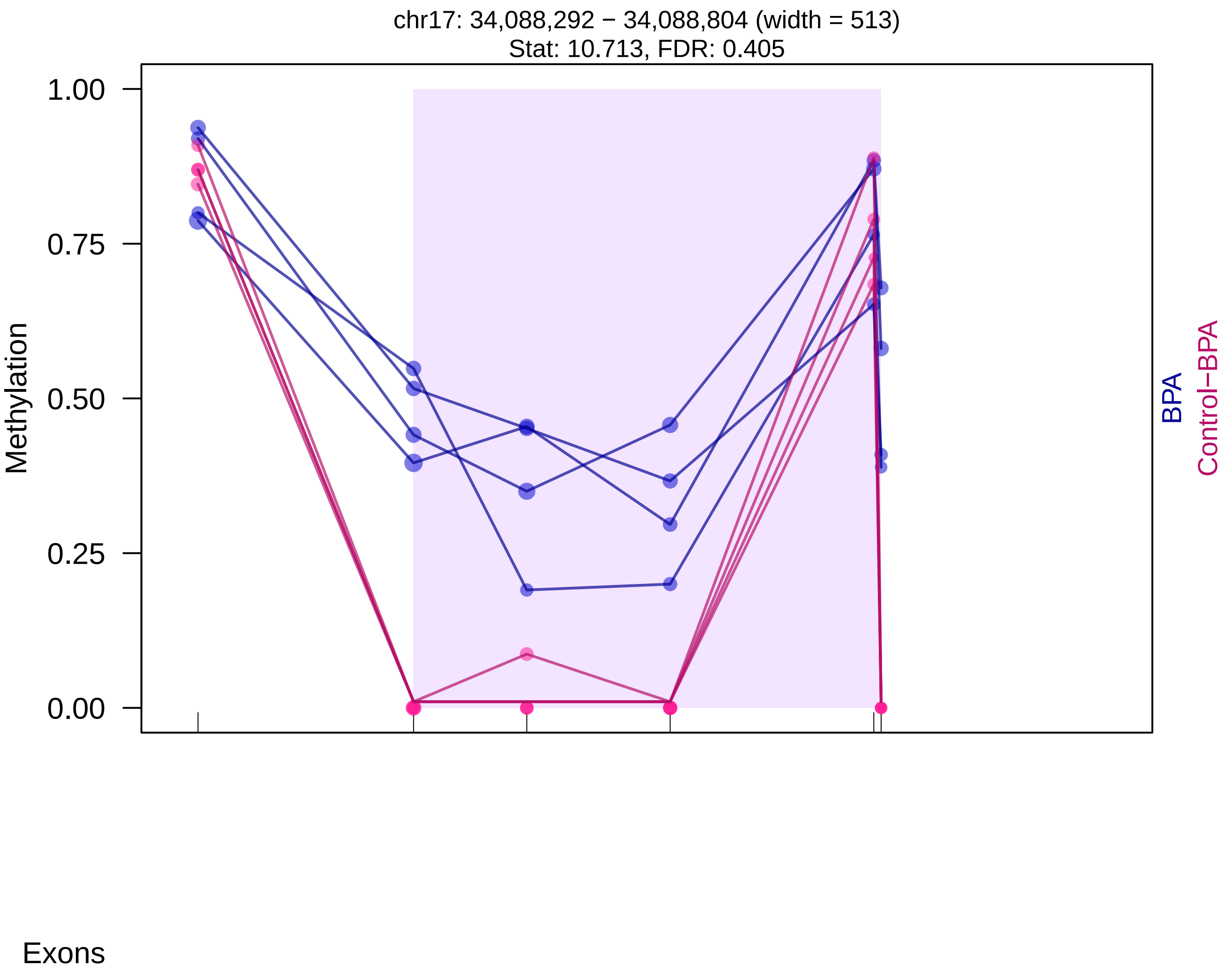

chr17: 34,088,292 − 34,088,804 (width = 513)
Stat: 10.713, FDR: 0.405
Methylation
1.00
0.75
0.50
0.25
0.00
BPA
Control−BPA
Exons

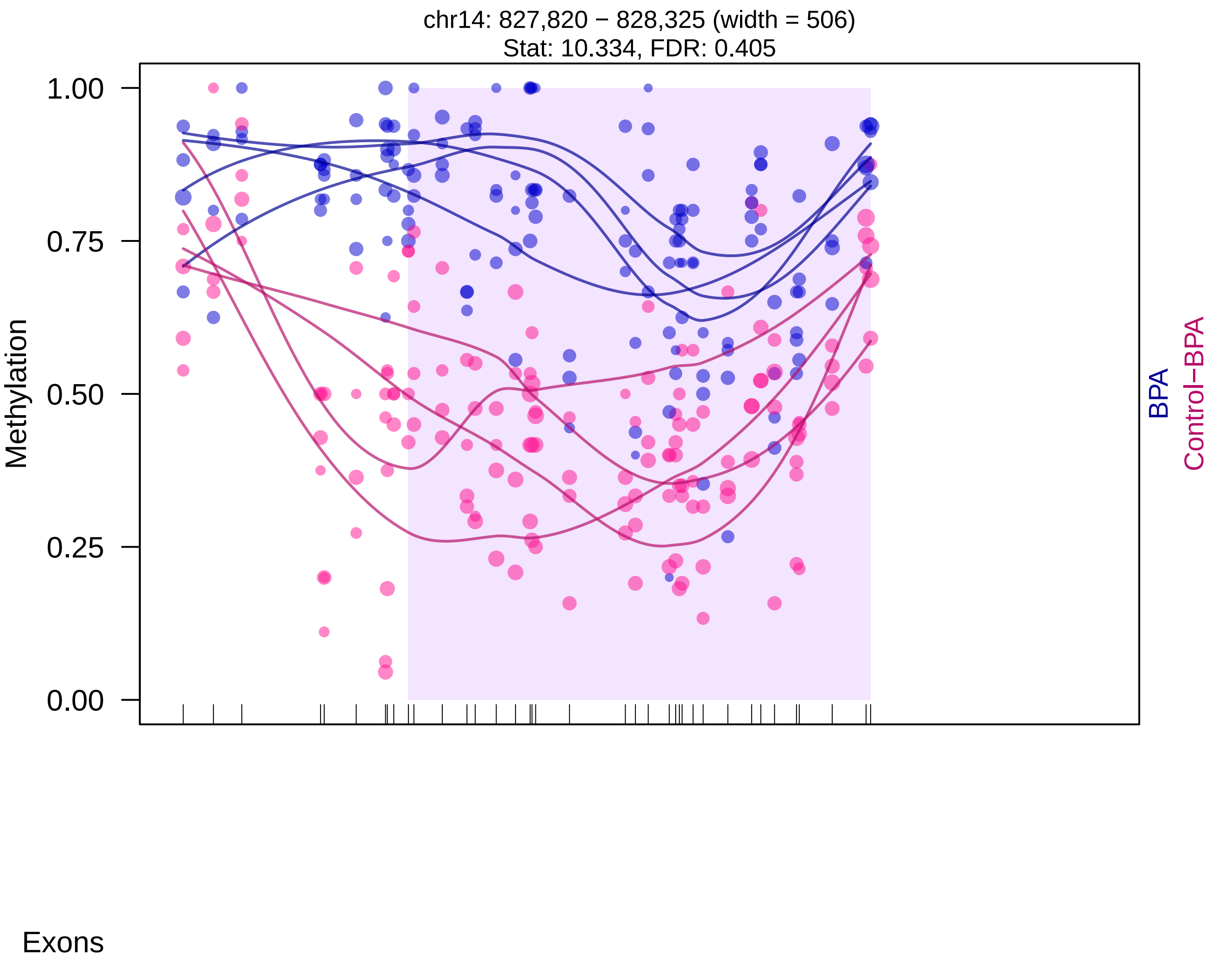
chr14: 827,820 − 828,325 (width = 506)
Stat: 10.334, FDR: 0.405
1.00
0.75
0.50
0.25
0.00
Methylation
BPA
Control−BPA
Exons

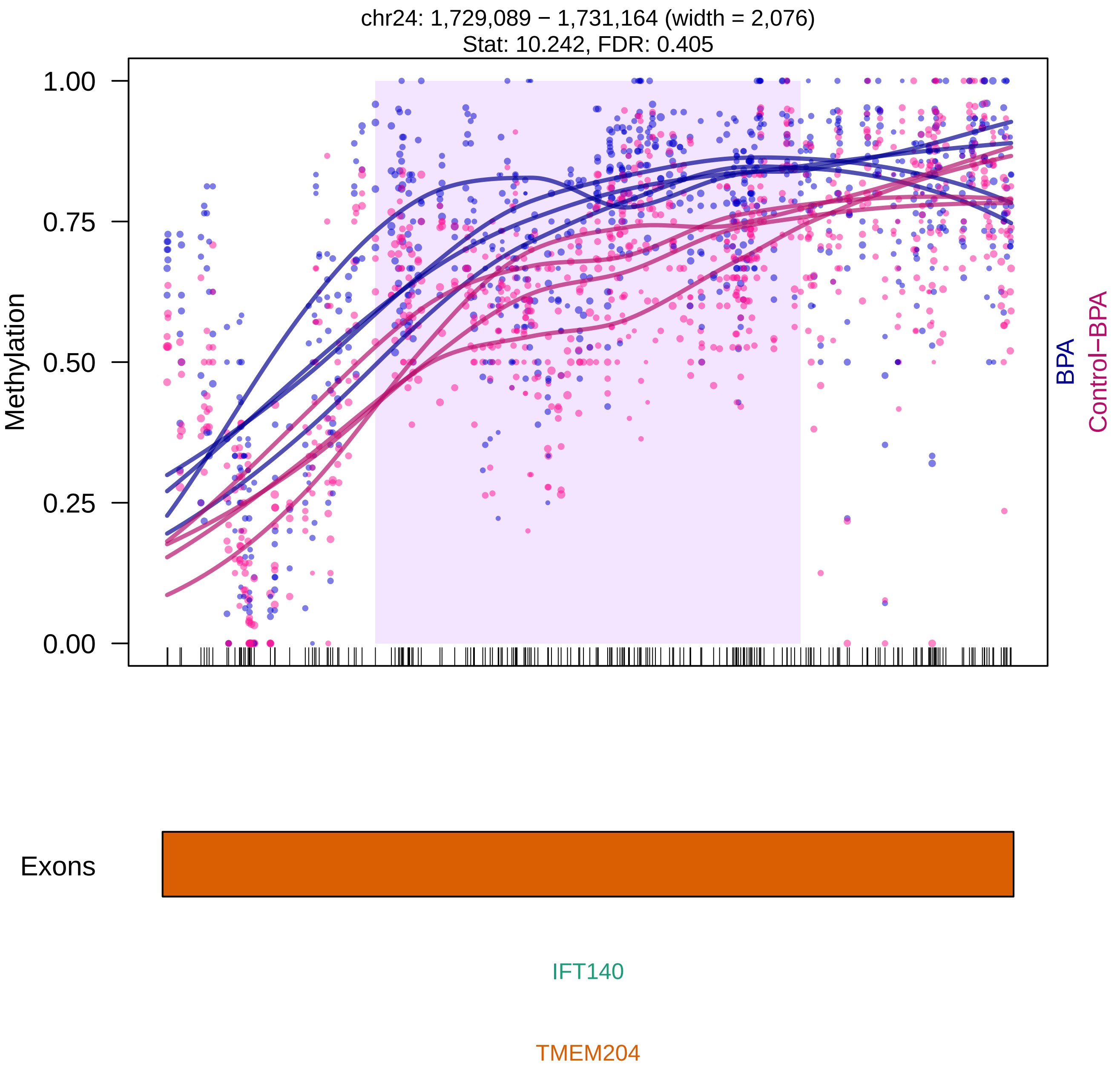

chr24: 1,729,089 − 1,731,164 (width = 2,076)
Stat: 10.242, FDR: 0.405
Methylation
1.00
0.75
0.50
0.25
0.00
BPA
Control−BPA
Exons
IFT140
TMEM204

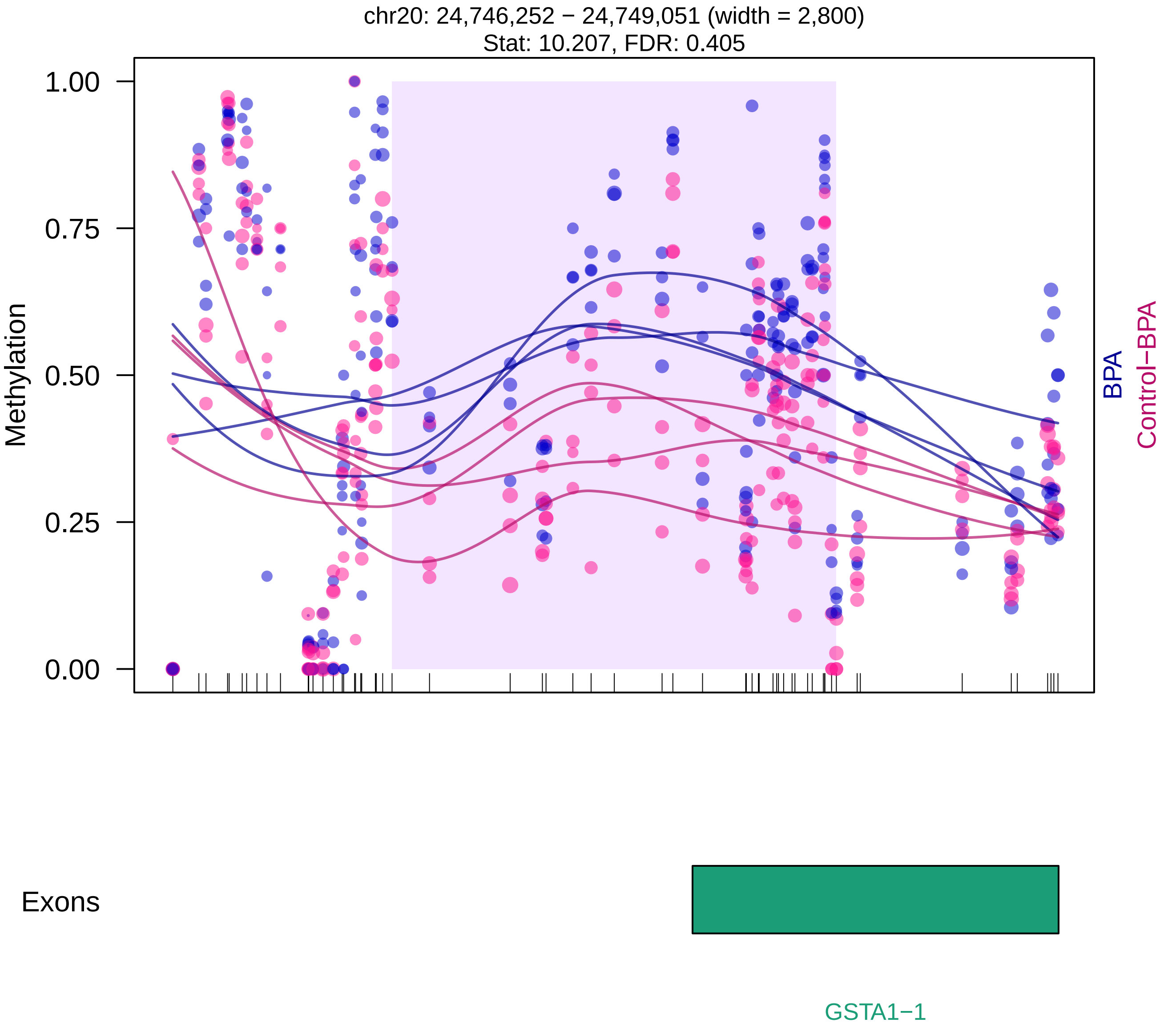
chr20: 24,746,252 − 24,749,051 (width = 2,800)
Stat: 10.207, FDR: 0.405
Methylation
1.00
0.75
0.50
0.25
0.00
BPA
Control−BPA
Exons
GSTA1−1

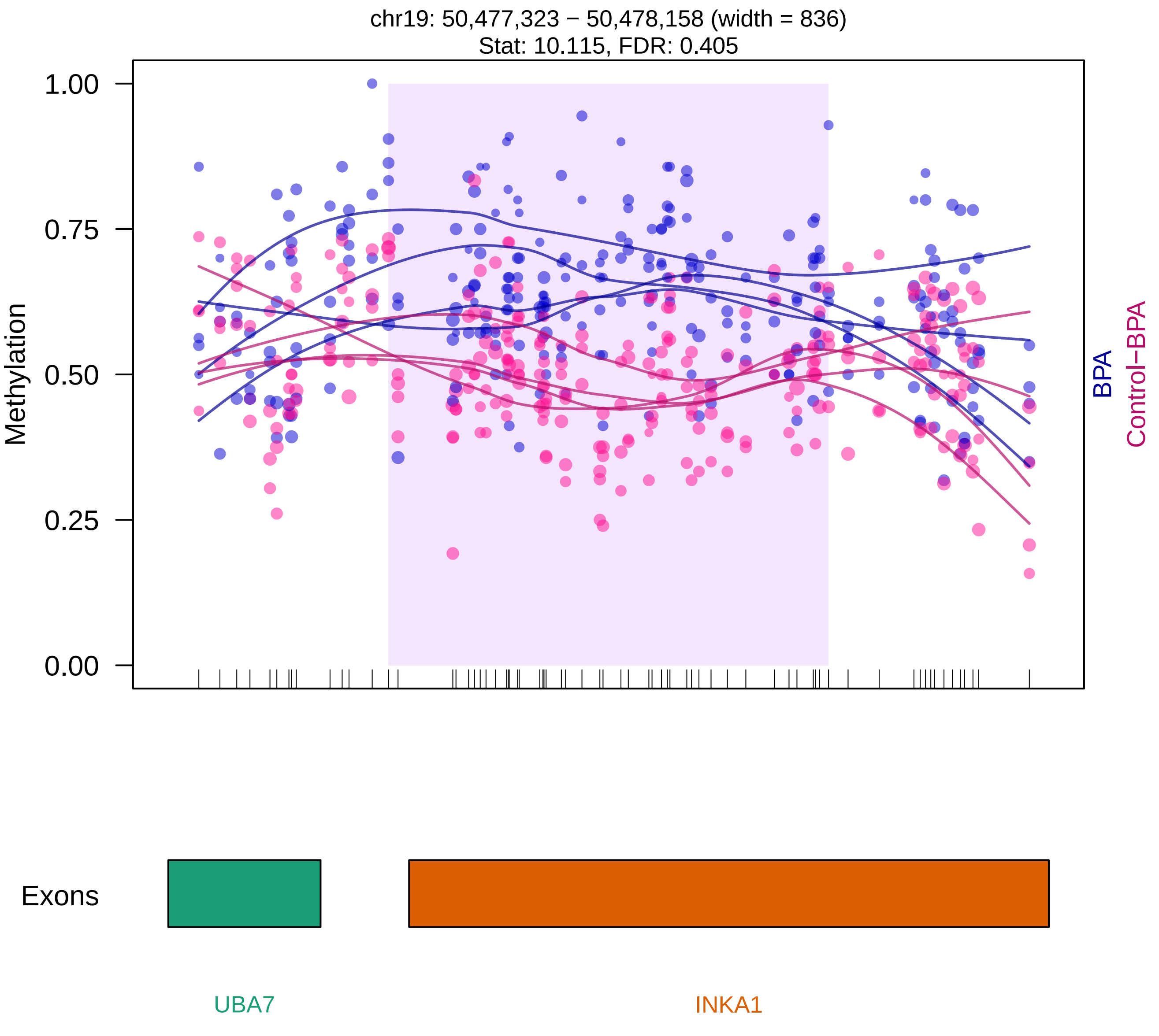

chr19: 50,477,323 − 50,478,158 (width = 836)
Stat: 10.115, FDR: 0.405
Methylation
1.00
0.75
0.50
0.25
0.00
BPA
Control−BPA
Exons
UBA7
INKA1

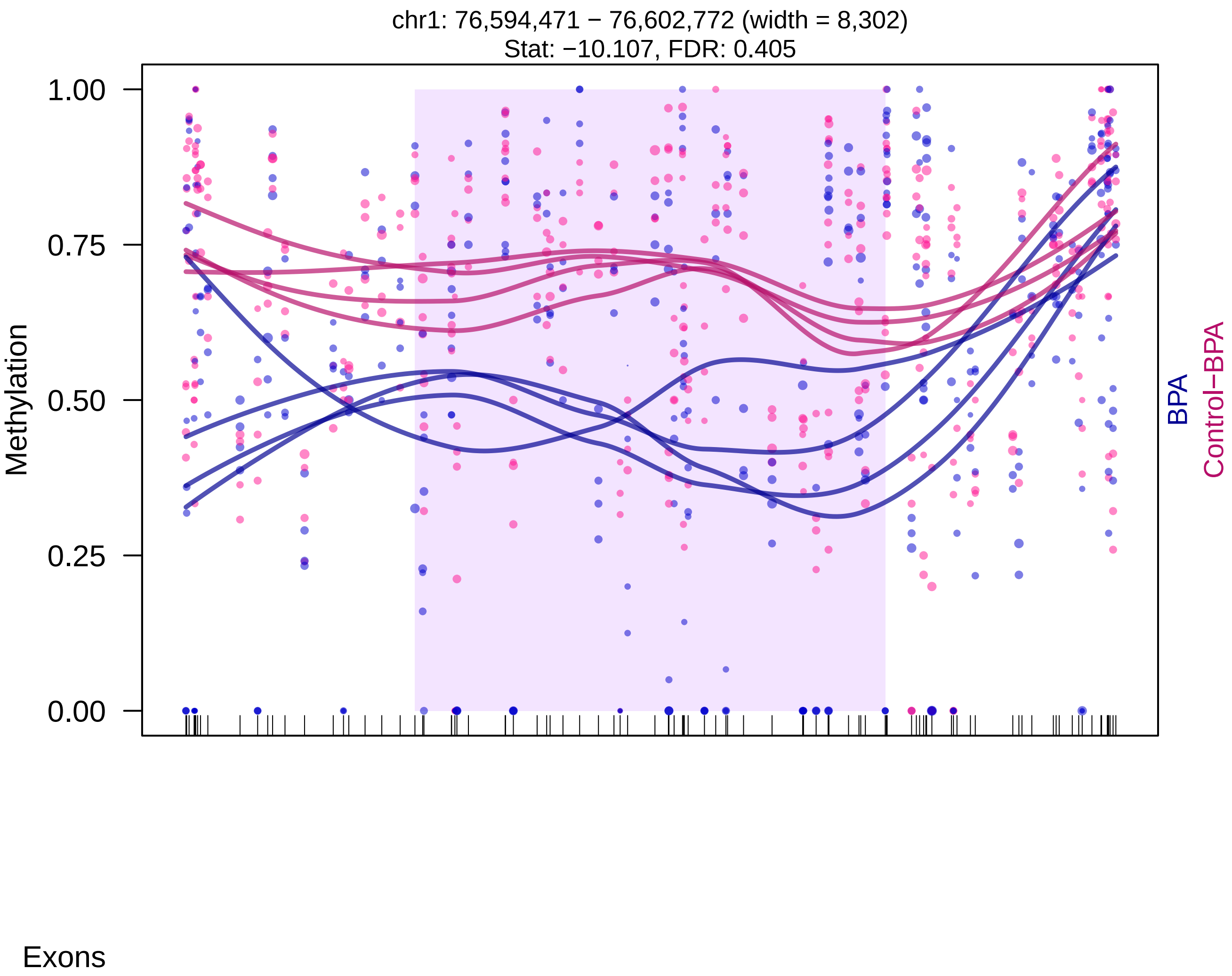
chr1: 76,594,471 − 76,602,772 (width = 8,302)
Stat: −10.107, FDR: 0.405
Methylation
1.00
0.75
0.50
0.25
0.00
BPA
Control−BPA
Exons

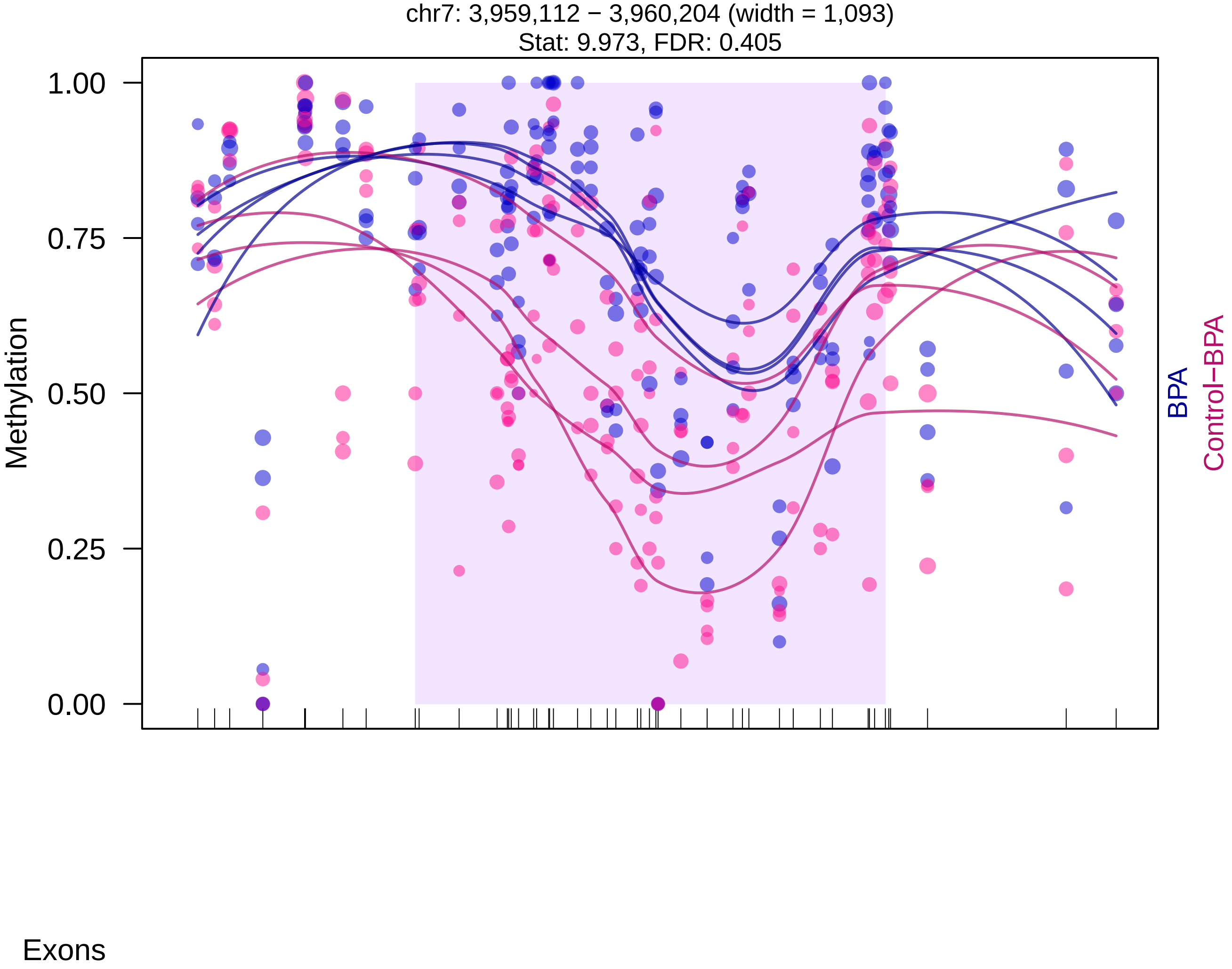

chr7: 3,959,112 − 3,960,204 (width = 1,093)
Stat: 9.973, FDR: 0.405
Methylation
1.00
0.75
0.50
0.25
0.00
BPA
Control−BPA
Exons

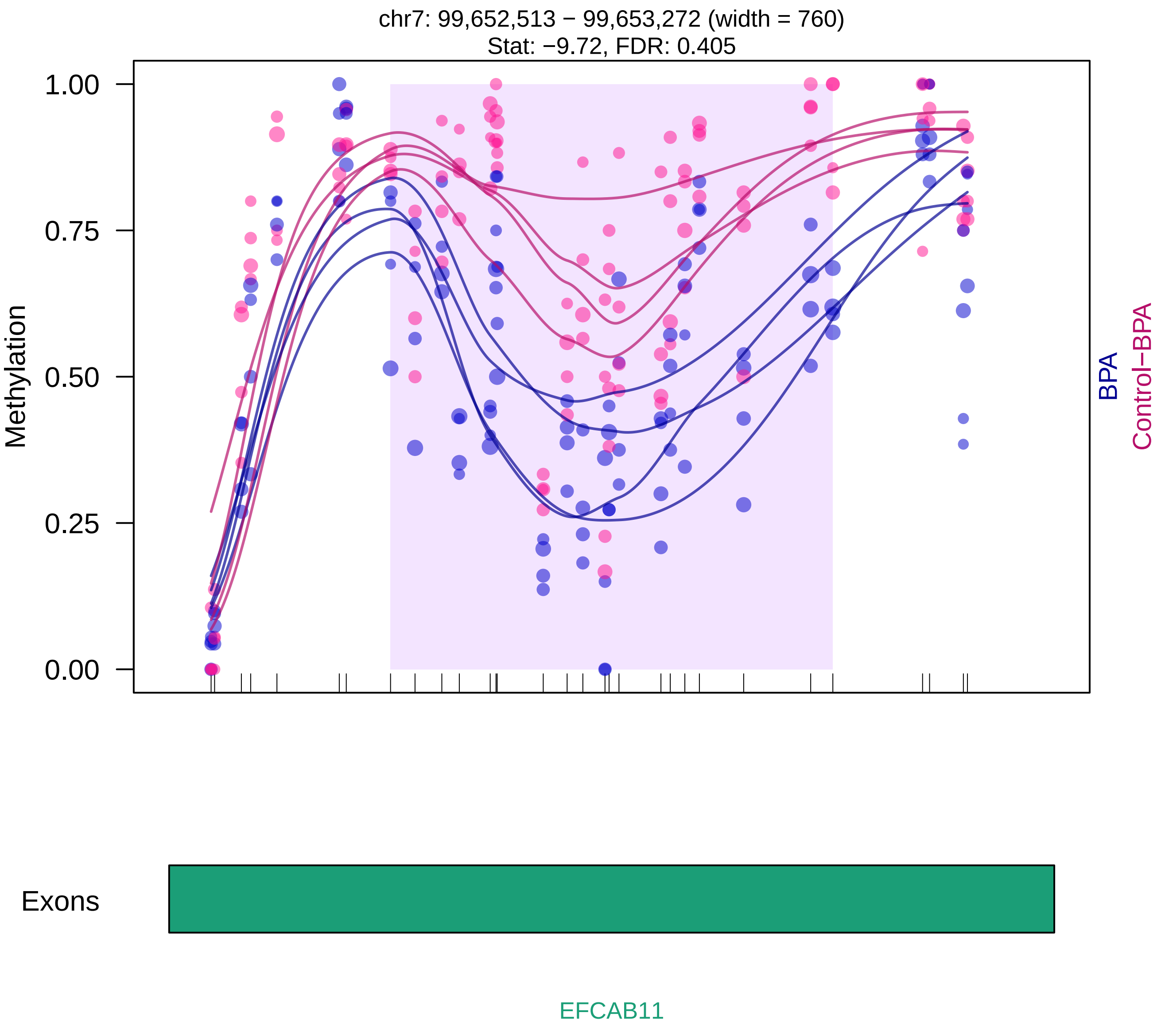

chr7: 99,652,513 − 99,653,272 (width = 760)
Stat: −9.72, FDR: 0.405
1.00
0.75
0.50
0.25
0.00
Methylation
BPA
Control−BPA
Exons
EFCAB11

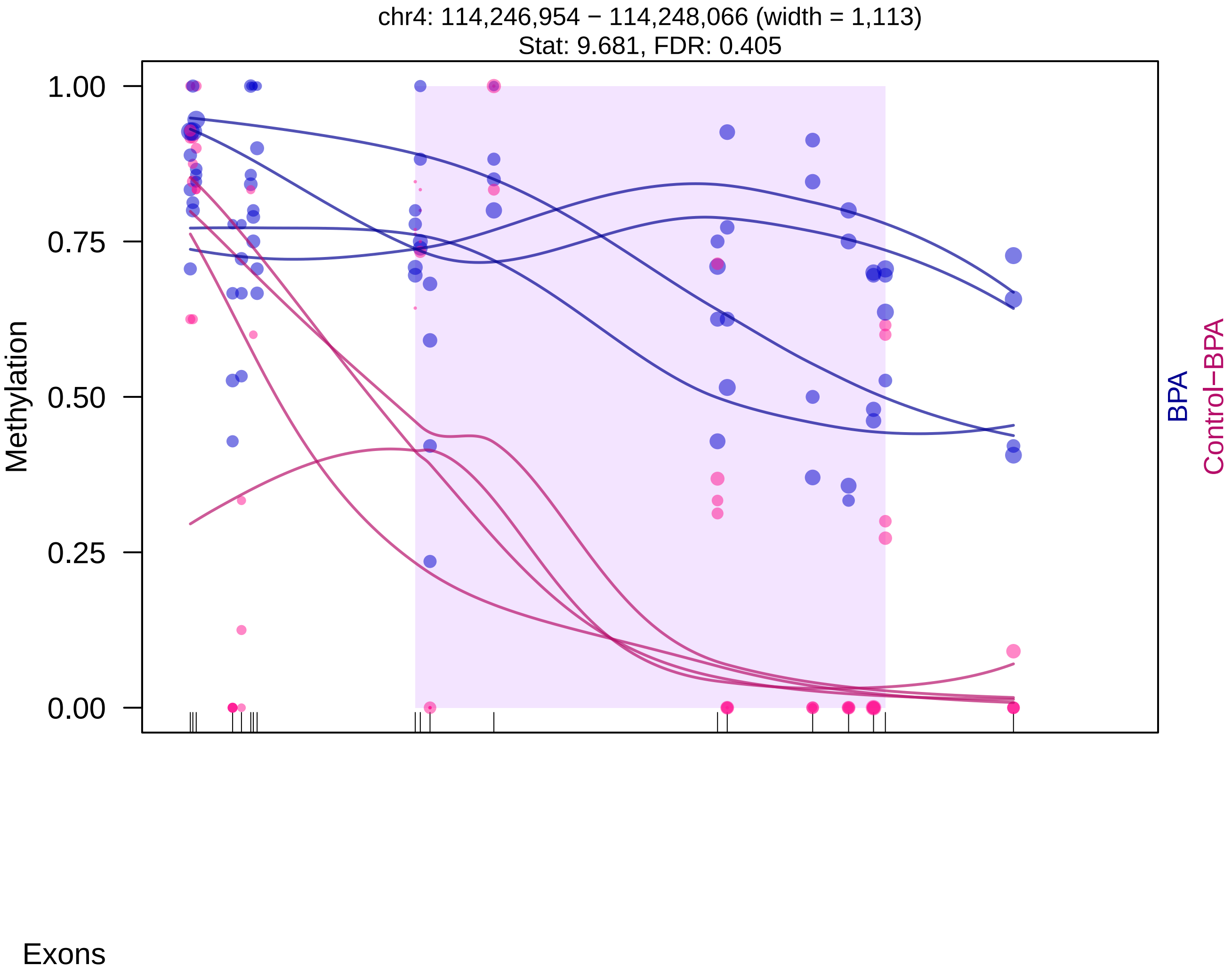
chr4: 114,246,954 − 114,248,066 (width = 1,113)
Stat: 9.681, FDR: 0.405
1.00
0.75
0.50
0.25
0.00
Methylation
BPA
Control−BPA
Exons

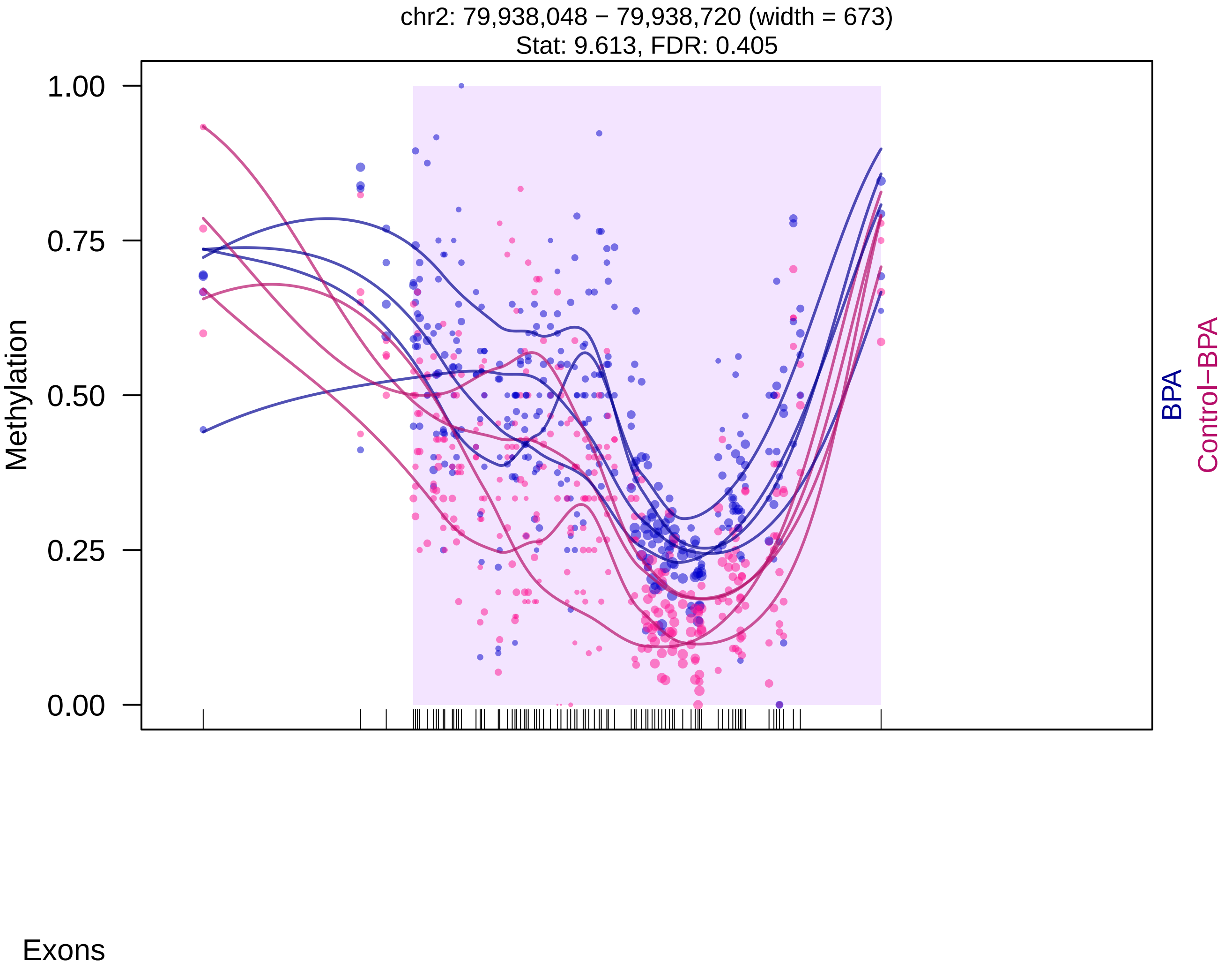

chr2: 79,938,048 − 79,938,720 (width = 673)
Stat: 9.613, FDR: 0.405
Methylation
1.00
0.75
0.50
0.25
0.00
BPA
Control−BPA
Exons

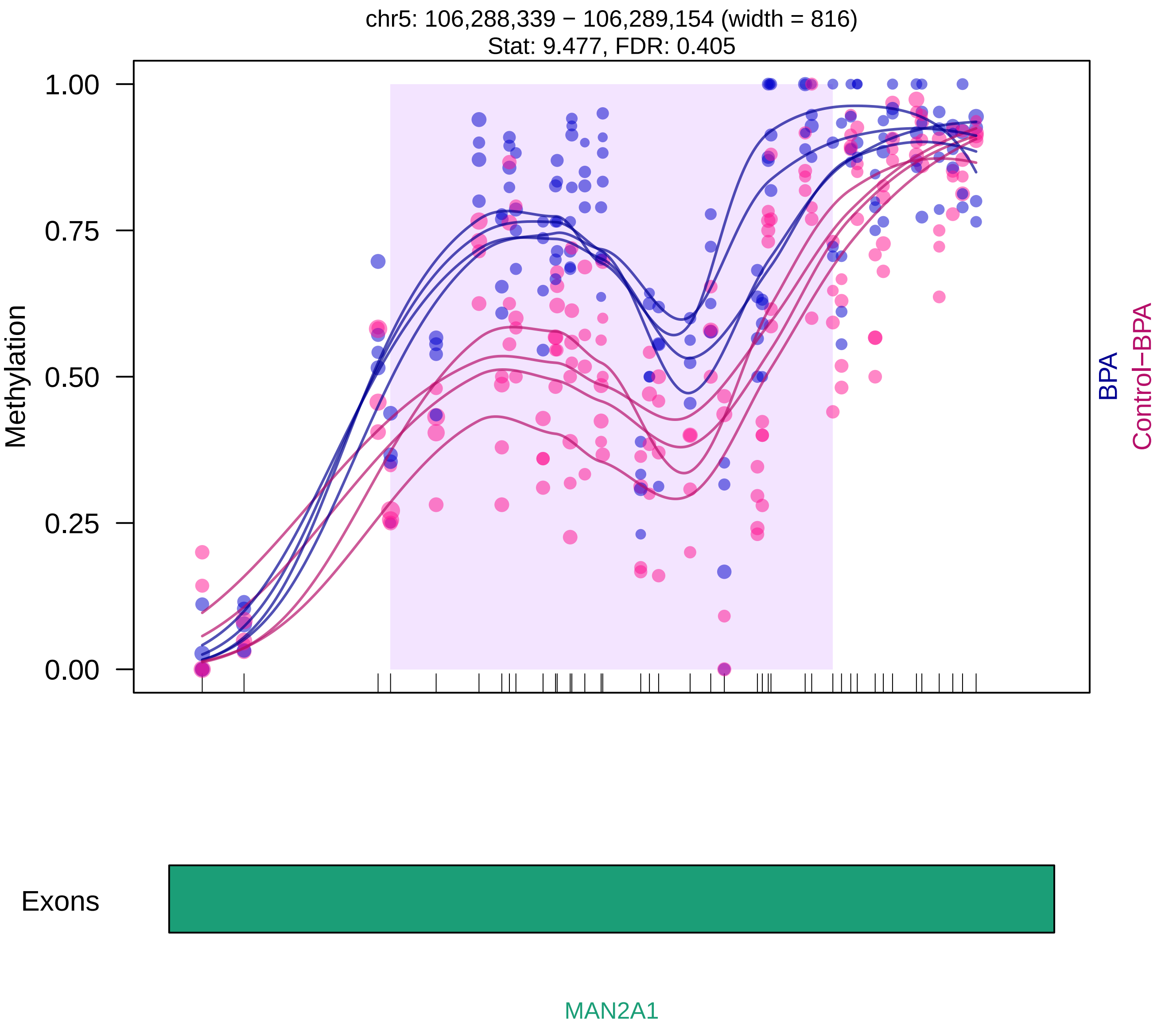

chr5: 106,288,339 − 106,289,154 (width = 816)
Stat: 9.477, FDR: 0.405
Methylation
1.00
0.75
0.50
0.25
0.00
BPA
Control−BPA
Exons
MAN2A1

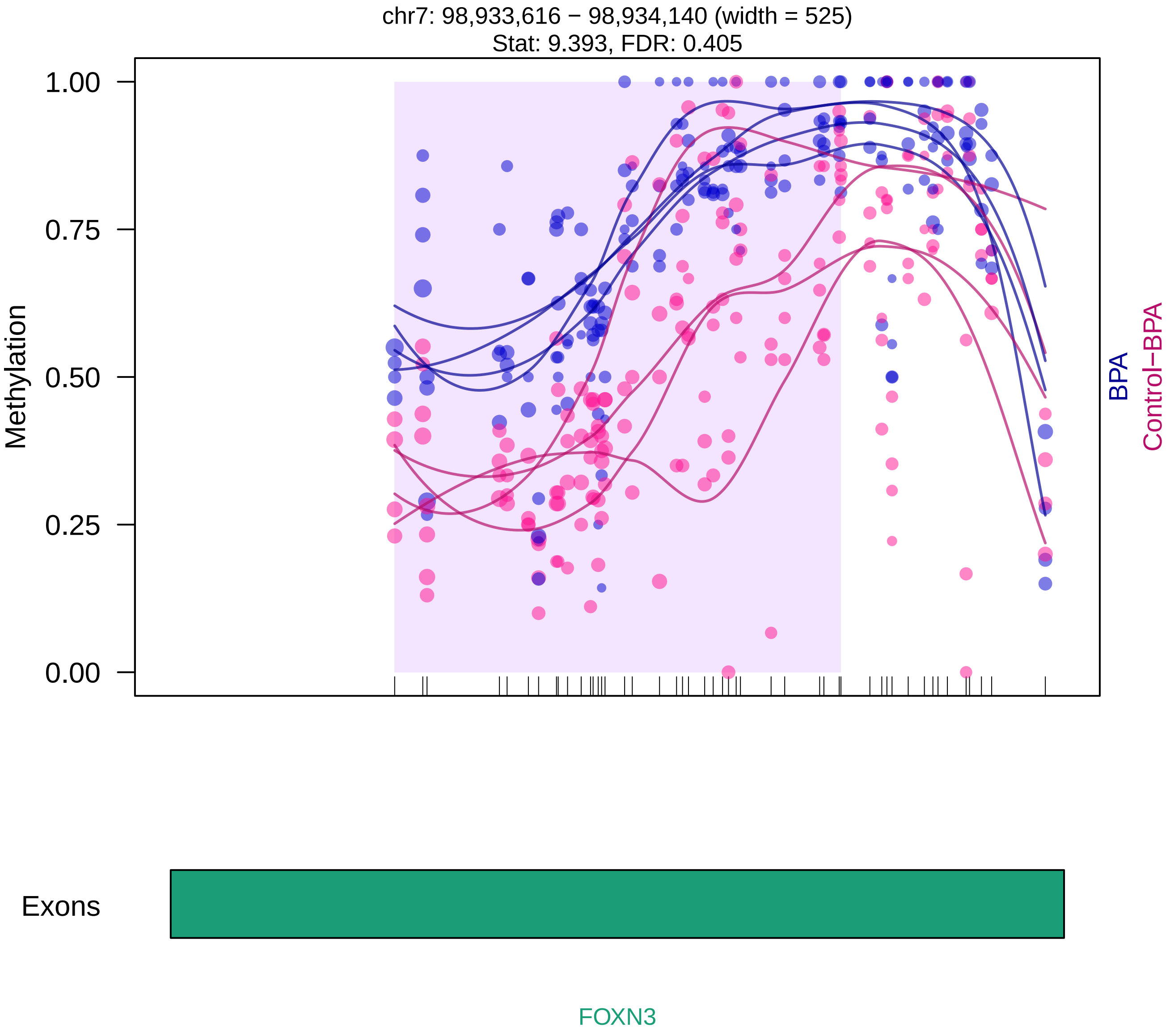
chr7: 98,933,616 − 98,934,140 (width = 525)
Stat: 9.393, FDR: 0.405
Methylation
1.00
0.75
0.50
0.25
0.00
BPA
Control−BPA
Exons
FOXN3

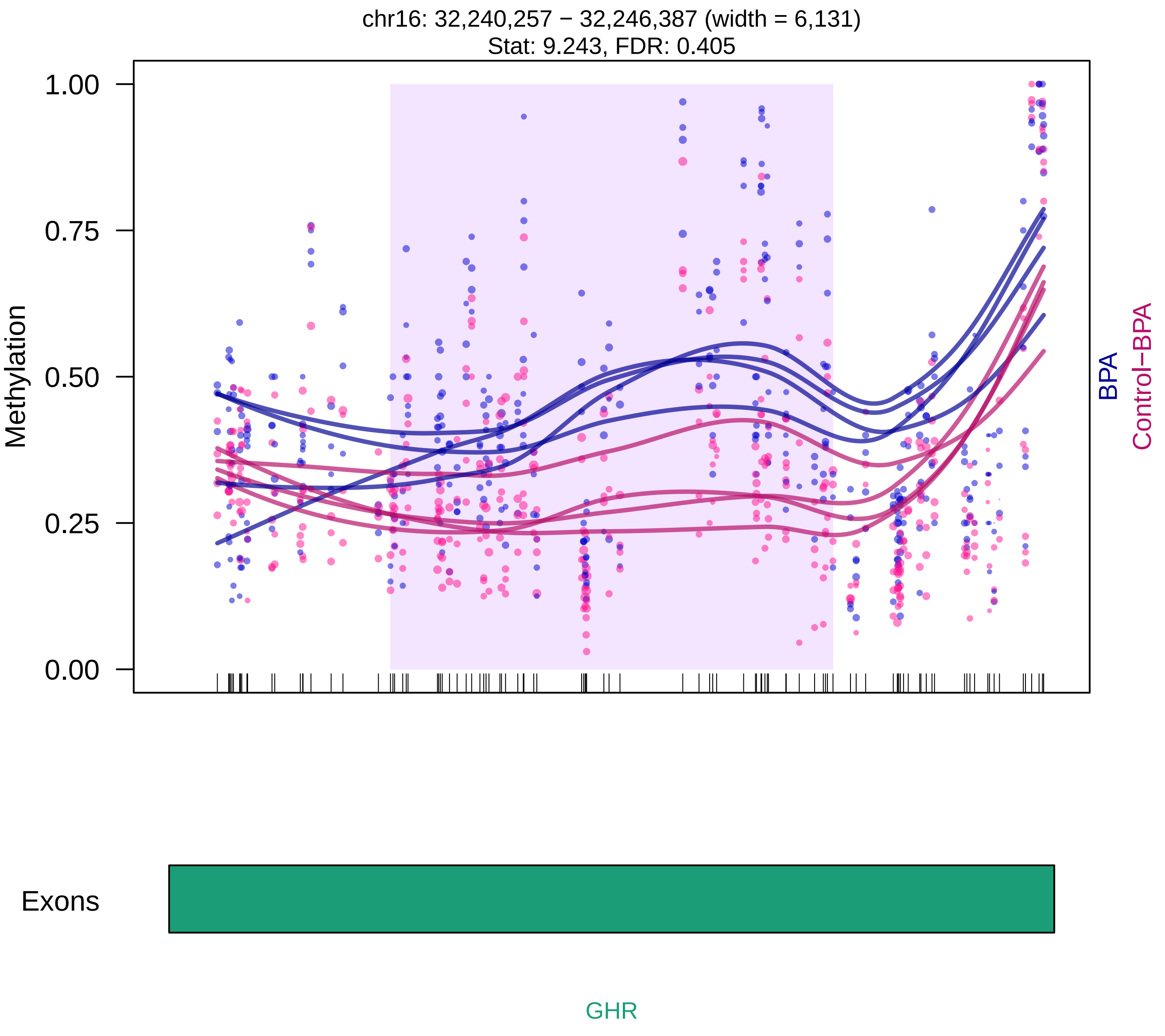

chr16: 32,240,257 − 32,246,387 (width = 6,131)
Stat: 9.243, FDR: 0.405
Methylation
1.00
0.75
0.50
0.25
0.00
BPA
Control−BPA
Exons
GHR

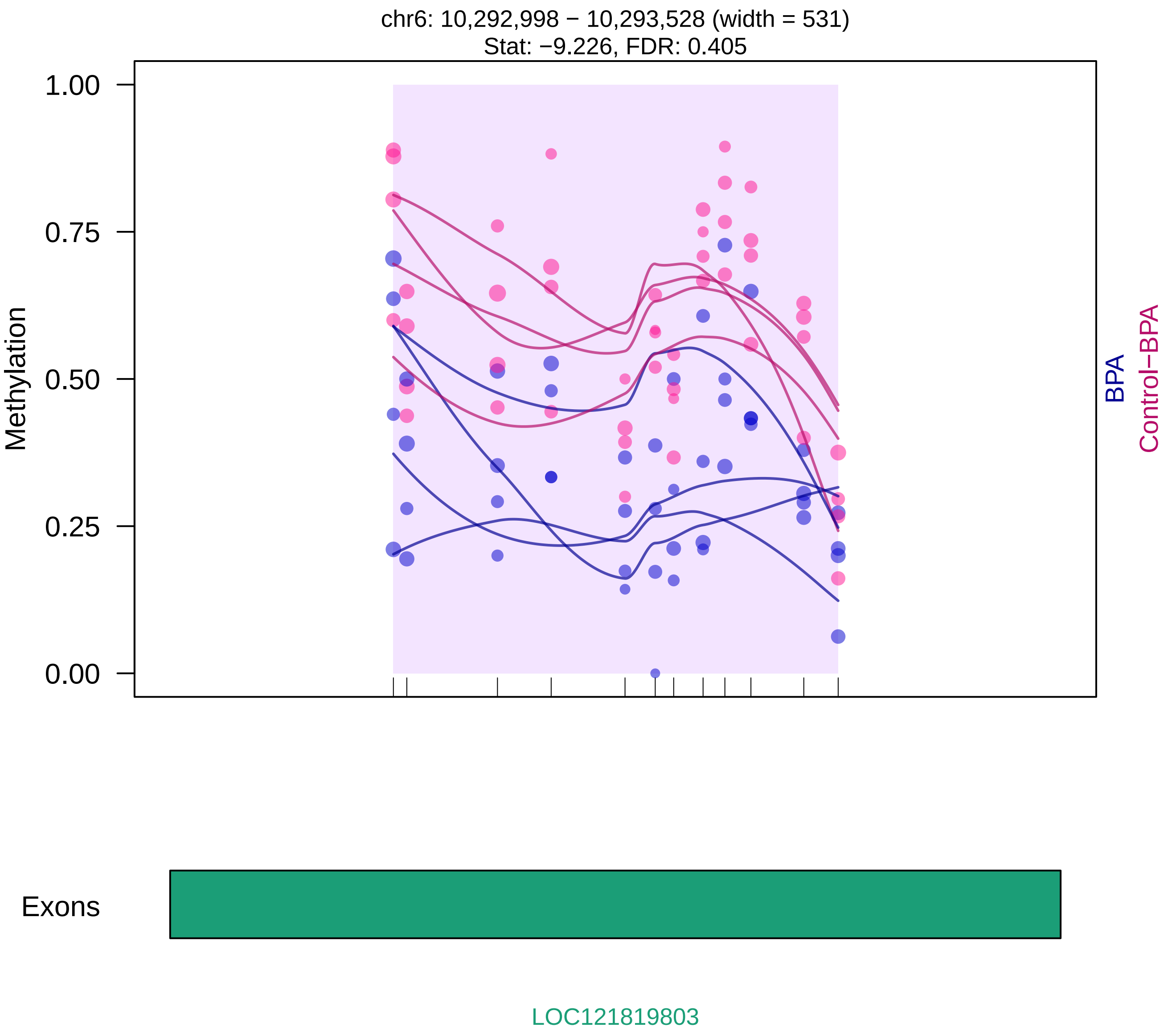
chr6: 10,292,998 − 10,293,528 (width = 531)
Stat: −9.226, FDR: 0.405
Methylation
1.00
0.75
0.50
0.25
0.00
BPA
Control−BPA
Exons
LOC121819803

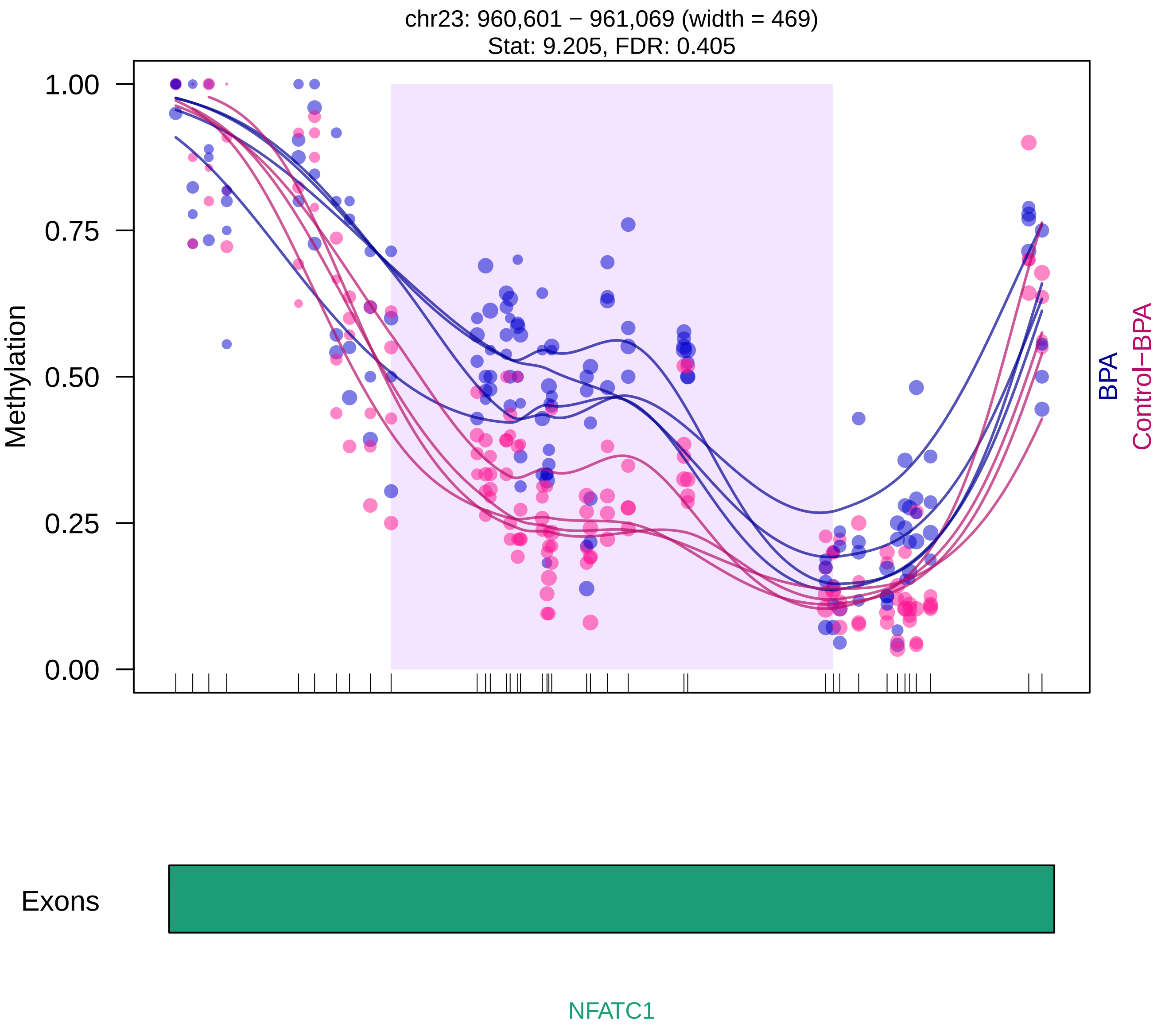
chr23: 960,601 − 961,069 (width = 469)
Stat: 9.205, FDR: 0.405
Methylation
1.00
0.75
0.50
0.25
0.00
BPA
Control−BPA
Exons
NFATC1

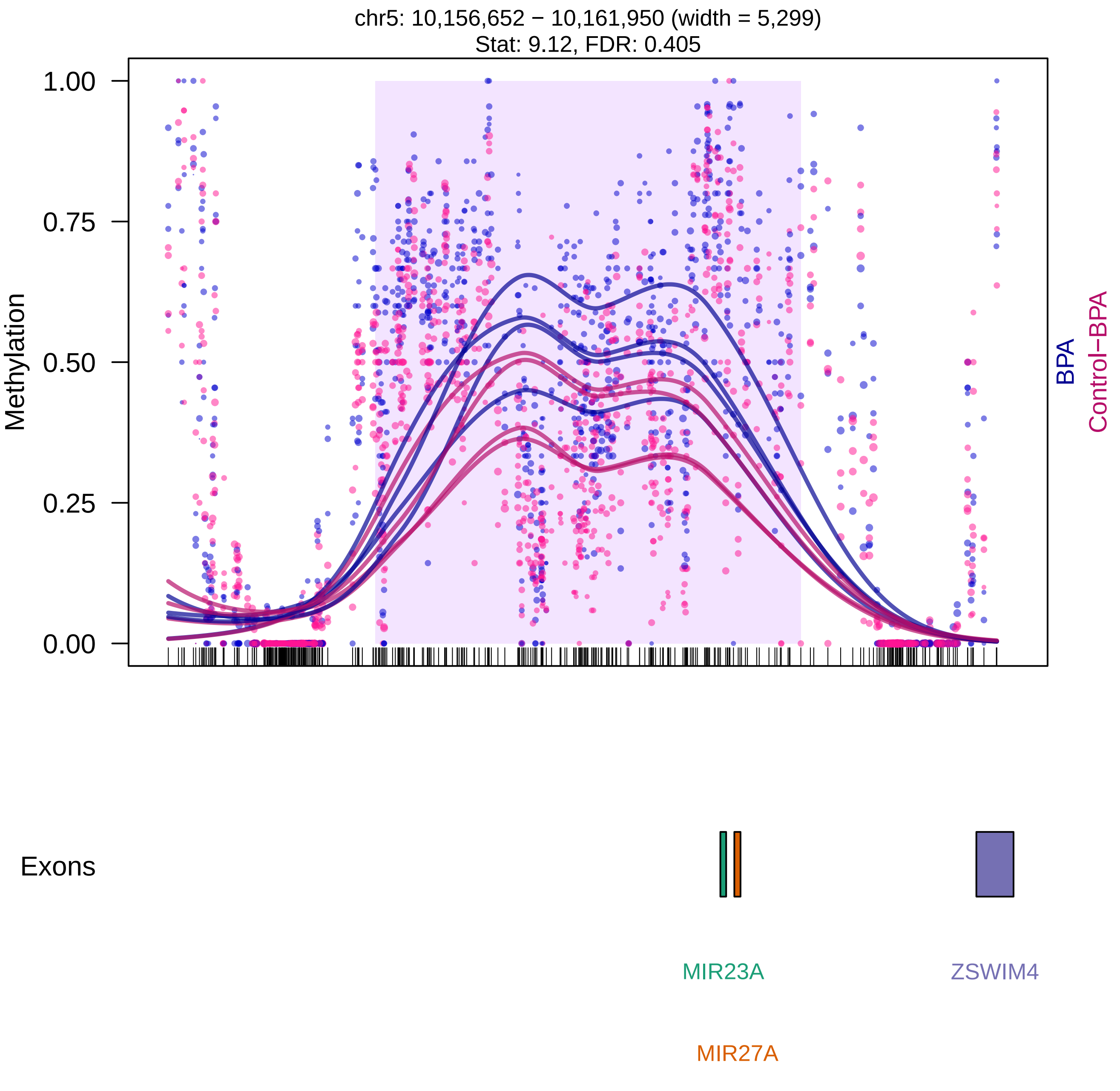
chr5: 10,156,652 − 10,161,950 (width = 5,299)
Stat: 9.12, FDR: 0.405
1.00
0.75
0.50
0.25
0.00
Methylation
BPA
Control−BPA
Exons
MIR23A
MIR27A
ZSWIM4

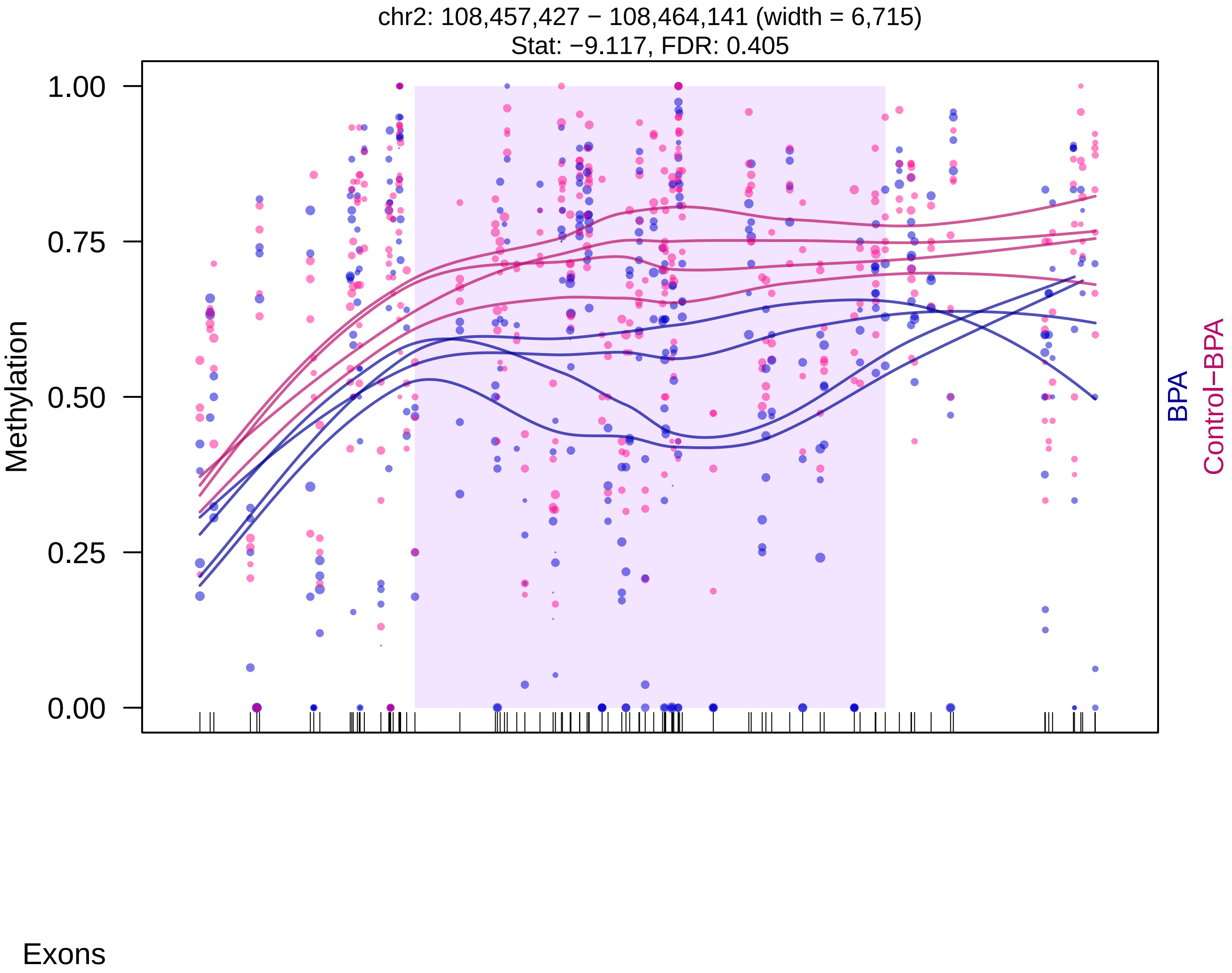
chr2: 108,457,427 − 108,464,141 (width = 6,715)
Stat: −9.117, FDR: 0.405
1.00
0.75
0.50
0.25
0.00
Methylation
BPA
Control−BPA
Exons

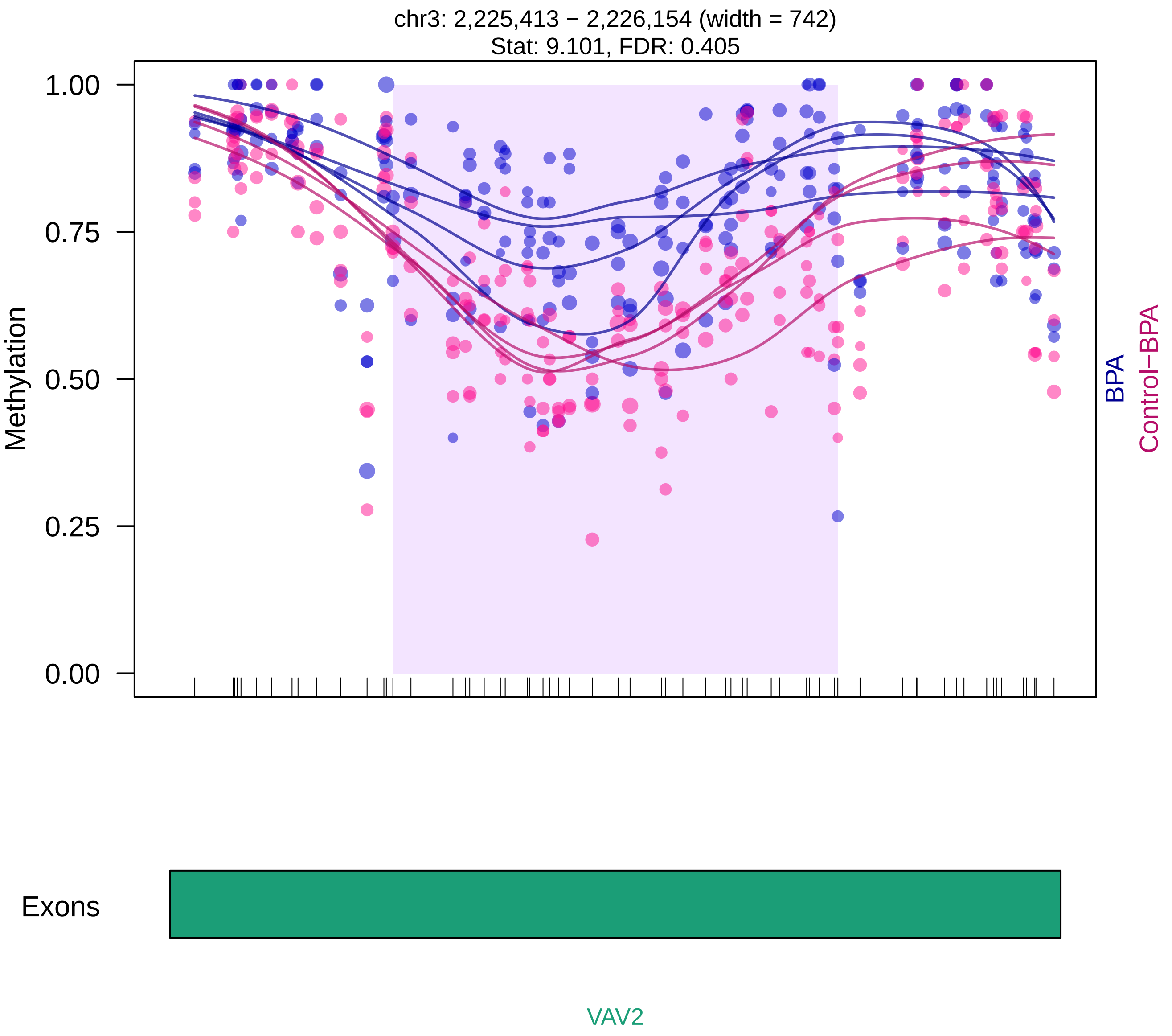

chr3: 2,225,413 − 2,226,154 (width = 742)
Stat: 9.101, FDR: 0.405
Methylation
1.00
0.75
0.50
0.25
0.00
BPA
Control−BPA
Exons
VAV2

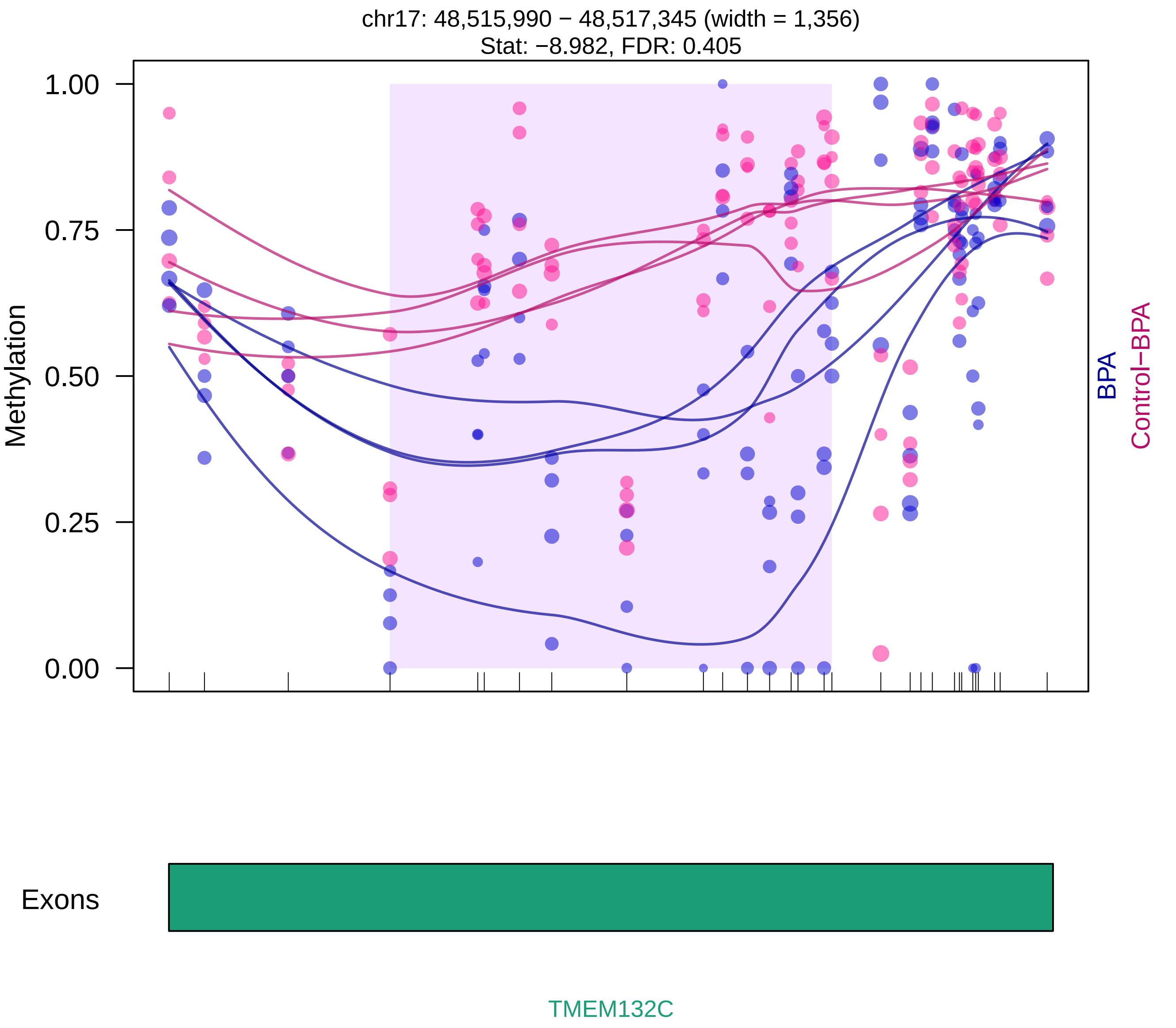
chr17: 48,515,990 − 48,517,345 (width = 1,356)
Stat: −8.982, FDR: 0.405
Methylation
1.00
0.75
0.50
0.25
0.00
BPA
Control−BPA
Exons
TMEM132C

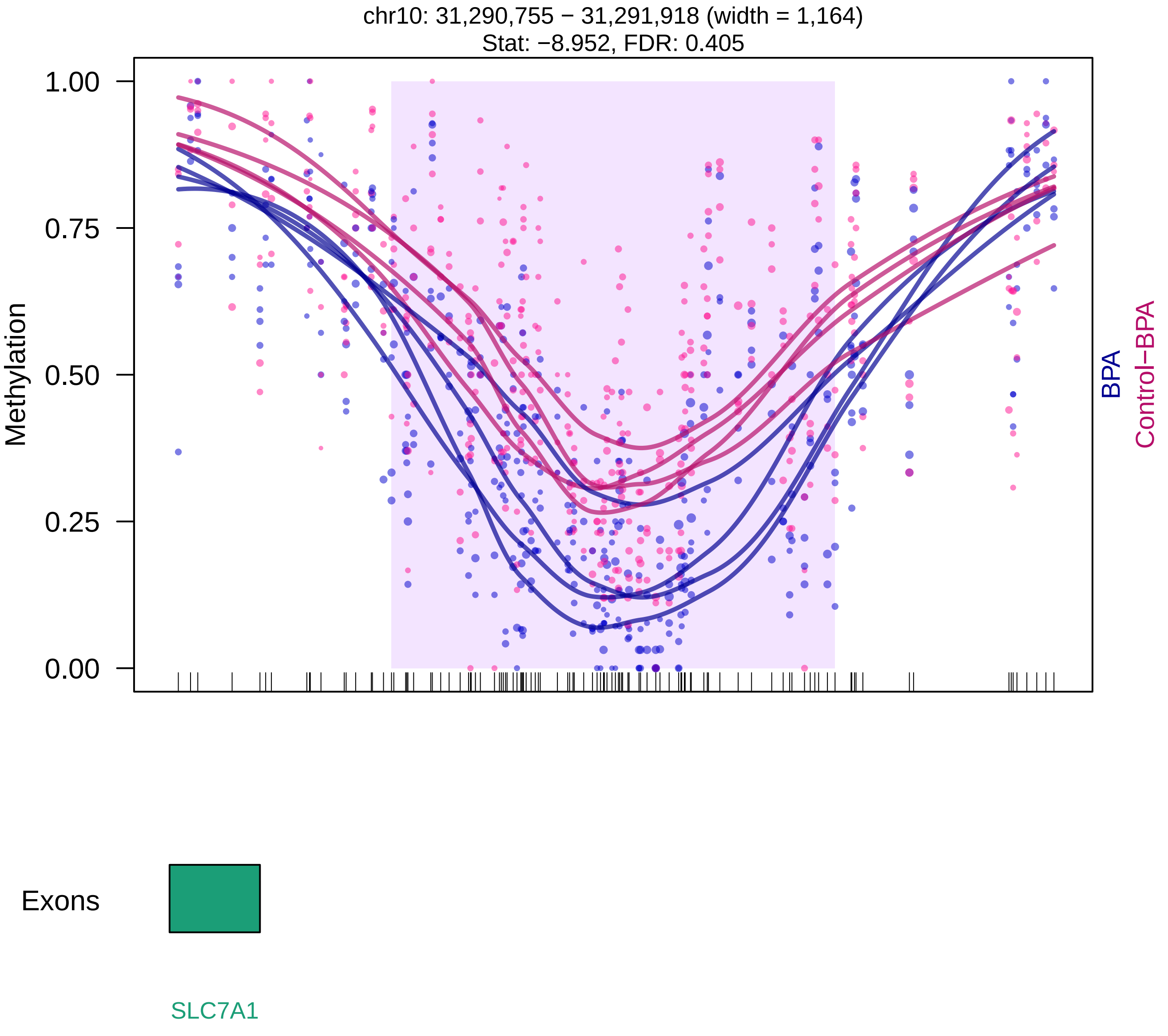
chr10: 31,290,755 − 31,291,918 (width = 1,164)
Stat: −8.952, FDR: 0.405
Methylation
1.00
0.75
0.50
0.25
0.00
BPA
Control−BPA
Exons
SLC7A1

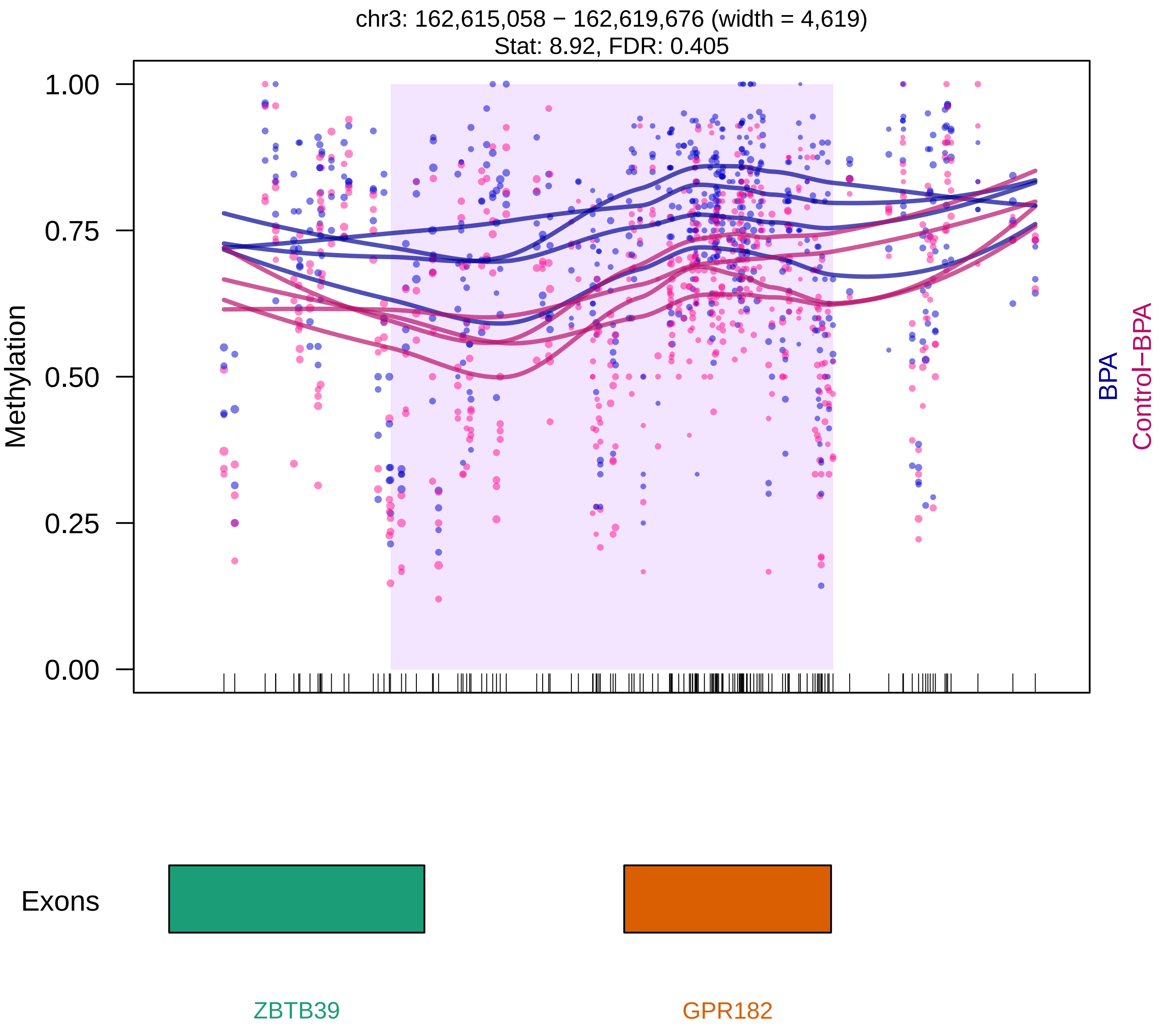
chr3: 162,615,058 − 162,619,676 (width = 4,619)
Stat: 8.92, FDR: 0.405
Methylation
1.00
0.75
0.50
0.25
0.00
BPA
Control−BPA
Exons
ZBTB39
GPR182

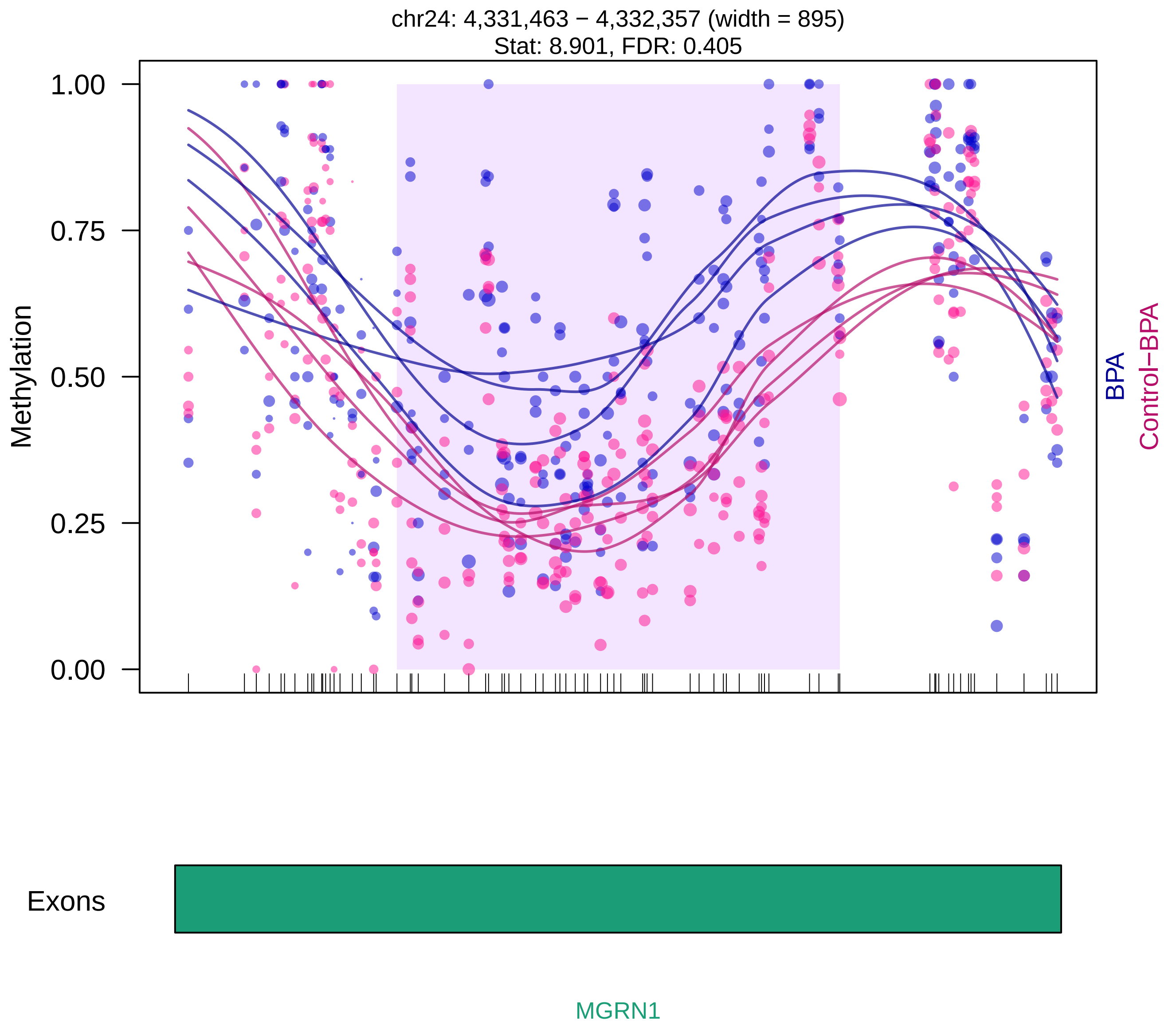
chr24: 4,331,463 − 4,332,357 (width = 895)
Stat: 8.901, FDR: 0.405
Methylation
1.00
0.75
0.50
0.25
0.00
BPA
Control−BPA
Exons
MGRN1

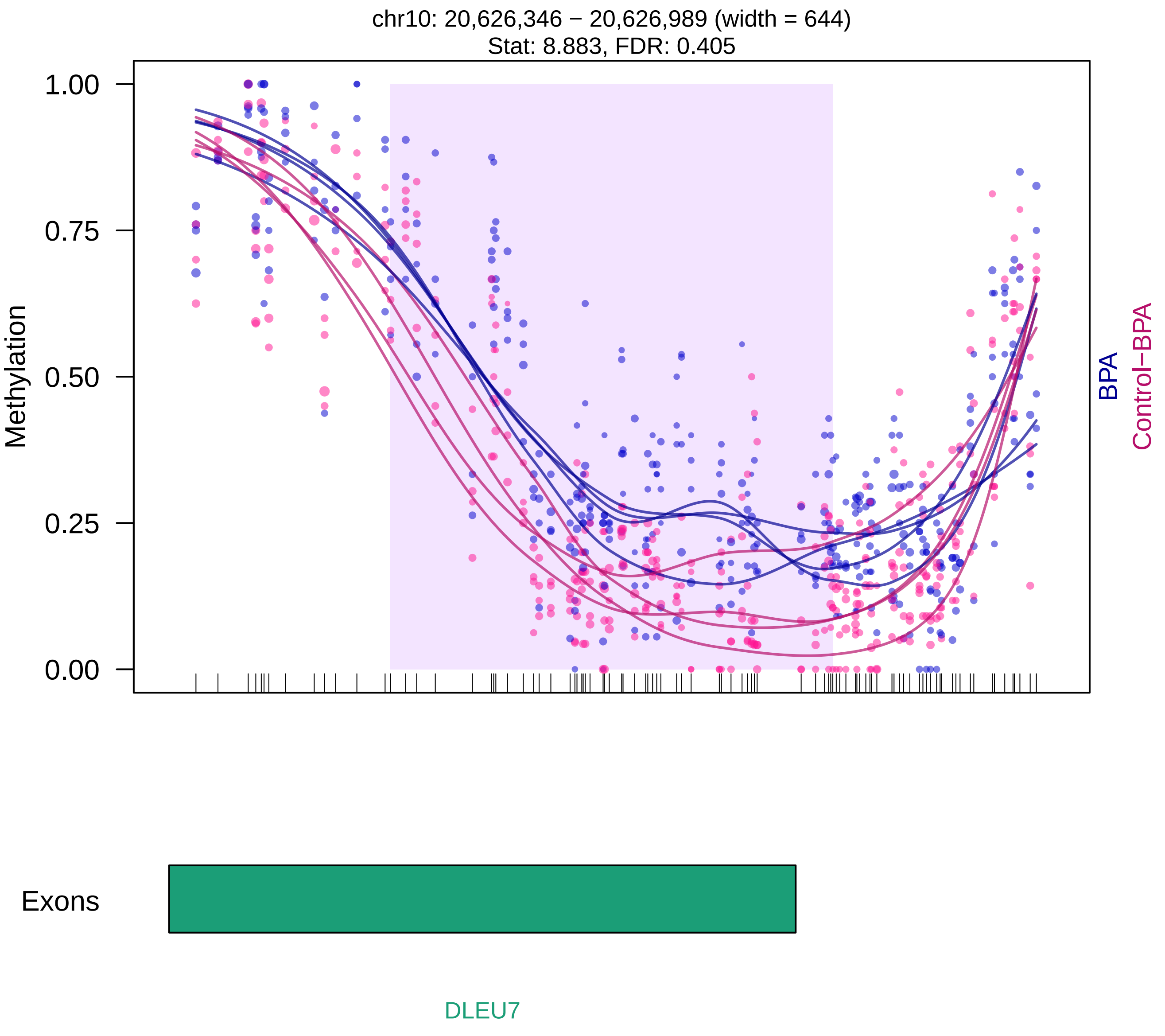

chr10: 20,626,346 − 20,626,989 (width = 644)
Stat: 8.883, FDR: 0.405
1.00
0.75
0.50
0.25
0.00
Methylation
BPA
Control−BPA
Exons
DLEU7

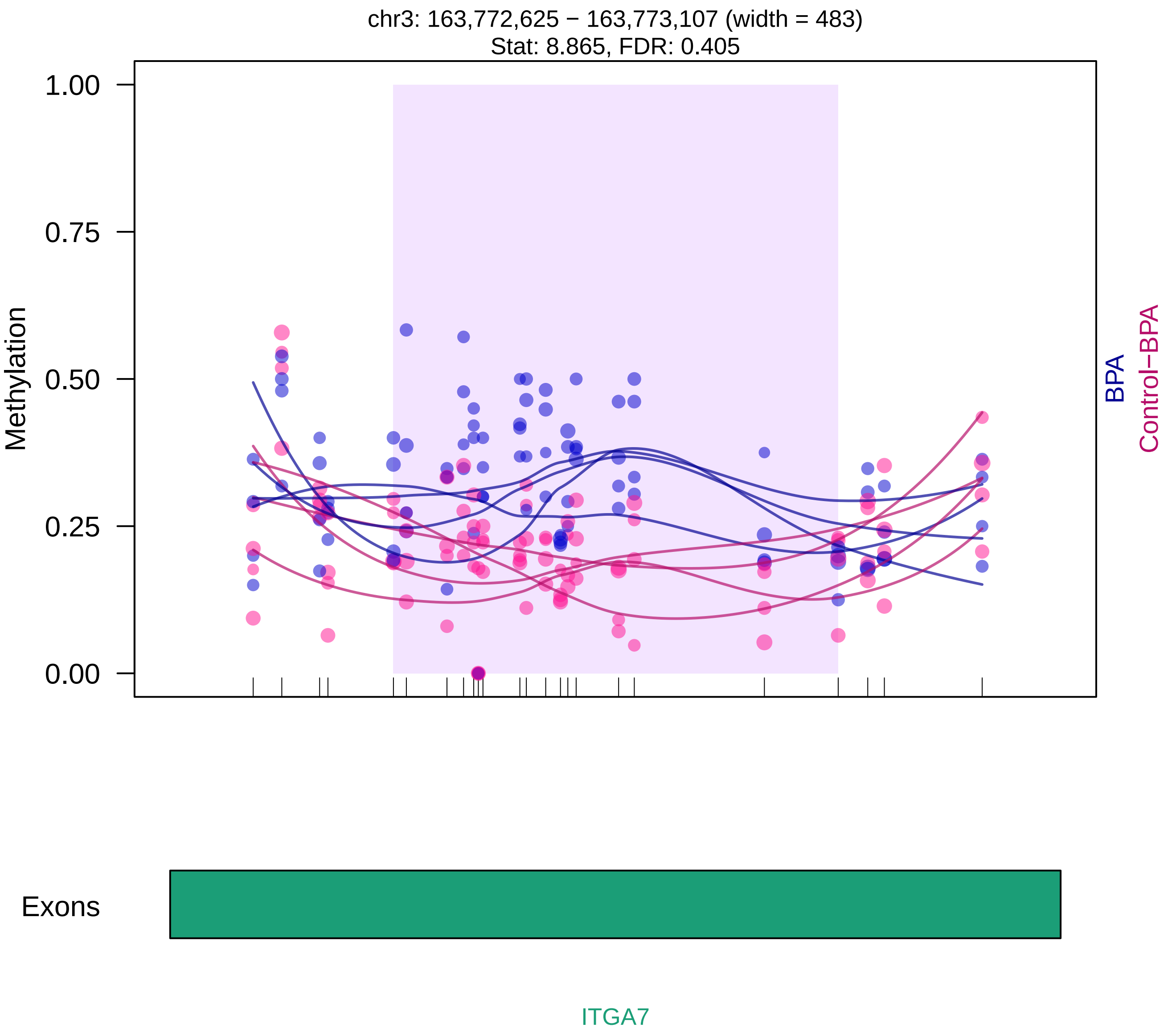
chr3: 163,772,625 − 163,773,107 (width = 483)
Stat: 8.865, FDR: 0.405
1.00
0.75
0.50
0.25
0.00
Methylation
BPA
Control−BPA
Exons
ITGA7

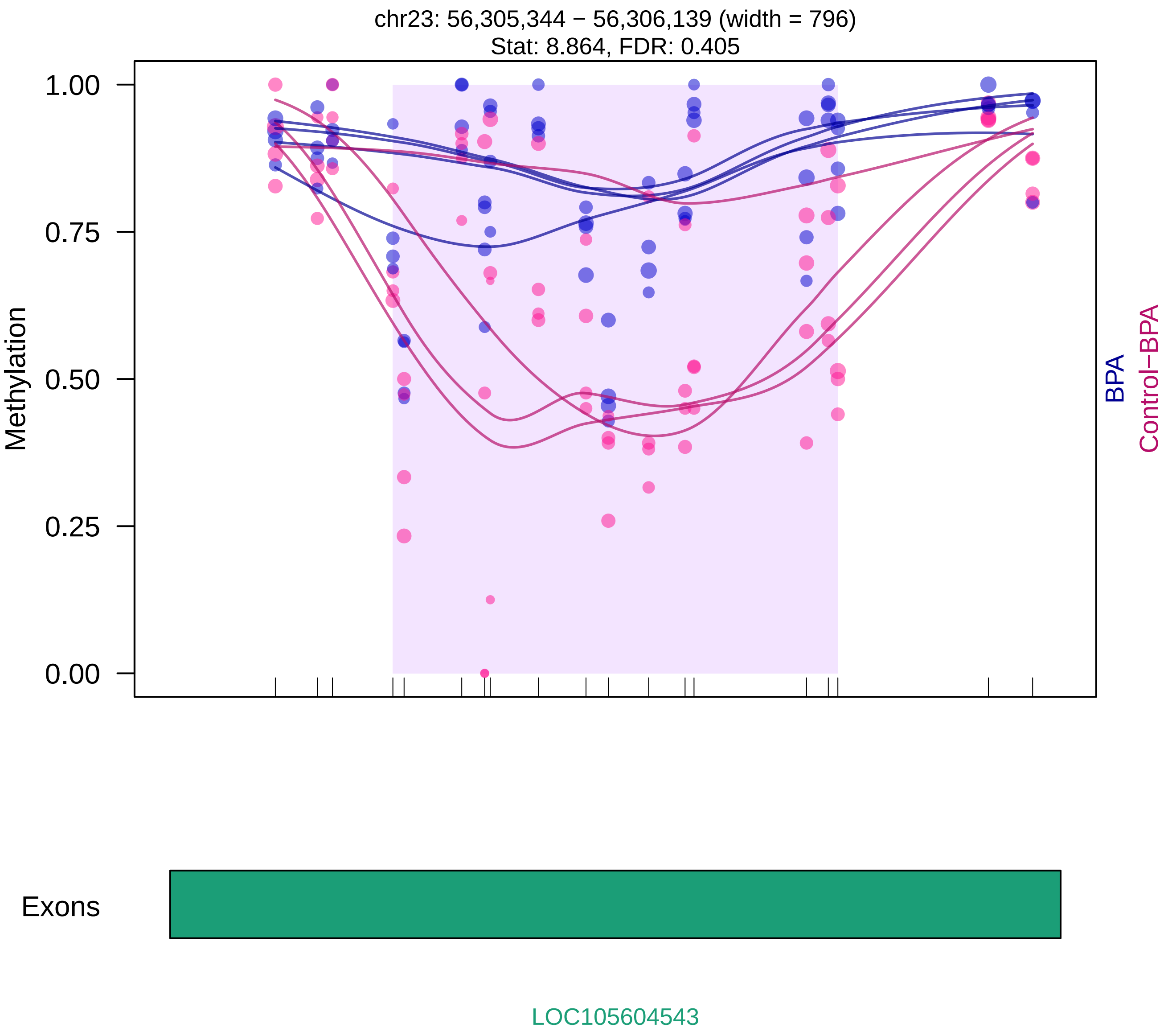

chr23: 56,305,344 − 56,306,139 (width = 796)
Stat: 8.864, FDR: 0.405
Methylation
1.00
0.75
0.50
0.25
0.00
BPA
Control−BPA
Exons
LOC105604543

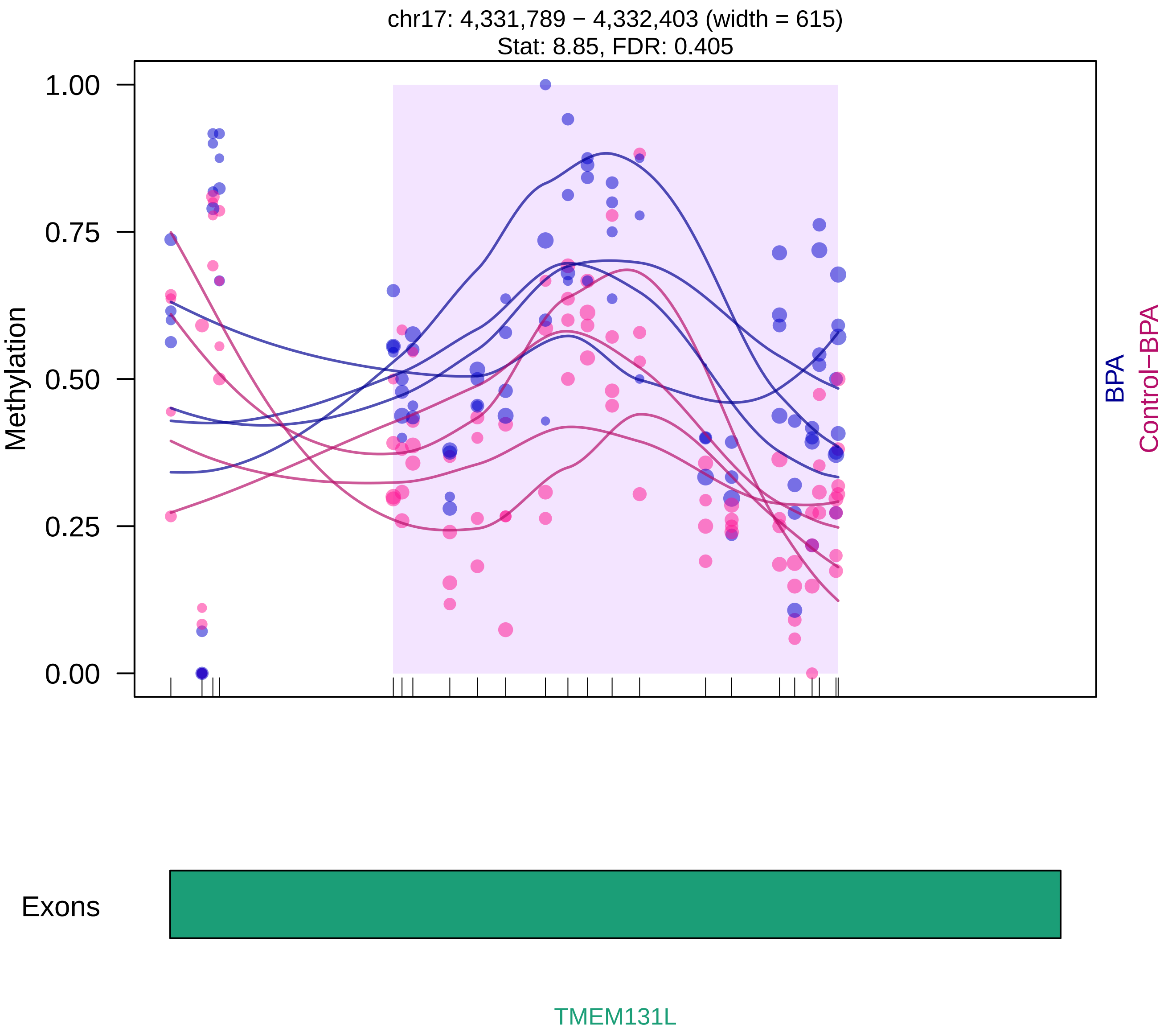
chr17: 4,331,789 − 4,332,403 (width = 615)
Stat: 8.85, FDR: 0.405
Methylation
1.00
0.75
0.50
0.25
0.00
BPA
Control−BPA
Exons
TMEM131L

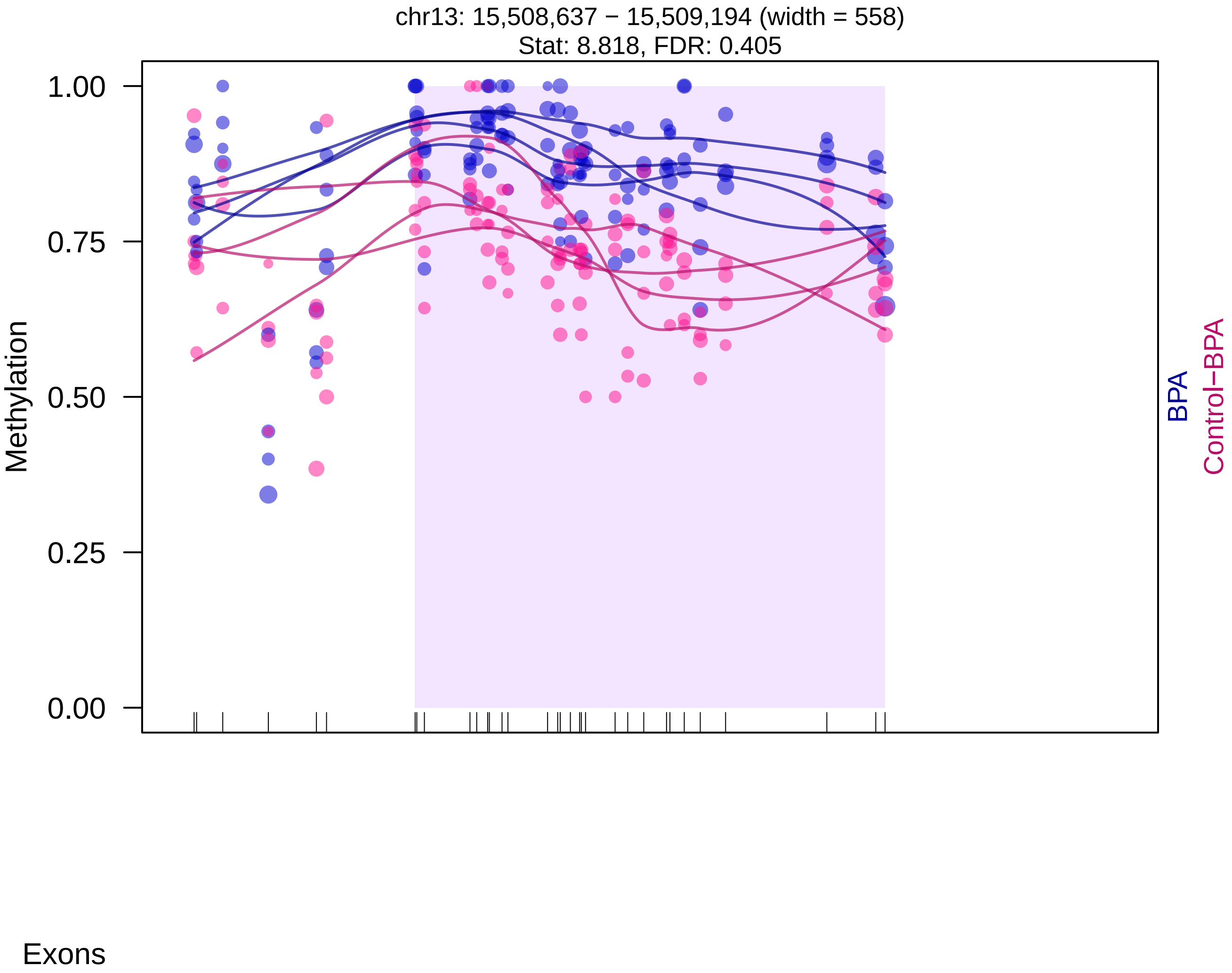

chr13: 15,508,637 − 15,509,194 (width = 558)
Stat: 8.818, FDR: 0.405
Methylation
1.00
0.75
0.50
0.25
0.00
BPA
Control−BPA
Exons

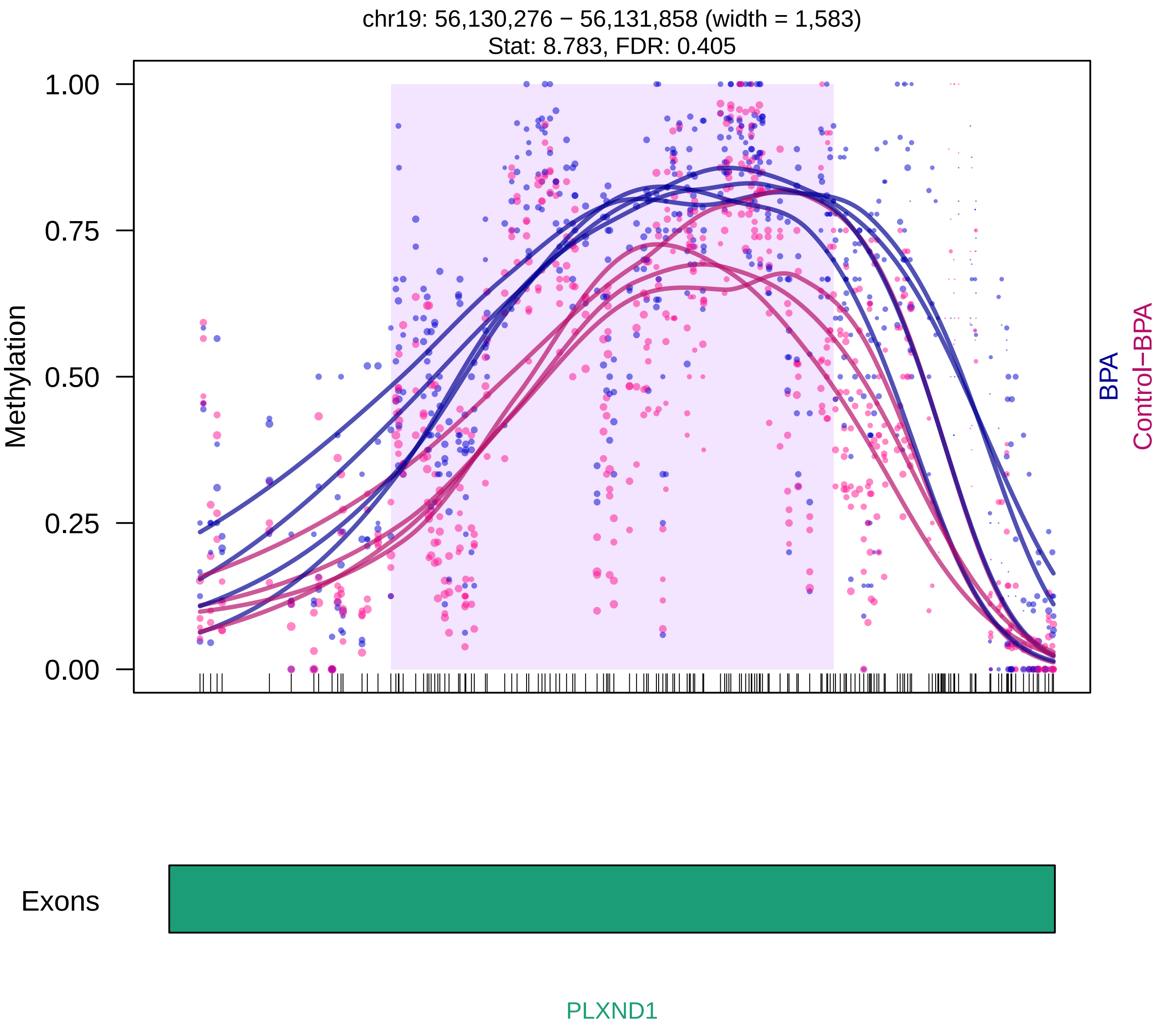
chr19: 56,130,276 − 56,131,858 (width = 1,583)
Stat: 8.783, FDR: 0.405
Methylation
1.00
0.75
0.50
0.25
0.00
BPA
Control−BPA
Exons
PLXND1
