# Supplementary figures and images for: Developmental programming: Differing impact of prenatal testosterone and prenatal bisphenol-A -treatment on hepatic methylome in female sheep
Source: Mol Cell Endocrinol. Author manuscript; Available in PMC 2025 Dec 1. (PMC12668269; doi:10.1016/j.mce.2025.112655)

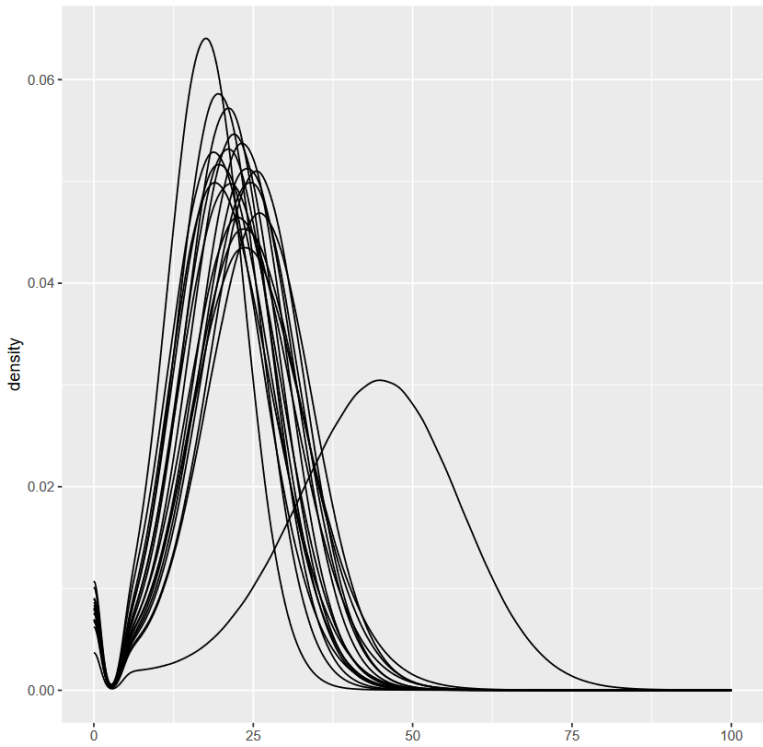

Supplement: Supplementary Figure S1 [file NIHMS2117496-supplement-Supplementary_Figure_S1.pdf]

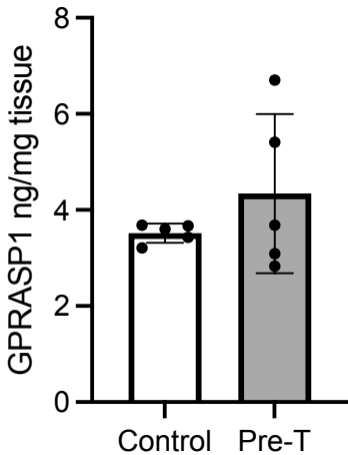

Supplement: Supplementary Figure S2 [file NIHMS2117496-supplement-Supplementary_Figure_S2.pdf]

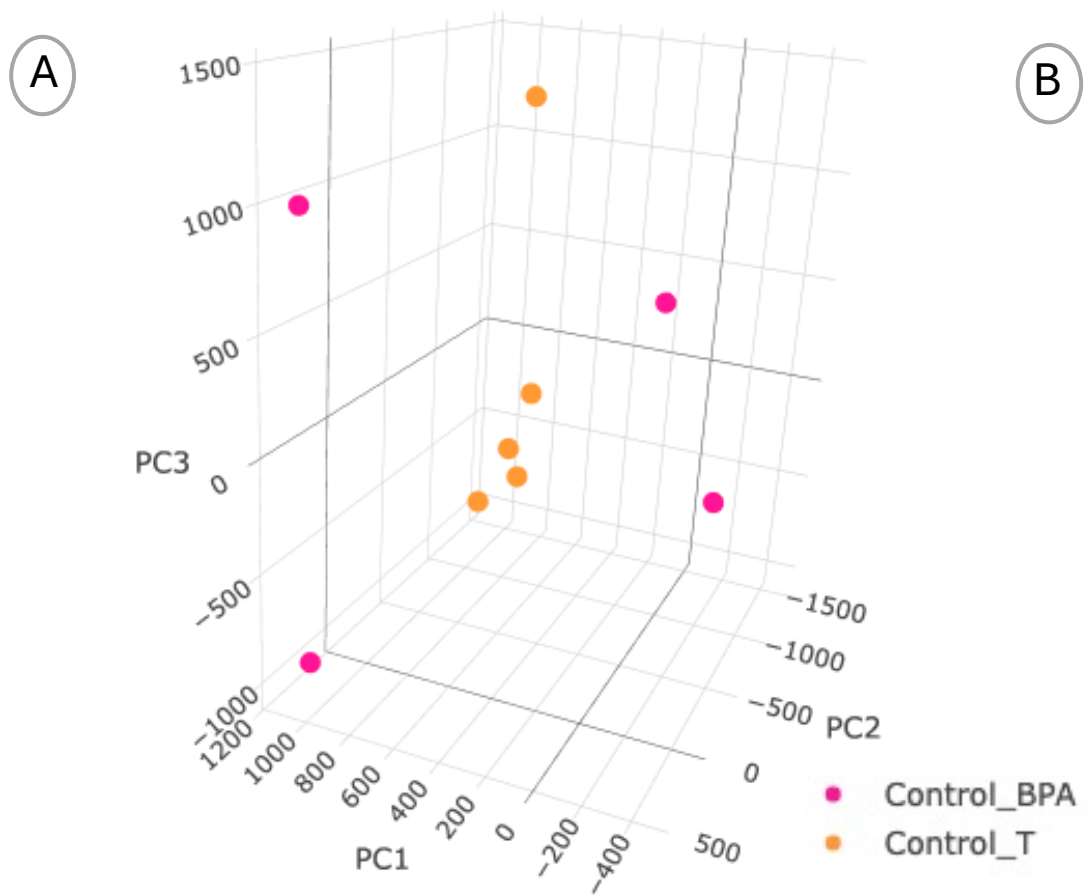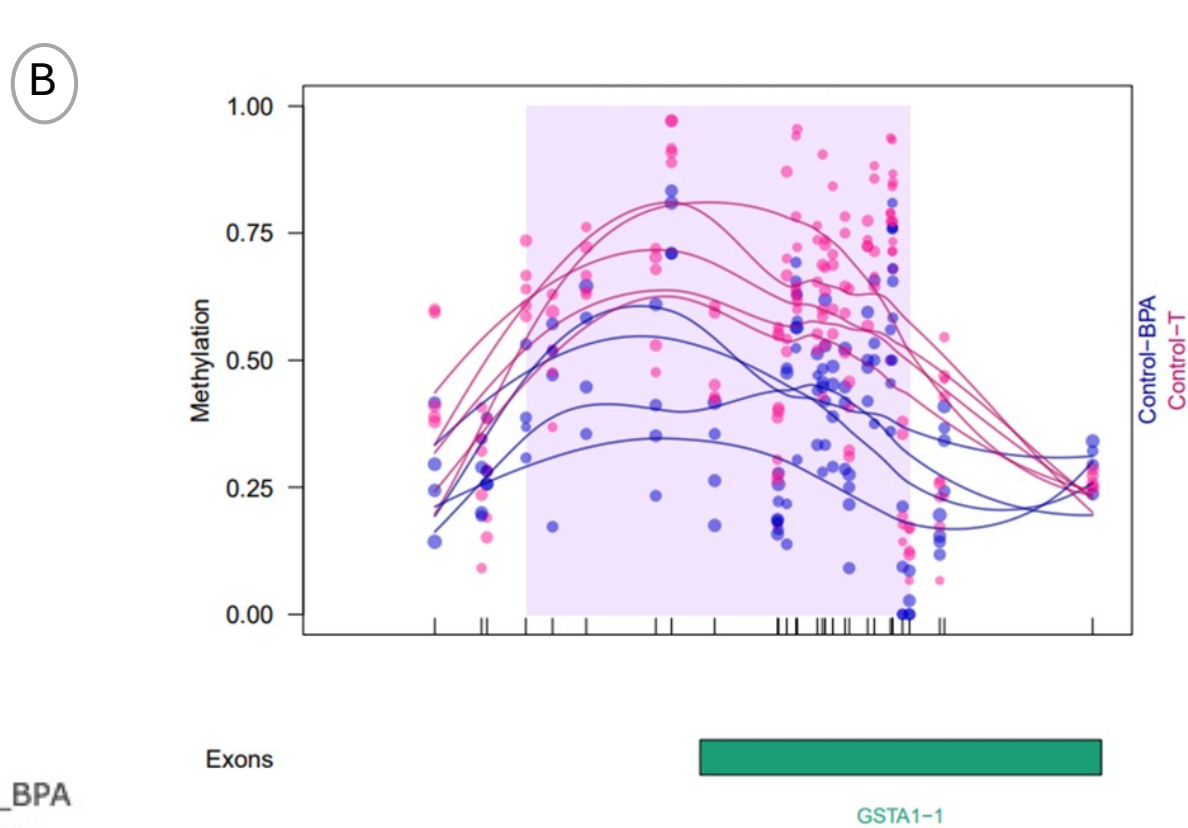

Supplement: Supplementary Figure S3 [file NIHMS2117496-supplement-Supplementary_Figure_S3.pdf]
